# Toxicants Released from Cigarette Butt and Other Tobacco and Nicotine Product Waste and Their Ecotoxicological Impact: A Systematic Review with One Health Perspectives

**DOI:** 10.3390/toxics14100846

**Published:** 2026-09-23

**Authors:** Andrea Moscato, Paola Rapisarda, Margherita Ferrante, Eloise Pulvirenti, Eliana Pellegrino, Luca Amato, Marco Palella, Giuseppe La Torre, Maria Fiore

**Affiliations:** 1 School of Specialization in Hygiene and Preventive Medicine, Department of Medical, Surgical Sciences and Advanced Technologies “GF Ingrassia”, University of Catania, Via S. Sofia 87, 95123 Catania, Italy; moscatoandrea99@gmail.com (A.M.); amatoluc@hotmail.it (L.A.); 2 Department of Medical, Surgical Sciences and Advanced Technologies “GF Ingrassia”, University of Catania, Via S. Sofia 87, 95123 Catania, Italy; paola.rapisarda@phd.unict.it (P.R.); marfer@unict.it (M.F.); eloise.pulvirenti@phd.unict.it (E.P.); elipellegrino90@gmail.com (E.P.); 3 Complex Unit of Public Health, Epidemiologic Games and Preventive Medicine, Provincial Health Authority (ASP) of Catania, Via Santa Maria La Grande 5, 95124 Catania, Italy; markopa92@hotmail.it; 4 Department of Public Health and Infectious Diseases, Sapienza University of Rome, Piazzale Aldo Moro 5, 00185 Rome, Italy; giuseppe.latorre@uniroma1.it

**Keywords:** cigarette butts, tobacco and nicotine product waste, cellulose acetate, microplastics, nanoplastics, ecotoxicology, environmental pollution, One Health

## Abstract

Cigarette butt waste is a widespread form of tobacco-related litter and an increasingly recognized source of environmental contamination. This systematic review synthesized evidence on the environmental impacts of cigarette butts and other tobacco and nicotine product waste, including contaminant release, plastic-derived particle generation, affected environmental matrices, ecotoxicological effects, dose–effect relationships, and ecological and human health risk assessment within a One Health framework. Following PRISMA guidelines and PROSPERO registration, PubMed, Scopus, and Web of Science were systematically searched. Forty-seven studies were included. Cigarette butts and related waste were found to release diverse contaminants, including metals, nicotine, PAHs, VOCs, BTEX, pesticides, phenolic compounds, aromatic amines, and cellulose-acetate-derived micro- and nanoplastics. Nicotine emerged as a prominent contaminant, with rapid release and high modelled ecological risk reported in some studies. Freshwater and marine environments were most frequently investigated, while soils, urban environments, stormwater systems, landfills, and waste-management pathways were also represented. Experimental studies reported mortality, growth inhibition, oxidative stress, reproductive and developmental impairment, genotoxicity, metabolic and behavioral alterations, and community-level effects. Evidence on emerging nicotine product waste remained limited and was constrained by differences in product design, terminology, characterization, and experimental scenarios. Overall, these wastes represent hybrid chemical–particulate environmental stressors, although their substantial heterogeneity limits broader generalization.

## 1. Introduction

Cigarette butts (CBs) represent the most ubiquitous form of anthropogenic litter collected globally, with billions of units discarded into the environment annually, posing a severe threat to global ecosystems [1]. Manufactured predominantly from cellulose acetate, a synthetic plastic that is photodegradable but highly persistent, CBs act as complex chemical matrices that retain and subsequently release a myriad of toxic compounds generated during tobacco combustion [1]. Hazardous substances that compose CBs include polycyclic aromatic hydrocarbons (PAHs), heavy metals, nicotine, and plasticizers [2]. Consequently, post-consumption CB waste constitutes a hazardous point-source of environmental contamination rather than mere aesthetic litter [3]. When discarded into the environment, these residues undergo weathering processes, leading to the leaching of their chemical cargo into urban and natural ecosystems [3,4]. Freshwater and marine systems are particularly relevant because CBs can rapidly release soluble compounds into water and because discarded butts from urban and coastal settings may be transported through runoff into rivers, lakes, beaches, and marine ecosystems. Terrestrial environments are also of concern, as CBs can accumulate in soils, parks, streets, and green areas, where they may interact with plants, soil organisms, and microbial communities [5,6]. Ecotoxicological research has demonstrated adverse effects of CB leachates in various biological models, such as aquatic organisms and plants [7,8]. Concurrently, the paradigm of environmental health has evolved with the One Health approach, recognizing that ecosystems, animal populations, and human health are intrinsically linked [9].

Although conventional cigarette butts remain by far the most extensively investigated form of tobacco product waste, recent years have witnessed the emergence of additional waste streams associated with electronic nicotine delivery systems (ENDS), heated tobacco products, and alternative filter technologies [10,11]. These products differ in material composition, design, and chemical profile and may therefore release distinct mixtures of contaminants into the environment. Although only a limited number of studies have investigated these emerging waste types, the widespread global use of electronic cigarettes and other emerging nicotine products highlights the need to consider their post-consumer waste alongside conventional cigarette butts when evaluating the environmental consequences of tobacco and nicotine product waste [12]. Importantly, the safety of electronic cigarettes for users and bystanders and the environmental impacts associated with discarded e-cigarette products and residues represent distinct issues and should be evaluated separately.

Despite growing attention to tobacco product waste, the available evidence remains heterogeneous in terms of environmental matrices, exposure conditions, contaminants, biological models, and assessed outcomes [13], while its integrated synthesis within a One Health framework remains limited. Accordingly, this systematic review aimed to comprehensively evaluate the environmental contamination and potential health implications associated with cigarette butts and related tobacco and nicotine product waste. Specifically, we sought to: (i) characterize the types of tobacco-related waste and environmental matrices investigated; (ii) synthesize evidence on the release, occurrence, persistence, and environmental behavior of chemical contaminants and plastic-derived particles; (iii) evaluate the biological and ecotoxicological effects reported across terrestrial, freshwater, and marine organisms and ecosystems, including concentration- and time-dependent responses; (iv) summarize available ecological and human health risk assessments; and (v) integrate these findings with evidence from environmental monitoring and life cycle assessment to identify major knowledge gaps and implications from a One Health perspective.

## 2. Materials and Methods

### 2.1. Protocol and Reporting Framework

The systematic review was conducted in accordance with the PRISMA 2020 guidelines and followed an a priori protocol that predefined the objectives, eligibility criteria, search strategy, study-selection process, data-extraction procedures, and quality-assessment methods [14]. The protocol was prospectively registered in the International Prospective Register of Systematic Reviews (PROSPERO, CRD 420261446230) [15,16].

### 2.2. Eligibility Criteria and PECO Framework

The eligibility criteria were primarily guided by the Population, Exposure, Comparator, and Outcomes (PECO) framework [17], which was applied to the main review question concerning the environmental release of contaminants from cigarette butts and other tobacco and nicotine product waste and their associated ecotoxicological effects. Specifically, the population (P) comprised aquatic and terrestrial organisms, biological communities, and environmental matrices potentially affected by tobacco- and nicotine-product waste; the exposure (E) included post-consumption cigarette butts, either whole or fragmented, cigarette butt leachates, cigarette-filter-derived particles, and other tobacco and nicotine product waste; the comparator (C), when applicable, consisted of unexposed or control conditions, reference matrices, or different exposure levels; and the outcomes (O) included contaminant release and occurrence, particle generation, bioaccumulation, and biological or ecotoxicological effects across different levels of biological organization.

Given the broader scope of the review and the heterogeneity of the eligible study designs, additional eligibility criteria were defined to capture evidence from environmental monitoring and exposure assessment, ecological and human health risk assessment, and life cycle assessment studies. Accordingly, studies were eligible if they investigated cigarette butts or other tobacco and nicotine product waste and assessed contaminant release, environmental occurrence, fate or persistence, quantitative environmental pollution burden, plastic-derived particle generation, biological or ecotoxicological effects, environmental exposure, ecological or human health risks, or waste-related environmental impacts. Environmental monitoring studies focusing on tobacco-related litter were considered eligible when litter occurrence or abundance was quantitatively characterized using explicitly defined environmental pollution metrics or indices, such as litter density or pollution indices, thereby providing a quantitative assessment of the environmental pollution burden.

Ecotoxicological studies were eligible when they reported quantitative biological endpoints, including mortality, growth inhibition, reproductive or developmental impairment, oxidative stress, genotoxicity, metabolic or behavioral alterations, or community-level responses. Studies investigating unused or virgin cigarette filters were included only when they assessed environmentally relevant contaminant or particle release or provided direct comparisons with post-consumption cigarette butts.

Studies focusing exclusively on cigarette smoke or smoking exposure, filter design, performance or ventilation, or alternative filter materials without direct environmental relevance were excluded. Studies reporting only the presence, occurrence, or abundance of tobacco-related litter without quantitative characterization through environmental pollution metrics or indices, and without contaminant, exposure, risk, or toxicological assessment, were excluded. Studies reporting wildlife ingestion without contaminant, exposure, risk, or toxicological assessment were also excluded. Only peer-reviewed articles published in English were considered eligible.

These predefined eligibility criteria were systematically applied during full-text assessment, and a specific reason for exclusion was recorded for each article excluded at this stage.

### 2.3. Search Strategy

A comprehensive literature search was conducted in PubMed, Scopus, and Web of Science for studies published from January 2020 to June 2026. These databases were selected to provide complementary coverage of the multidisciplinary evidence relevant to the review: PubMed primarily covers biomedical and toxicological research, whereas Scopus and Web of Science provide broad coverage of environmental sciences, ecotoxicology, pollution, and other interdisciplinary fields. Their combined use was therefore considered appropriate to capture the different components of the One Health framework addressed in this review. The 2020 cutoff was selected to provide an updated synthesis of the most recent evidence, building on the literature already covered by previous reviews. The search strategy combined free-text terms related to cigarette butt and tobacco product waste, contaminant release, ecotoxicology, environmental pollution, and risk assessment. The core search strategy was: (“cigarette butt” OR “cigarette filter” OR “tobacco product waste” OR “cigarette waste”) AND (leachate OR toxicant* OR pollutant* OR contaminant* OR “heavy metal” OR PAH OR phthalate* OR “cellulose acetate” OR microplastic) AND (ecotoxicology OR ecotox OR “environmental pollution” OR “risk assessment” OR “environmental risk” OR “One Health”). The search strategy was adapted to the syntax and search fields of each database. No geographical restrictions were applied. In addition, the reference lists of all included studies were manually screened to identify potentially eligible publications not retrieved through the database searches.

### 2.4. Study Selection

All retrieved records were imported into Rayyan (web-based systematic review platform, Rayyan System Inc., Cambridge, MA, USA), which was used to manage the screening process and ensure a transparent and reproducible workflow [18]. After automatic and manual deduplication within the platform, the study selection proceeded through the standard Rayyan workflow, beginning with blind title and abstract screening conducted independently by two reviewers. Rayyan’s blinding feature allowed each reviewer to classify records without influence from the other, thereby reducing selection bias. Conflicts generated during this phase were subsequently unblinded and resolved through discussion, and when necessary, by consulting a third reviewer. Studies deemed potentially eligible were then subjected to full-text assessment within Rayyan, where inclusion and exclusion decisions were recorded along with justifications. The entire selection process, from initial import to final inclusion, is summarized in the PRISMA flow diagram. Study selection was independently performed by two reviewers (A.M and P.R), and any disagreements were resolved by consensus with a third reviewer (M.F.).

### 2.5. Data Extraction and Classification of Study Characteristics

Study characteristics were classified using a predefined operational framework developed by the review authors. Study design was assigned at a general methodological level using the categories laboratory experimental study, in vivo laboratory experimental study, in vitro laboratory experimental study, field observational study, combined field and laboratory study, and environmental risk assessment study, the latter being reserved for investigations primarily based on risk modelling without a substantive experimental component. The experimental model was classified separately to describe how each study was conducted and included laboratory leaching experiment, laboratory sorption experiment, laboratory ecotoxicological bioassay, whole-cigarette butt exposure bioassay, mesocosm exposure experiment, and field survey. Laboratory ecotoxicological bioassays encompassed exposure to cigarette-butt leachates, extracts, fibres, microplastics, nanoplastics, or other cigarette-butt-derived contaminants.

Variables measured in each study were reported as outcomes assessed, encompassing chemical concentrations, leaching parameters, biological and ecotoxicological endpoints, and risk-assessment metrics such as toxic equivalency and risk quotients. Each study was also classified according to the One Health domain(s) directly addressed by its objectives and measured outcomes. Animal health included biological or toxicological effects in animal organisms; plant health included biological, physiological, or toxicological effects on plant organisms, including terrestrial plants and aquatic macrophytes; environmental health encompassed environmental contamination, pollutant release, occurrence, fate or transport, littering, waste degradation or persistence, and ecosystem- or community-level effects; and human health included quantitative assessments of human exposure or potential health risk associated with contaminants released from cigarette butts or other tobacco and nicotine product waste, including carcinogenic and non-carcinogenic risk estimates. No study was classified exclusively within the human health domain; rather, human health was represented only in multi-domain studies in combination with environmental health.

Domain assignment was based on study objectives, experimental or observational models, and measured outcomes rather than solely on the environmental matrix used for sampling, leaching, or exposure. Thus, the use of an environmental medium did not, by itself, justify assignment to the environmental health domain. The domains were non-mutually exclusive, and individual studies could therefore be assigned to more than one domain. Studies directly addressing a single domain were classified as single-domain, whereas those directly addressing two or more domains were classified as multi-domain. Accordingly, multi-domain studies were represented in each relevant domain-specific category as well as being identified separately as multi-domain studies.

One Health domains were not extracted from the original articles but were independently assigned by the review authors using an operational framework adapted from the One Health High-Level Expert Panel (OHHLEP) definition of One Health, with particular emphasis on the environmental dimension relevant to cigarette butt pollution [19]. For the purposes of this review, the One Health framework was operationalized into four interconnected domains: human health, animal health, plant health, and environmental health. The data-extraction spreadsheet also captured the toxic substances investigated, ecotoxicological outcomes, key findings, quantitative measures when available, and study limitations. Data extraction was conducted independently by three reviewers (A.M., E.P., L.A.), and disagreements were resolved by consensus with a fourth reviewer (M.F.).

### 2.6. Risk of Bias Assessment

The methodological quality of the included studies was assessed according to study design. Experimental in vivo and in vitro toxicological studies were evaluated using the Toxicological Data Reliability Assessment Tool (ToxRTool) [20]. Environmental monitoring and exposure-assessment studies, human health risk assessment (HHRA) studies, life cycle assessment (LCA) studies, and laboratory experimental studies that were neither in vivo nor in vitro were evaluated using a review-specific methodological quality checklist developed a priori for this review. The checklist integrated methodological principles from internationally recognized frameworks, including the Office of Health Assessment and Translation (OHAT) Risk of Bias guidance developed by the U.S. National Toxicology Program, the U.S. Environmental Protection Agency (USEPA) Risk Assessment Guidance for Superfund (RAGS) for Human Health Evaluation, and the International Organization for Standardization (ISO) 14040 and ISO 14044 standards for Life Cycle Assessment [21].

The checklist comprised ten predefined domains: (i) clarity of study objectives; (ii) appropriateness of the study design; (iii) adequacy and representativeness of the sampling strategy; (iv) exposure characterization; (v) validity and description of analytical methods, including method validation and quality assurance/quality control (QA/QC), where relevant; (vi) quality and reliability of input data; (vii) transparency and scientific justification of model assumptions; (viii) uncertainty and sensitivity analyses, where applicable; (ix) appropriateness and reporting of statistical or modelling analyses; and (x) consistency between the reported results, acknowledged limitations, and authors’ conclusions (Table S1).

Each domain was rated as low methodological concern (2 points), moderate methodological concern (1 point), or high methodological concern (0 points). For the analytical methods domain, the highest score required both adequate methodological description and evidence of appropriate validation and/or QA/QC procedures. For the sampling strategy domain, the highest score required a clearly described and adequately representative sampling approach, whereas appropriate but less representative sampling strategies were rated as moderate methodological concern.

Domains 7 and 8 were applied only to studies that directly developed or implemented quantitative modelling or risk-modelling frameworks involving explicit assumptions. Descriptive calculations, pollution indices, concentration estimates, or the use of model-derived parameters from previously published sources were not considered sufficient to classify a study as a modelling study. When externally derived model outputs or parameters were used without direct implementation of the underlying model, their appropriateness was evaluated under the input data quality domain. For non-modelling studies, Domains 7 and 8 were rated as not applicable (N/A) and excluded from the total score.

For Domain 8, the highest score was assigned when a formal and sufficiently comprehensive uncertainty and/or sensitivity analysis was performed, whereas a moderate score was assigned when uncertainty in model inputs, assumptions, or outputs was only partially or qualitatively evaluated. General acknowledgement of study limitations was not considered an uncertainty analysis and was assessed under Domain 10.

For studies in which all ten domains were applicable, total scores ranged from 0 to 20, and methodological quality was classified as high (17–20), moderate (13–16), or low (≤12). For non-modelling studies, total scores ranged from 0 to 16 and were classified as high (14–16), moderate (10–13), or low (≤9). In mixed-design studies combining observational/environmental and in vivo or in vitro experimental components, the review-specific checklist was applied only to the observational/environmental component, while the experimental component was assessed separately using the corresponding ToxRTool module.

Each study was independently assessed by two reviewers (M.P. and P.R.), and disagreements were resolved by consensus with a third reviewer (G.L.T.).

## 3. Results

The literature search identified 261 records from three electronic databases. After removing 121 duplicate records, 140 articles were screened by title and abstract. At this stage, 78 records were excluded, including 14 reviews and 64 records not relevant to the review question. A total of 62 articles underwent full-text assessment, of which 15 were excluded because they addressed littering or monitoring only (*n* = 5), smoke chemistry or filter ventilation (*n* = 3), alternative or biodegradable filters without direct environmental relevance (*n* = 3), or wildlife ingestion only (*n* = 4). Consequently, 47 studies were included in the qualitative synthesis (Figure 1).

### 3.1. Characteristics of the Included Studies According to One Health Domains

#### 3.1.1. Environmental Health Domain

A total of 22 studies were classified within the environmental health domain. Published between 2020 and 2026, they were conducted in Germany, Iran, Italy, Brazil, France, China, India, the United States, and Indonesia, and included laboratory experimental, in vivo and in vitro experimental, field observational, and combined field–laboratory designs. The studies investigated the occurrence and distribution of cigarette butt waste, contaminant and particle release, leaching and sorption processes, ecological risk, and community-level biological responses across freshwater and marine waters, urban and coastal environments, recreational beaches, and stormwater systems.

Chemical investigations assessed nicotine, PAHs, BTEX, PCBs, OCPs, aromatic amines, phenolic compounds, and metals, while other studies examined cigarette-filter-derived microfibers, microplastics, and nanoplastics. Laboratory experiments used different aqueous media and exposure durations to characterize contaminant release and behavior, with several studies also assessing ecological risk through risk quotients, toxic equivalency, pollution indices, hazardous concentrations, or related metrics. Field and combined field–laboratory studies quantified cigarette butt abundance and spatial distribution and, in some cases, coupled environmental surveys with chemical characterization or controlled leaching experiments.

Community-level investigations evaluated phytoplankton abundance, richness and composition, as well as cyanobacterium–chytrid host–parasite responses, including growth, infection dynamics, oxidative and metabolic biomarkers, and microcystin concentrations. Overall, the environmental health evidence encompassed environmental occurrence, chemical and particulate contamination, contaminant fate and release, ecological risk, and community-level responses (Table 1).

#### 3.1.2. Plant Health Domain

A total of three studies were classified within the plant health domain. Published between 2022 and 2025, they were conducted in the United Kingdom, Poland/Czech Republic, and Türkiye and used in vivo or in vitro laboratory experimental designs. The investigated plant models included the freshwater macrophyte Lemna minor, Sinapis alba, Hordeum vulgare, and *Allium cepa*.

The studies evaluated exposure to smoked conventional cigarette butts or their leachates, unsmoked cigarette filters, and, in one study, used disposable e-cigarettes and extracted e-liquid. Outcomes included biomass, root length, frond number, chlorophyll content, seed germination and germination indices, as well as cytotoxic and genotoxic endpoints, including mitotic activity, chromosomal abnormalities, micronuclei, and total chromosomal damage. Exposure periods ranged from 72 h to 14 days, with cigarette-butt leachates tested at different concentrations and leaching durations (Table 2).

#### 3.1.3. Animal Health Domain

A total of 12 studies were classified within the animal health domain. Published between 2020 and 2025, they were conducted in Egypt, Iran, South Korea, Portugal, Brazil, Slovenia, the United Kingdom, China, and India. All used in vivo laboratory experimental designs and investigated terrestrial, freshwater, and marine organisms, including earthworms, crustaceans, rotifers, molluscs, planarians, insects, and fish.

Experimental exposures involved smoked conventional cigarette butts or filters, unsmoked filters or cigarette butts, cigarette-butt leachates or elutriates, and cellulose-acetate-derived micro- or nanoplastics. Exposure media included artificial or agricultural soil, freshwater and standardized freshwater media, filtered rainwater, and artificial seawater. The studies assessed acute and chronic or sub-chronic effects across multiple biological endpoints, including mortality and survival, growth, reproduction, behaviour, feeding and locomotor activity, oxidative stress and antioxidant responses, neurotoxicity, metabolic and mitochondrial alterations, genotoxicity, histopathological changes, bioaccumulation, and molecular responses.

Overall, the animal health studies evaluated the effects of cigarette-butt leachates and cigarette-filter-derived particulate materials across terrestrial, freshwater, and marine animal models, using exposure periods ranging from acute assays of 24–48 h to longer-term experiments lasting several weeks, including assessment of post-exposure recovery in one study (Table 3).

#### 3.1.4. Multi-Domain Studies and One Health Integration

A total of 12 studies were classified as multi-domain, addressing two or more predefined One Health domains. Published between 2021 and 2026, they were conducted in Spain, Italy, Chile, Bosnia and Herzegovina, Sweden, India, and Iran. Study designs included laboratory experimental, in vivo and in vitro laboratory experimental, combined field–laboratory, and environmental risk assessment studies. The most frequent combination was environmental health + animal health, while other studies addressed environmental health + human health, environmental health + plant health, or environmental health + animal health + plant health.

The multi-domain studies investigated contaminant and microfiber release, cigarette-butt degradation, environmental occurrence and littering, ecotoxicological effects, and ecological or human health risks. Experimental and observational approaches encompassed freshwater and marine environments, soils, coastal and urban settings, landfill systems, and modelled environmental compartments. Biological outcomes included mortality, immobilization, growth, reproduction and development, phytotoxicity, biomarker responses, bioaccumulation, and community-level effects, while risk-oriented studies assessed ecological and human health metrics, including hazard quotients, carcinogenic and non-carcinogenic risks, and life-cycle environmental impacts.

Overall, these studies integrated environmental contamination or waste-related processes with direct assessments of animal, plant, or human-health-related outcomes. Tobacco products investigated included smoked and unsmoked conventional cigarette butts or filters and, in some studies, electronic or heated tobacco product waste (Table 4).

### 3.2. Methodological Quality of the Included Studies

#### 3.2.1. Methodological Quality of In Vitro Studies

Seven studies included an in vitro component and were assessed using ToxRTool. All seven studies were classified as reliable without restriction, with total scores ranging from 16/18 to 18/18. Bonanomi et al. (2026) and Lazo et al. (2024) achieved the maximum score (18/18) [58,59], while Guttmann et al. (2024), Jakimiuk et al. (2022), Michael et al. (2022), and Piccardo et al. (2021) scored 17/18 [33,36,44,65]. Martinez-Ruiz et al. (2026) received the lowest score (16/18) [35], but remained within the reliable without restriction category. Overall, the in vitro evidence showed consistently high methodological reliability, with no study classified as reliable with restrictions or not reliable (Table S2a).

#### 3.2.2. Methodological Quality of In Vivo Studies

Twenty studies included an in vivo component and were assessed using ToxRTool. Sixteen studies were classified as reliable without restriction, whereas four were classified as reliable with restriction; no study was classified as not reliable. Total scores ranged from 16/21 to 20/21. Cho et al. (2025), Dolar et al. (2025), Olah-Kovacs et al. (2025), and Soleimani et al. (2023) achieved the highest score (20/21) [48,51,55,66].

The four studies classified as reliable with restriction were de Arragao Ferreira-Griz et al. (2026), Green et al. (2020), Green et al. (2023), and Sadic et al. (2025), with scores ranging from 16/21 to 17/21 [43,45,50,52]. Overall, the in vivo evidence showed predominantly high reliability, with 80% (16/20) of the assessed studies classified as reliable without restriction (Table S2b).

#### 3.2.3. Methodological Quality of Other Environmental and Experimental Studies

The review-specific methodological quality checklist was applied to the environmental and laboratory experimental studies that were neither in vivo nor in vitro, including the relevant observational/environmental components of mixed-design studies. Among the 22 studies listed in Table S3, 9 were classified as having high methodological quality, 12 as moderate quality, and 1 as low quality. Scores ranged from 12/16 to 16/16 among non-modelling studies and from 12/20 to 17/20 among studies for which all ten domains were applicable.

The highest score among non-modelling studies was obtained by Yona et al. (2025) (16/16) [42], while El Hadri et al. (2021) and Nongdren et al. (2026) achieved the highest score among studies assessed across all ten domains (17/20) [31,63]. Kouhi et al. (2025) was the only study classified as low methodological quality (12/20) [34]. For mixed-design studies, only the observational/environmental component was considered in this assessment, while in vivo or in vitro components were evaluated separately using the corresponding study-design-specific tool (Table S3).

### 3.3. Main Findings According to One Health Domains

#### 3.3.1. Environmental Health Domain

Studies addressing the environmental health domain documented the release, occurrence, persistence, degradation, and environmental fate of chemical contaminants and particulate materials derived from cigarette butts (CBs) and related tobacco product waste. Chemical analyses identified nicotine and cotinine; polycyclic aromatic hydrocarbons (PAHs); benzene, toluene, ethylbenzene, and xylenes (BTEX); polychlorinated biphenyls (PCBs); organochlorine pesticides; aromatic amines; phenolic compounds; and metals [22,27,68]. Release patterns varied according to chemical class, contact time, smoking status, cigarette-butt component, environmental ageing, and experimental conditions. PAH concentrations progressively increased during prolonged water contact, whereas BTEX compounds were rapidly released, reaching equilibrium within approximately 15 min [24,38].

Nicotine emerged as a prominent chemical contaminant in the available evidence. Dobaradaran et al. (2024) reported aqueous nicotine concentrations ranging from 0.04 to 3.04 mg/L, with release detected within the first 5 min of contact with water and concentrations exceeding predicted no-effect concentrations by several orders of magnitude; the resulting risk quotients indicated high ecological risk (RQ > 10) from the earliest investigated time point [25]. Nongdren et al. (2026), using a life-cycle assessment approach, estimated nicotine release at 2.095 mg per butt and identified nicotine as the dominant contributor to freshwater ecotoxicity during the littering phase, accounting for 93.31% of the estimated impact [63]. Similarly, Tan et al. (2026) reported a nicotine concentration of 744 mg/kg in discarded cigarette-butt waste and estimated that nicotine accounted for 75.15% of cumulative toxicity, identifying it as the principal contributor to acute toxicity [27].

Other chemical constituents also showed contaminant-specific release and risk profiles. PAHs, including benzo(a)pyrene, fluoranthene, anthracene, and naphthalene, were associated with aquatic contamination and, in some studies, concentrations exceeding environmental quality or risk-based thresholds [28,38]. Aromatic amines, including aniline and 2-naphthylamine, and phenolic compounds such as phenol, o-cresol, and 2,4-dimethylphenol also exceeded predicted no-effect concentrations under some experimental conditions [40,68]. Metals including Pb, Cd, Cr, Fe, Cu, Zn, and Mn were detected in cigarette-butt waste or leachates, with release kinetics varying according to the element and environmental conditions [28,29,36,42]. In seawater, for example, Yona et al. (2025) observed an early release peak for Cd, whereas Zn concentrations progressively increased over 30 days, and Fe showed a delayed peak after approximately one week [42]. Overall, these findings indicate that CBs release complex mixtures of chemical contaminants characterized by markedly different release kinetics, persistence, and environmental-risk profiles.

Several studies also documented the release and persistence of cellulose-acetate-derived particulate material. Belzagui et al. (2021) showed sustained release of microfibres from cigarette filters and limited degradation of cellulose acetate after prolonged exposure to aquatic media and natural sunlight [60]. Similarly, Mohammadi et al. (2025) reported a time-dependent increase in microfiber release from cigarette filters over 60 days, with greater release in seawater than in distilled water [57]. Long-term decomposition experiments similarly showed that, although CB mass and toxicity decreased over time, cellulose acetate degradation remained incomplete, and measurable biological effects persisted after up to 10 years [58].

Field investigations documented substantial CB contamination in urban and coastal environments, with abundance varying according to location, land use, season, and sampling approach. Yang et al. (2023) documented CB occurrence across beach, park, and market settings and combined field occurrence data with laboratory characterization of metals, PAHs, and plastic-derived particles [28]. Garshasbi et al. (2026), for example, reported a mean density of 3.71 CBs/m^2^ in an urban stormwater network, with all investigated stations classified as extremely dirty according to the applied Cigarette Butt Pollution Index [37].

Where comparisons among tobacco-product types were available, environmental profiles differed according to product composition. Mihajlović et al. [29] compared conventional cigarette butts with used HEETS tobacco sticks from a heated tobacco product (HTP).

The original study referred to the HEETS residues as “e-cigarette butts”; however, HEETS are consumables of heated tobacco products and should not be classified as electronic-cigarette waste.

Conventional smoked CBs contained higher mean Pb, Cr, and Cd concentrations, whereas Mn was higher in used HEETS tobacco sticks. Metal mobilization in both product types was strongly influenced by pH and contact time [29].

At the community level, dos Santos et al. (2024) reported concentration-dependent reductions in freshwater phytoplankton cell density and species richness, together with progressive alterations in community composition following exposure to cigarette-butt leachate [32].

Overall, environmental health studies characterized tobacco-product waste as a source of both complex chemical mixture and persistent particulate contamination, with contaminat release, environmental behaviour, and persistence varying according to chemical properties, product characteristics, environmental conditions, and ageing (Figure 2).

#### 3.3.2. Animal Health Domain

Studies addressing the animal health domain reported lethal and sublethal effects across terrestrial, freshwater, and marine organisms. Outcomes included mortality and survival, immobilization, growth, reproduction and development, behavioral changes, oxidative stress, neurotoxicity, metabolic and mitochondrial dysfunction, genotoxicity, histopathological alterations, and bioaccumulation. Concentration-dependent responses were common, although effect magnitude and shape differed across species, life stages, exposure durations, and endpoints.

In freshwater gastropods, survival progressively declined with increasing CB-leachate concentration, with juvenile *Bithynia tentaculata* showing greater sensitivity than adults. At 5 CBs/L, fewer than 20% of juveniles survived after 24 h, while behavioral impairment occurred at lower concentrations [52]. Notably, biodegradable cellulose-filter CBs were not less toxic than conventional cellulose-acetate-filter CBs under the tested conditions.

Soleimani et al. [66] observed concentration- and time-dependent acute toxicity in Periophthalmus waltoni, with toxicity consistently following the order smoked CBs > smoked filters > unsmoked filters. The 96 h LC50 values were 1.37, 2.90, and 7.46 CB/L, respectively, and smoked CB leachates contained higher PAH and metal concentrations than the other materials [66]. Concentration-dependent toxicity was also observed in marine foraminifera, with marked species-specific sensitivity and shell decalcification; Rosalina globularis was the most sensitive taxon, with a 48 h LC50 of 1.9 CB/L in smoked-CB leachate corresponding to a nicotine concentration of approximately 3.7 mg/L [30].

Cigarette-filter-derived particles also produced adverse effects. Chronic exposure to CB-derived microfibres in whiteleg shrimp was associated with increased molting activity, reduced cuticle thickness, altered pigmentation, and gill damage [23]. In the marine rotifer Brachionus plicatilis, CB exposure impaired feeding and swimming behaviour and induced oxidative stress, metabolic disruption, and mitochondrial dysfunction, with adverse responses generally increasing with CB concentration [53]. Oxidative and antioxidant-system alterations were also reported in the freshwater snail *Pila virens* following CB exposure [54].

In Daphnia magna, smoked-filter microplastics caused greater acute and chronic toxicity than microplastics from unsmoked filters, including reproductive impairment and changes in oxidative-stress-, endocrine-, and moulting-related gene expression. Notably, nicotine was released from smoked-filter-derived microplastics in the Daphnia gut, suggesting that these particles may also act as carriers of associated chemical contaminants rather than exerting exclusively particle-mediated effects [48]. Similarly, Dolar et al. [51] found that microplastics derived from smoked cigarette filters generally produced stronger physiological and survival effects than particles derived from unsmoked filters across terrestrial and aquatic invertebrates. The authors related the greater toxicity of smoked-filter particles to their associated chemical load, including nicotine and metals, further supporting the combined chemical–particulate nature of cigarette-filter waste [51].

Direct comparisons with alternative cigarette products were less frequent. Piccardo et al. (2021) compared classic and electronic cigarette butts in marine bioassays and reported greater ecotoxicity for classic CBs, particularly smoked classic CBs, whereas electronic cigarette butts were associated with lower hazard classifications under the tested conditions [65].

Overall, the animal evidence showed substantial variability among models but repeatedly identified stronger biological effects for smoked conventional CB- or filter-derived materials than for corresponding unsmoked materials; where direct comparisons with electronic cigarette waste were available, conventional cigarette butts generally produced greater ecotoxicological effects (Figure 3).

#### 3.3.3. Plant Health Domain

Studies addressing the plant health domain evaluated seed germination, root and seedling development, biomass, chlorophyll-related parameters, mitotic activity, and cytogenetic alterations. Responses varied according to plant species, tobacco-product type, exposure concentration, and experimental conditions.

In *Allium cepa*, both smoked CB and unsmoked-filter leachates produced concentration-dependent physiological, cytotoxic, and genotoxic effects. Increasing concentrations progressively reduced root growth and mitotic activity and increased chromosomal abnormalities and micronuclei, with smoked CB leachate generally producing stronger effects than unsmoked-filter leachate [45].

Jakimiuk et al. (2022) reported species-dependent and biphasic responses in Sinapis alba and Hordeum vulgare. Low CB exposures occasionally stimulated germination or root growth, whereas increasing CB concentrations progressively reduced seed germination, germination indices, and root length, particularly in S. alba [44]. Lazo et al. (2024) similarly observed inhibition of germination and seedling growth in Lactuca sativa and Lolium perenne, with L. sativa showing greater sensitivity [59].

A direct comparison between conventional CB waste and electronic-cigarette-related waste was provided by Green et al. [43]. Exposure to one conventional CB/L stimulated Lemna minor biomass, root growth, frond number, and chlorophyll-a, whereas closed disposable e-cigarettes, experimentally opened/disassembled disposable e-cigarettes, and isolated e-liquid significantly reduced frond production. The responses to experimentally opened/disassembled devices and isolated e-liquid were similar, suggesting an important contribution of residual e-liquid under the tested experimental conditions. However, these two treatments represented experimentally modified exposure scenarios rather than exposure to an intact discarded device [43].

Thus, plant responses were not uniformly inhibitory and depended strongly on product type, plant species, and exposure level. The limited comparative evidence indicates that conventional CBs and disposable electronic-cigarette waste may produce qualitatively different biological responses under the specific experimental conditions tested (Figure 4).

#### 3.3.4. Multi-Domain Studies and One Health Integration

Multi-domain studies simultaneously addressed environmental processes and outcomes relevant to animal, plant, or human health. The identified combinations included environmental health + animal health, environmental health + plant health, environmental health + human health, and environmental health + animal health + plant health.

Belzagui et al. [60], classified as environmental health + animal health, directly linked microfiber release and environmental persistence with biological effects in Daphnia magna. The 48 h EC50 decreased from 0.067 SF/L for leachate alone to 0.017 SF/L in the presence of microfibres, while cellulose acetate showed limited degradation, thereby linking particulate persistence with increased biological toxicity [60].

Bonanomi et al. [58], addressing environmental, animal, and plant health, integrated long-term CB decomposition, chemical transformations, microbial-community changes, and ecotoxicological testing. Although toxicity declined during ageing, 10-year-old CBs continued to produce measurable effects, demonstrating the persistence of both environmental and biological impacts over prolonged time periods [58].

Several multi-domain studies provided direct comparisons among tobacco-product types. Oliva et al. (2021) compared smoked and unsmoked conventional-cigarette-filter leachates across marine and freshwater bioassays and generally observed greater toxicity for smoked materials, although concentration–response patterns varied among organisms and were not uniformly monotonic [56]. Piccardo et al. (2021) extended this comparison to materials described by the original authors as “electronic cigarette butts”, reporting higher overall toxicity for classic cigarette butts, particularly smoked classic CBs, while the materials classified as “electronic cigarette butts” in the original study showed lower hazard under the investigated conditions; toxicity was also modified by weathering conditions and duration [65].

Studies incorporating human health did so through quantitative risk assessment rather than direct measurement of human health effects. Mihajlović et al. [29] compared conventional cigarette butts with used HEETS tobacco sticks from a heated tobacco product and found product-specific differences in metal content and leaching, while modelled carcinogenic risk for Cd and Cr exceeded the authors’ acceptable thresholds under the assessed scenarios [29]. Torkashvand et al. (2021) similarly showed that the presence of cigarette butts increased heavy-metal concentrations in landfill leachate and resulted in higher estimated human-health hazard quotients and ecological risk, with freshly smoked cigarette butts producing greater effects than littered cigarette butts [64]. Nongdren et al. (2024) integrated CB littering, predicted surface-water contamination, and human-health risk estimates, whereas Nongdren et al. (2026) extended the assessment to the full cigarette life cycle and compared conventional cellulose acetate filters with biodegradable alternatives [63,67]. In the latter study, nicotine release during the littering phase was estimated at 2.095 mg per butt and accounted for 93.31% of the modelled freshwater ecotoxicity impact, identifying nicotine as a major chemical driver of the environmental burden associated with discarded cigarette waste [63].

In the latter LCA, alternative end-of-life strategies and biodegradable filters showed different environmental profiles from conventional cellulose acetate filters. Filters containing high proportions of flax, hemp, or jute reduced modelled impacts, with ≥90% of jute- or hemp-based filters showing the best endpoint performance among the alternatives evaluated [63].

The waste profiles of conventional cigarettes and emerging nicotine products differed substantially. Conventional CB waste primarily consisted of spent filters containing residual tobacco- and combustion-derived contaminants and cellulose acetate, whereas disposable ENDS generated a more heterogeneous waste stream involving the device and residual e-liquid [43]. Heated tobacco product waste represented a distinct category and should not be conflated with ENDS; in the available evidence, used HEETS tobacco sticks showed metal profiles and leaching behaviour that differed from conventional CBs [29]. Overall, comparative evidence across tobacco-product types was limited but informative. Conventional smoked cigarette butts generally showed greater chemical or ecotoxicological impacts than corresponding unsmoked materials, while the study by Piccardo et al. reported lower hazard for materials described as “electronic cigarette butts”, whereas disposable e-cigarette exposures produced inhibitory plant effects in Lemna minor under the specific experimental conditions tested [43,65]. Biodegradable filter alternatives showed lower modelled life-cycle impacts in one LCA, whereas biodegradable cellulose filters did not reduce leachate toxicity in one freshwater animal study. Given the limited number of direct comparisons and the substantial differences in product design, composition, and resulting waste, findings for one type of tobacco- or nicotine-product waste should not be extrapolated to other product categories without product-specific evidence (Figure 5).

## 4. Discussion

This systematic review shows that cigarette butts (CBs) and related tobacco product waste represent persistent sources of environmental contamination, releasing both chemical contaminants and cellulose-acetate-derived particles. Across the 47 included studies, evidence encompassed environmental contamination, effects on animal and plant models, and, in a smaller subset of multi-domain studies, modelled human-health-related risks. Despite substantial heterogeneity in study designs, exposure conditions, environmental matrices, biological models, and endpoints, recurrent findings included contaminant release, persistence of filter-derived particulate material, and adverse biological responses. Consistent with previous reviews [3,13], these findings confirm the environmental persistence and ecotoxicological relevance of CB waste, while extending previous evidence through more recent data on dose-dependent effects, environmental risk assessment, emerging-tobacco-product waste, and their integration within an explicitly operationalized One Health framework.

The contribution of tobacco-product waste should also be considered relative to other pollution sources. While nicotine represents a characteristic tobacco-derived contaminant, PAHs and metals are not specific to cigarette waste and originate from multiple anthropogenic sources, including combustion, traffic, industrial activities, and urban runoff. Therefore, the available evidence does not allow the contribution of CBs to the overall environmental burden of PAHs and metals to be reliably quantified relative to these other sources. In contrast, cellulose acetate microfibers released from cigarette filters represent a more tobacco-product-specific source of particulate pollution [57,60]. Overall, the environmental relevance of cigarette butts lies in their widespread occurrence and their capacity to simultaneously release chemical contaminants and persistent plastic-derived particles.

The methodological appraisal showed that most individual studies met the reliability or methodological-quality criteria of the study-design-specific assessment tools applied. However, these findings should not be interpreted as indicating high certainty of the overall body of evidence. In particular, some methodological categories were represented by relatively few studies; for example, only seven studies included an in vitro component and were assessed using ToxRTool. Although all seven were classified as reliable without restriction, ToxRTool assesses the methodological reliability of individual studies and does not provide an inferential assessment of the certainty, consistency, or generalizability of the evidence base as a whole. Accordingly, the results of the methodological appraisal should be interpreted primarily as a structured descriptive characterization of the included studies. Limitations related to sampling representativeness, exposure characterization, analytical procedures, input data quality, modelling assumptions, and uncertainty assessment remained relevant in some studies.

The included studies documented the release of multiple contaminants, including nicotine, metals, PAHs, VOCs and BTEX, phenolic compounds, aromatic amines, pesticides, and cellulose-acetate-derived microfibers, microplastics, and nanoplastics [7,25,38,40,62]. These findings support considering CBs as hybrid chemical–particulate contaminants, rather than solely as sources of individual pollutants. Cellulose acetate filters can release persistent particles while simultaneously acting as reservoirs of tobacco- and combustion-derived chemicals [39,48,60]. This dual nature is relevant in the broader context of environmental microplastic exposure [46,69]. However, most studies did not distinguish the relative contribution of particles from that of associated chemicals; therefore, additive or interactive effects should be considered hypotheses requiring further investigation rather than established mechanisms.

The toxicological relevance of several chemical classes detected in CB waste is independently established. Metals such as cadmium, lead, and chromium can produce multiple toxic effects depending on chemical form, dose, and exposure conditions [70]; several PAHs have recognized carcinogenic properties and environmental exposure to BTEX compounds has been associated with a range of adverse health outcomes [71]. Nevertheless, their occurrence in CB waste demonstrates a toxicologically relevant source of environmental contamination but does not, by itself, demonstrate adverse effects in environmentally exposed human populations.

Quantitative evidence frequently showed increasing biological responses with CB concentration and, in some studies, exposure duration. Acute and sublethal effects were observed in some animal models, including mortality or immobilization, oxidative stress, reproductive and developmental impairment, and behavioral changes. Dose–effect patterns, however, were not uniform: non-monotonic or biphasic responses, species-specific sensitivity, and modification by environmental conditions were also observed. Consequently, the available evidence does not support a single toxicity threshold applicable across CB waste; effects depend on concentration, duration, product characteristics, biological model, and environmental context.

Within the environmental health domain, CBs were shown to release contaminants at different rates and to remain environmentally relevant after ageing. PAHs were progressively released during prolonged water contact, whereas BTEX compounds showed more rapid release; nicotine, aromatic amines, phenolic compounds, metals, and particulate material also displayed condition-specific release patterns [25,38,40]. Long-term experiments documented progressive degradation and chemical transformation but persistence of cellulose acetate and measurable biological effects after extended ageing [58]. Field investigations further documented CB accumulation in urban, coastal, recreational, and stormwater environments [37,61,72]. Thus, the environmental relevance of CB waste extends beyond visible littering to chemical release, particulate pollution, persistence, and contaminant transport.

Within the animal health domain, effects across terrestrial, freshwater, and marine organisms included mortality, impaired growth and reproduction, developmental and behavioral alterations, oxidative stress, neurotoxicity, metabolic dysfunction, genotoxicity, histopathological changes, and bioaccumulation [41,47,56]. Smoked CBs or smoked-filter-derived materials frequently showed greater toxicity than corresponding unsmoked materials, although this pattern was not universal. Particle-associated effects were also reported: cellulose acetate microfibers increased toxicity and physically interfered with *Daphnia magna*, while filter-derived microplastics affected reproductive and molecular endpoints [48,60]. The diversity of affected taxa and endpoints supports the biological activity of CB-derived contaminants, although heterogeneity among experimental models limits extrapolation to population- or ecosystem-level effects.

Evidence within the plant health domain was less extensive but showed effects on germination, root and seedling development, biomass, chlorophyll-related parameters, mitotic activity, and chromosomal integrity. Responses varied by species and exposure level, ranging from inhibition at increasing concentrations to biphasic responses characterized by stimulation at lower exposures. Cytotoxic and genotoxic effects were also observed in *Allium cepa* [45]. These findings indicate that plant responses are heterogeneous and cannot be inferred directly from animal ecotoxicological evidence.

The limited comparisons between conventional CBs and waste from alternative tobacco or nicotine products suggest product-specific environmental effects. Piccardo et al. (2021) compared classic cigarette butts with materials described by the original authors as “electronic cigarette butts”; however, the specific electronic-cigarette product or device associated with these materials was not clearly identified, limiting the reproducibility and interpretation of this comparison [65]. Green et al. (2023) found distinct responses of *Lemna minor* to conventional CBs, disposable-electronic-cigarette waste, and residual e-liquid [43,65]. Although Green et al. clearly characterized the disposable e-cigarettes investigated, the disassembled-device and isolated-e-liquid treatments should be interpreted as experimentally modified scenarios useful for identifying potential sources of toxicity rather than as direct simulations of the most common post-consumption disposal condition. Interpretation of [29] et al. also requires caution, as the residues described as “e-cigarette butts” were in fact used HEETS tobacco sticks from a heated tobacco product, highlighting the importance of accurate product identification [29]. Because conventional cigarette butts and HEETS tobacco sticks derive from products with different designs, materials, and physicochemical processes, differences in metal content and leaching should be interpreted as product-specific comparisons rather than as evidence of a general difference between conventional cigarettes and electronic nicotine delivery systems. Environmental pollution from electronic cigarettes is also likely to vary according to device configuration. Disposable e-cigarettes may enter the waste stream as assembled units containing batteries, electronic circuitry, metallic and plastic components, wicks, and residual e-liquid, whereas refillable tank systems and pod- or cartridge-based devices generate different combinations of replaceable components and residual-liquid-containing waste. Consequently, the relative importance of electronic waste, metallic and plastic components, and direct e-liquid release may differ substantially among device types. These differences should be considered when designing environmentally realistic exposure studies and preclude extrapolation of findings from one e-cigarette configuration to another [49]. Regulatory frameworks, collection and recycling systems, and extended producer responsibility schemes may further influence the environmental fate of these products. Alternative filter materials also produced inconsistent findings: biodegradable cellulose filters did not necessarily reduce leachate toxicity in freshwater organisms, whereas life-cycle modelling suggested lower impacts for some biodegradable filter compositions [52,63]. The current comparative evidence is therefore limited not only by the small number of studies but also by inconsistent product terminology, incomplete characterization of some investigated residues, differences in product design and physicochemical characteristics, and limited environmental realism of some experimental exposure scenarios. Accordingly, the available evidence is insufficient to establish a consistent hierarchy of environmental hazard among conventional cigarettes, electronic cigarettes, heated tobacco products, and alternative filters.

The multi-domain evidence is particularly relevant to the One Health interpretation because these studies directly connected environmental processes with animal, plant, or human health-related outcomes. Human health was not represented as an exclusive domain; human-health-related evidence derived primarily from exposure or risk modelling rather than epidemiological or biomonitoring studies. Accordingly, the available evidence does not establish a causal relationship between environmental CB exposure and human disease. Instead, it identifies potential exposure pathways and modelled risks involving contaminants of established toxicological relevance. This interpretation is consistent with the OHHLEP definition of One Health, which recognizes the interdependence of human, animal, plant, and ecosystem health, and with the Quadripartite One Health Joint Plan of Action, which explicitly addresses the human–animal–plant–environment interface [19]. Importantly, the evidence in this review was unevenly distributed across these dimensions, with environmental and animal health more extensively investigated than plant or human health.

These findings also have implications for environmental policy and waste management. Prevention of littering, dedicated collection and disposal systems, consumer information, and extended producer responsibility have been proposed as complementary approaches to reduce the environmental burden associated with tobacco-product waste [73]. In the European Union, Directive (EU) 2019/904 explicitly includes tobacco products with filters and filters marketed for use with tobacco products within extended producer responsibility provisions [74]. Product-design interventions and alternative end-of-life management strategies may also reduce selected environmental impacts; in one life-cycle assessment, some alternative filter materials showed lower modelled impacts than conventional cellulose acetate filters [63]. However, biodegradability alone should not be assumed to result in lower ecotoxicity, since the experimental evidence included in this review did not consistently demonstrate reduced toxicity for biodegradable filter alternatives. Nevertheless, evidence on the real-world effectiveness of specific mitigation strategies remains limited, and laboratory or life-cycle modelling results should not be assumed to translate directly into environmental benefits.

To our knowledge, this is the first systematic review to integrate experimental toxicology, environmental monitoring and exposure assessment, risk assessment, and life-cycle evidence on CB and related tobacco product waste within an explicitly operationalized One Health framework. This should be distinguished from being the first systematic review of CB pollution, as previous systematic reviews have addressed the environmental hazards and toxicity of CB waste [3,13]. Additional strengths include the use of study-design-specific methodological appraisal and the distinction between an environmental matrix used experimentally and an outcome directly attributable to the environmental health domain.

Several limitations of the available evidence should be considered. Much of the evidence was generated under controlled laboratory conditions, while cigarette brands, smoking status, ageing, leachate preparation, concentrations, exposure duration, analytical methods, species, and endpoints varied substantially. This heterogeneity is particularly relevant because the chemical profile of cigarette waste may change according to smoking status and environmental ageing; for example, primary aromatic amine concentrations were substantially higher in freshly smoked cigarette butts than in unsmoked cigarettes and remained detectable, although generally reduced, in aged cigarette butts collected from urban environments [26]. Aquatic systems were more extensively investigated than terrestrial environments, whereas chronic low-dose exposure, repeated contamination, mixture toxicity, bioaccumulation, trophic transfer, and direct human exposure remain comparatively understudied. Moreover, some experimental concentrations had uncertain environmental representativeness, and incomplete chemical characterization of leachates sometimes limited the attribution of biological effects to individual constituents.

The review process also has limitations. Restriction to peer-reviewed articles published in English may have excluded relevant evidence. Moreover, substantial heterogeneity in study designs, exposure metrics, organisms, endpoints, and reporting units precluded quantitative meta-analysis and the derivation of a unified toxicity threshold. Consequently, the evidence was synthesized narratively, supported by structured comparison of quantitative dose–effect data where sufficiently comparable.

Furthermore, despite the inclusion of 47 studies overall, the evidence was unevenly distributed across study designs and One Health domains, resulting in relatively small numbers of studies within some specific methodological categories. This limits the extent to which domain- or design-specific patterns can be generalized. In particular, the methodological quality assessments should be regarded as structured descriptive evaluations of the individual studies rather than as analytical estimates of the certainty of the evidence within each domain. This limitation is especially relevant to the in vitro evidence, for which only seven studies were available for ToxRTool assessment.

Future studies should prioritize standardized CB-leachate preparation and reporting, environmentally realistic chronic exposures, comprehensive chemical and particulate characterization, and harmonized ecotoxicological endpoints. Greater attention is needed for terrestrial ecosystems, community-level effects, bioaccumulation and trophic transfer, and direct comparisons among conventional cigarettes, electronic cigarettes, heated tobacco products, and alternative filter materials. Studies involving emerging nicotine products should ensure accurate identification of the product category and characterization of the investigated devices or residues, clearly distinguishing electronic nicotine delivery systems from heated tobacco products, and should adopt exposure conditions that reflect realistic patterns of use, disposal, physical damage, residual liquid content, and environmental weathering. Finally, combining environmental monitoring with human exposure assessment or biomonitoring would help determine whether exposure pathways identified experimentally translate into measurable human exposure and would strengthen the assessment of tobacco product waste within a One Health framework.

## 5. Conclusions

This systematic review supports the recognition of cigarette butts and related tobacco product waste as an environmental issue extending beyond conventional litter pollution and requiring consideration within a One Health perspective. Their combined chemical and particulate nature, together with the interconnections identified across different health domains, highlights the need for an integrated approach to their environmental assessment and management.

However, the heterogeneity of the available evidence and the limited integration of environmental, biological, and human exposure data currently constrain a comprehensive assessment of their overall impact. In particular, evidence concerning emerging tobacco and nicotine products remains insufficient to determine whether their environmental profiles differ consistently from those of conventional cigarettes. Interpretation of the available comparative evidence is further limited by inconsistent product terminology, incomplete characterization of some investigated residues, and differences in experimental exposure scenarios.

Future research should move towards standardized and environmentally realistic approaches, with greater integration of environmental monitoring, ecotoxicology, and human exposure assessment. Studies of emerging products should clearly distinguish electronic nicotine delivery systems from heated tobacco products and accurately characterize the devices and residues investigated. Such evidence will be essential to support more robust One Health risk assessment and to inform prevention strategies, product design, waste management, and regulatory measures aimed at reducing the environmental burden of tobacco product waste.

## Figures and Tables

**Figure 1:**
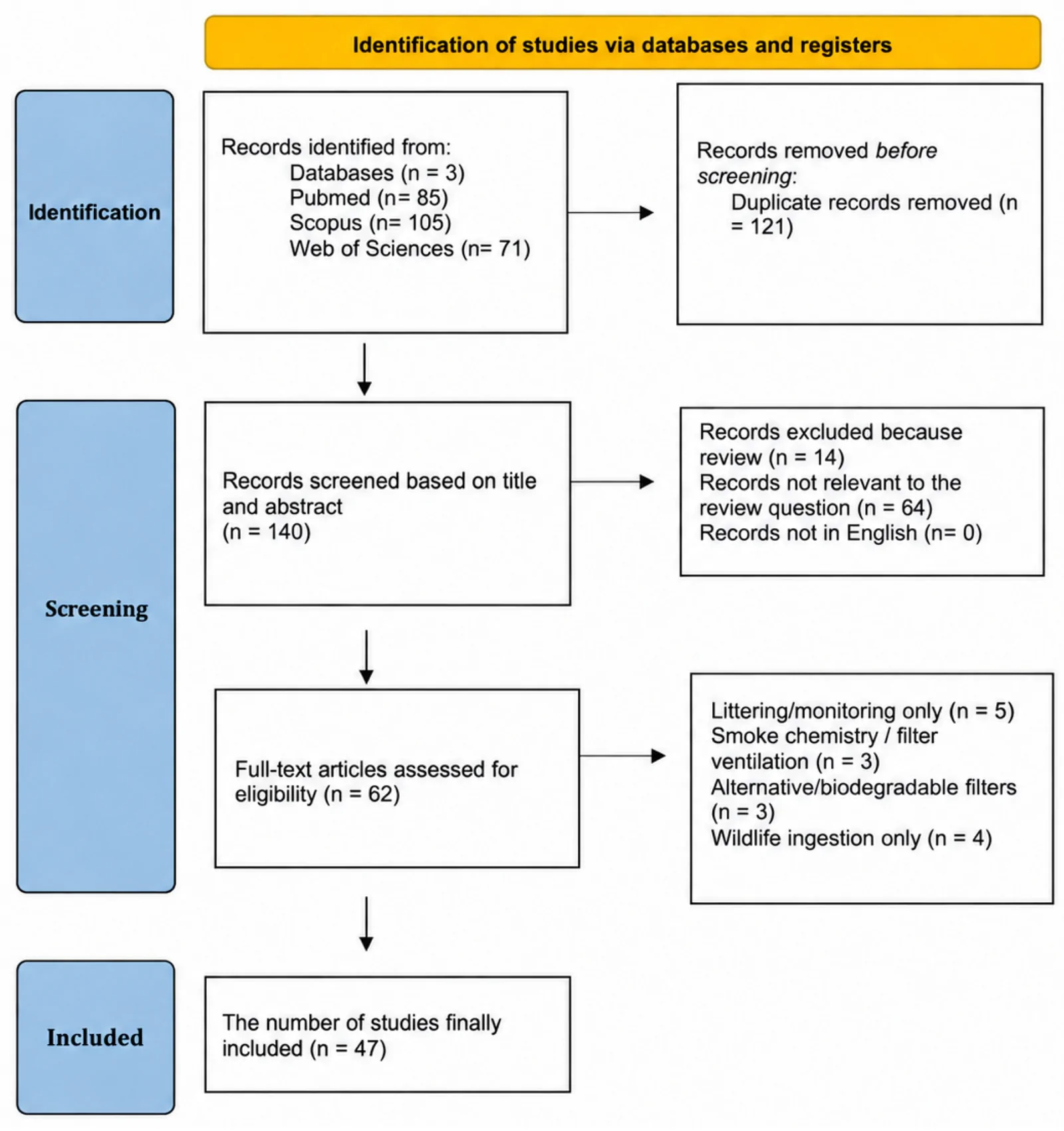
Flowchart of the study selection process according to the PRISMA guideline.

**Figure 2:**
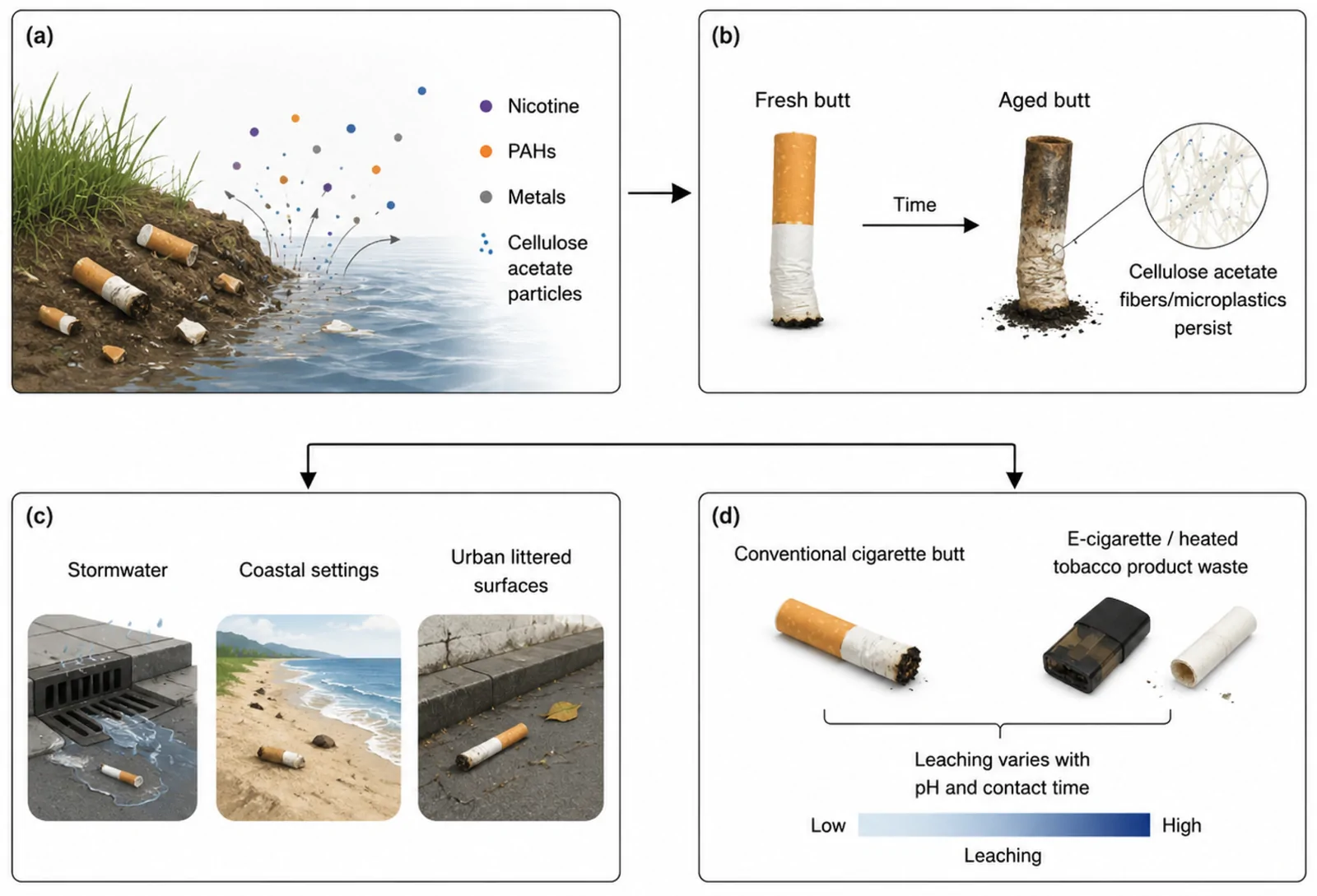
Environmental release, persistence, and transport of chemical contaminants and cellulose-acetate-derived particles from cigarette butts and related tobacco-product waste. (**a**) Release of nicotine, polycyclic aromatic hydrocarbons (PAHs), metals, and cellulose-acetate particles from discarded cigarette butts into the environment; (**b**) ageing of cigarette butts over time and persistence of cellulose-acetate fibers/microplastics; (**c**) occurrence and transport of cigarette-butt waste in stormwater, coastal settings, and urban littered surfaces; (**d**) product-specific differences in leaching from conventional cigarette butts and electronic-cigarette/heated-tobacco-product waste, influenced by pH and contact time.

**Figure 3:**
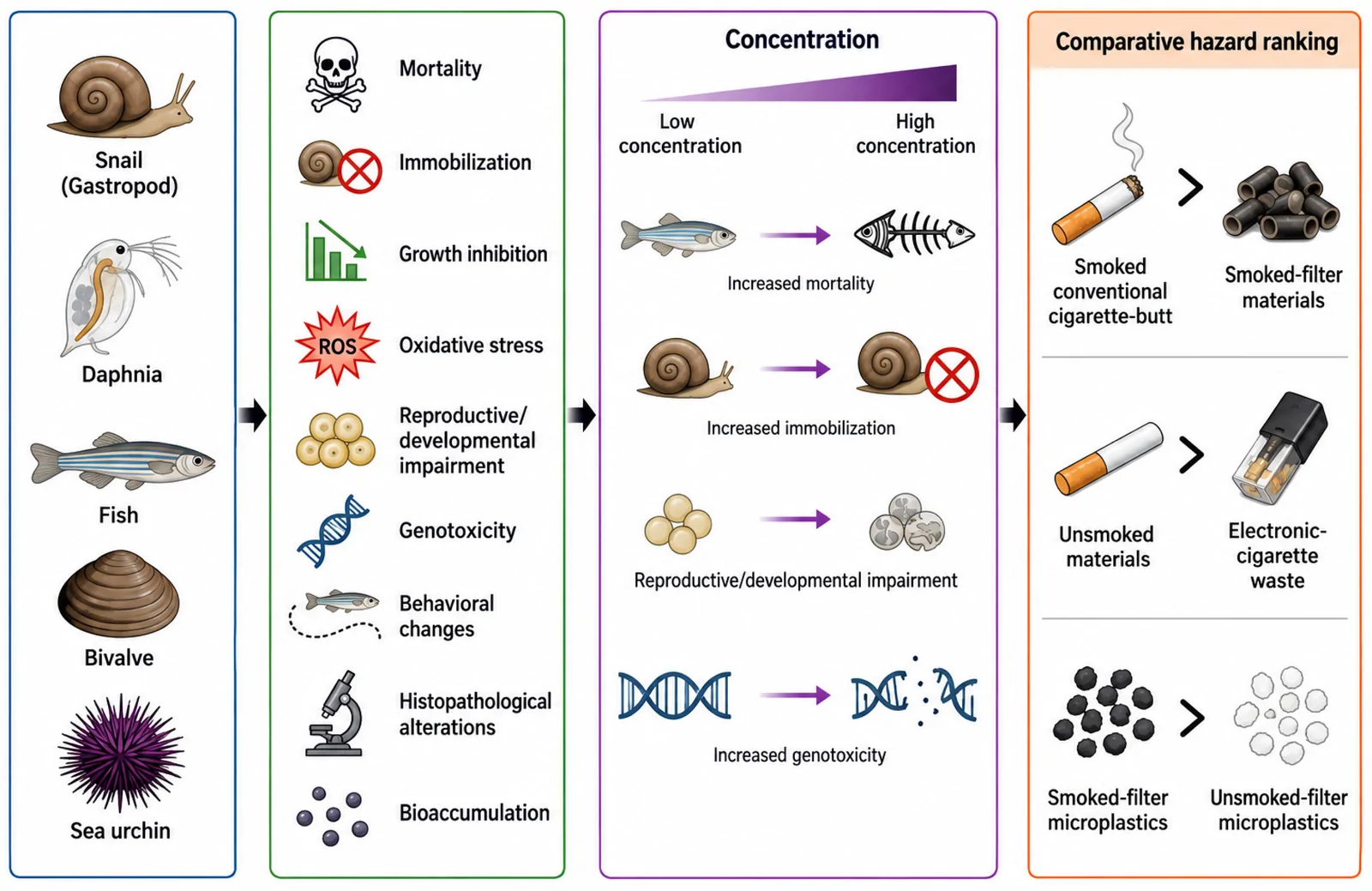
Main ecotoxicological effects of cigarette-butt and filter-derived waste across terrestrial, freshwater, and marine animal models, including concentration-dependent responses and product-related differences.

**Figure 4:**
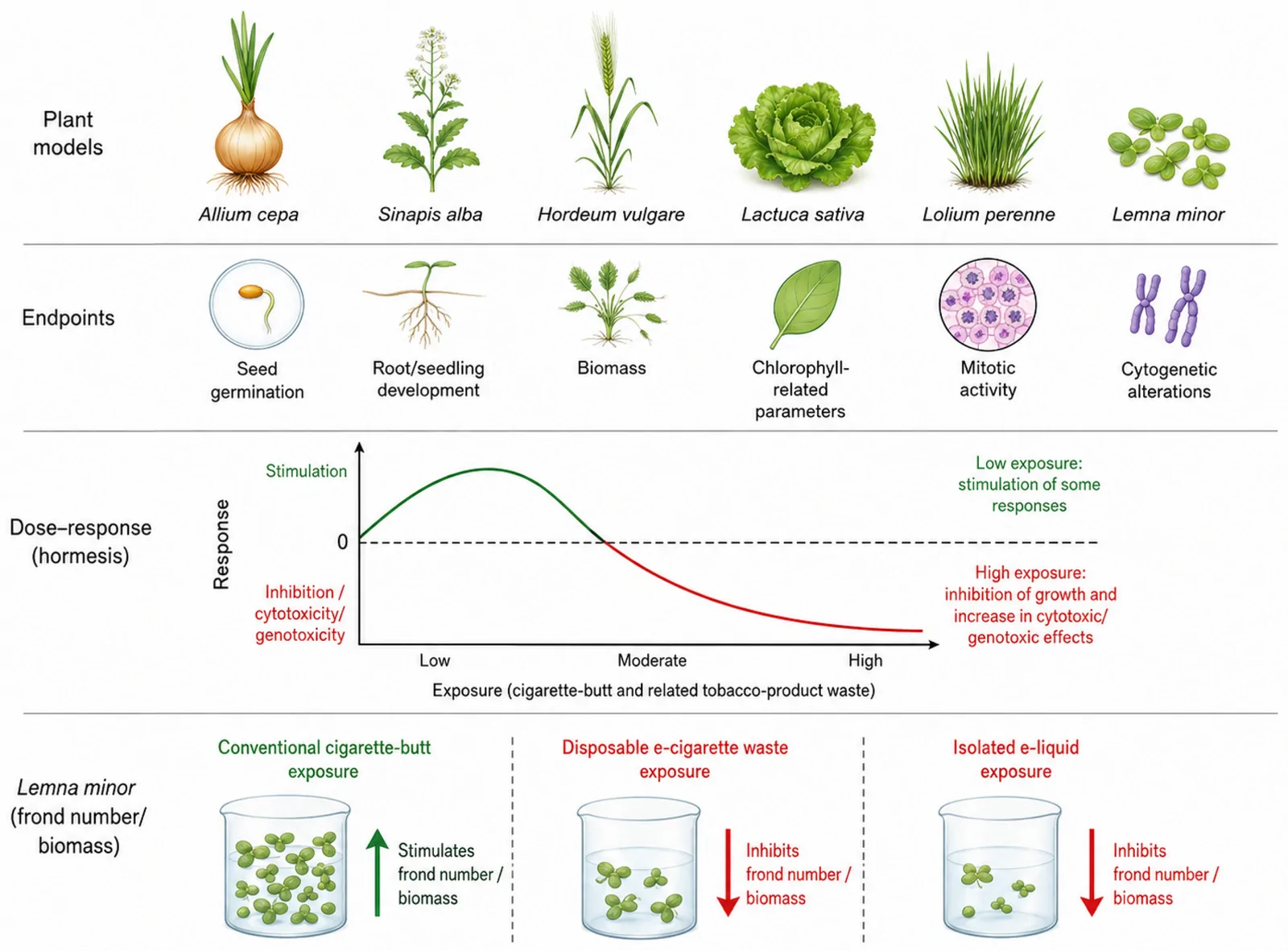
Plant responses to cigarette-butt and disposable-electronic-cigarette-related waste, highlighting species- and exposure-dependent effects and possible biphasic responses.

**Figure 5:**
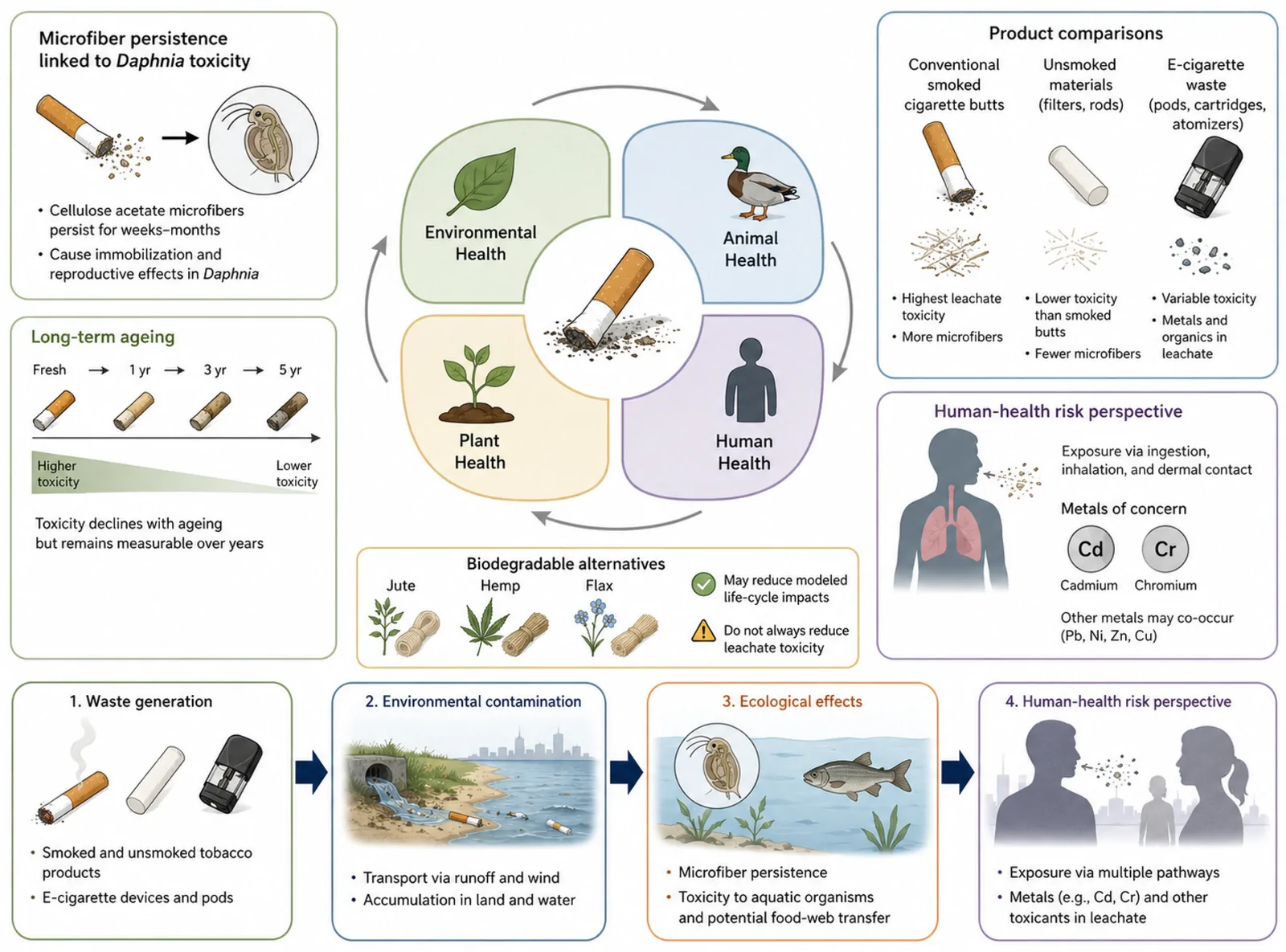
One Health integration of environmental contamination, ecological effects, product-specific differences, and modelled human-health risks associated with tobacco-product waste.

**Table 1:** Characteristics of the studies included in the systematic review within the Environmental health domain.

Author Year	Country	Study Design	Objective	Outcomes Assessed	Environmental Matrix/Experimental Medium *	Experimental Model Tobacco Product Investigated Exposure Conditions
Arfaeinia et al. 2024 [22]	Iran	Laboratory experimental study	To determine the release of PCBs ** and OCPs ** from cigarette butt leachates, compare pollutant release among different cigarette butt types, and evaluate their potential ecological risk.	PCBs ** and OCPs ** Toxic equivalency Risk quotient	Natural seawater (leaching medium)	Experimental model: laboratory leaching experiment Tobacco product investigated: Smoked cigarette butts, Smoked filters, Unsmoked filters Exposure conditions: SCBs: 2 CB/L; SFs: 4 filters/L; USFs: 8 filters/L in natural seawater for 24 h; leachates filtered before chemical analysis.
Caridi et al. 2020 [23]	Italy	In vivo laboratory experimental study	To assess the acute toxicity of smoked cigarette-butt leachate on three cultured genera of benthic foraminifera and investigate the associated morphological and biochemical alterations.	Nicotine concentration, LC_50_, survival, shell decalcification, cytoplasmic alterations and FTIR biochemical profile.	Artificial seawater (leaching/exposure medium)	Experimental model: Laboratory ecotoxicological bioassay Tobacco product investigated Smoked conventional cigarette butts Exposure conditions Ecotoxicity: stock solution of 32 smoked CBs/L in artificial seawater; test concentrations: 1, 2, 4, 8 and 16 CBs/L (1.96, 3.91, 7.82, 15.63 and 31.25 mg nicotine/L). Three benthic foraminiferal taxa were exposed for 48 h (4 replicates × 5 individuals), followed by CellTracker Green staining and a 72-h recovery period.
Dobaradaran et al. 2020 [24]	Germany	Laboratory experimental study	To quantify the leaching of EPA priority PAHs from smoked cigarette butts into different water matrices under controlled laboratory conditions and evaluate their potential environmental relevance.	PAH concentrations, total PAH (ΣPAH) concentrations, individual PAH profiles and comparison with Water Framework Directive environmental quality standards.	Deionized water, tap water, and river water (leaching media)	Experimental model: laboratory leaching experiment Tobacco product investigated: smoked conventional cigarette butts Exposure conditions Smoked cigarette butts were immersed in deionized water, tap water and river water for 4 h, 1 day, 2 days, 7 days and 21 days. Additional tap- and river-water experiments were performed with sodium azide to inhibit microbial degradation.
Dobaradaran et al. 2021 [25]	Germany	Laboratory experimental study	To investigate the leaching behaviour of BTEX compounds from smoked cigarette butts into different water matrices and evaluate the influence of contact time and post-smoking interval on BTEX release.	BTEX concentrations, individual BTEX profiles, partitioning behaviour (log KOC/water and log Kwater/air) and effect of contact time and post-smoking interval on BTEX release.	Deionized water (leaching medium); river water (sampled/leaching medium)	Experimental model: laboratory leaching experiment Tobacco product investigated: smoked conventional cigarette butts Exposure conditions Freshly smoked cigarette butts (0.28–0.37 g) were immersed in 10 mL of distilled water or river water. Leaching experiments were performed for 15 min, 45 min, 1.5 h, 3 h, 6 h and 24 h at room temperature without agitation. Additional experiments evaluated the effect of delaying immersion after smoking (0, 15, 45 min, 1.5 h and 3 h).
Dobaradaran et al. 2022 [26]	Germany	Laboratory experimental study	To quantify and compare the concentrations of primary aromatic amines in unsmoked cigarettes, freshly smoked cigarette butts and aged cigarette butts collected from urban environments.	Individual aromatic amine concentrations, total aromatic amine concentrations and comparison among unsmoked cigarettes, freshly smoked cigarette butts and aged cigarette butts.	Urban streets (sampling setting); cigarette-butt material (analytical matrix)	Experimental model: laboratory chemical characterization Tobacco product investigated: unsmoked conventional cigarettes, freshly smoked conventional cigarette butts and aged conventional cigarette butts. Exposure conditions Ten primary aromatic amines were quantified in three sample categories: unsmoked cigarettes, freshly smoked cigarette butts and aged cigarette butts collected from urban streets. Samples were analysed by LC–MS/MS without experimental leaching or biological exposure.
Dobaradaran et al. 2023 [27]	Germany	Laboratory experimental study	To investigate the leaching of aromatic amines from smoked cigarette butts into different water matrices, evaluate their partitioning behaviour and assess their potential ecological risk to aquatic organisms.	Individual aromatic amine concentrations, total aromatic amine concentrations, partition coefficients (log KOC/water and log Kwater/air) and ecological risk assessment.	Distilled water (leaching medium); river water (sampled/leaching medium)	Experimental model Laboratory leaching experiment Tobacco product investigated Smoked conventional cigarette butts Exposure conditions Three freshly smoked cigarette butts (mean weight 1.06 ± 0.11 g) were immersed in 50 mL of distilled water or river water. Leaching experiments were performed for 15 min, 1.5 h, 1, 2, 5, 15 and 30 days under static laboratory conditions at room temperature.
Dobaradaran et al. 2024a [28]	Germany	Laboratory experimental study	To quantify nicotine leaching from different cigarette butt components (freshly smoked cigarette butts, aged cigarette butts, remaining tobacco plus ash, and filter plus paper) into water over time and evaluate their ecological risk.	Nicotine concentrations Nicotine leaching kinetics Nicotine release from different cigarette butt components Ecological risk was estimated using published toxicity data for aquatic organisms.	Deionized water (leaching medium)	Experimental model: Laboratory leaching experiment Tobacco product investigated Freshly smoked conventional cigarette butts Aged conventional cigarette butts Remaining tobacco plus ash from freshly smoked cigarette butts Filter plus paper from freshly smoked cigarette butts Exposure conditions Freshly smoked cigarette butts, aged cigarette butts collected from streets, remaining tobacco plus ash, and filter plus paper were immersed separately in deionized water. Leaching experiments were performed from 1 min to 30 days.
Dobaradaran et al. 2024b [29]	Germany	Laboratory experimental study	To quantify the release of phenolic compounds from different cigarette butt components into water over time and evaluate their ecological risk for aquatic organisms.	Individual phenolic compound concentrations Total phenolic compound release Leaching kinetics Ecological risk assessment	Ultrapure water (leaching medium)	Experimental model: Laboratory leaching experiment Tobacco product investigated Freshly smoked conventional cigarette butts Aged conventional cigarette butts Filter plus paper from freshly smoked cigarette butts Remaining tobacco plus ash from freshly smoked cigarette butts Exposure conditions Freshly smoked cigarette butts, aged cigarette butts, filter plus paper, and remaining tobacco plus ash were immersed separately in ultrapure water (10 cigarette butts/L) in 2-L glass containers. Leachates were collected after 1, 5, 15 and 30 min; 1 and 2 h; and 1, 7, 21 and 30 days without agitation at room temperature.
dos Santos et al. 2024 [30]	Brazil	In vivo laboratory experimentalstudy.	To investigate the effects of increasing concentrations of cigarette butt leachate on chlorophyll-a concentration, cell density, species richness, and community composition of a natural freshwater phytoplankton community.	Chlorophyll-a concentration; total phytoplankton cell density; species richness; taxonomic/community composition; species dominance and abundance distribution. Community composition was evaluated using presence/absence and density data.	Natural eutrophic lake water (sampled/leaching/exposure medium)	Experimental model: Natural freshwater phytoplankton community maintained in laboratory microcosms. Tobacco product investigated: Artificially smoked conventional cigarette butts were used to prepare the leachate. Cigarettes were artificially smoked to 2 cm above the filter and the ash was discarded. Exposure conditions A stock leachate was prepared with 50 smoked CBs L^−1^ in filtered natural lake water and left for 24 h at 15–18 °C. Phytoplankton microcosms were then exposed for 96 h to 0 (control), 1, 5, or 10 CBs L^−1^, with 5 replicates per treatment (20 experimental units), at 28 °C, 12:12 h light/dark cycle; microcosms were manually agitated for 30 s every 24 h.
El Hadri et al. 2021 [31]	France	Laboratory experimental study	To investigate the sorption capacity of nanoplastics on cigarette filters and evaluate their potential co-transport with cigarette butts in the environment, with particular attention to the effects of nanoplastic size and morphology on sorption behaviour	Nanoplastic residual fraction; sorption kinetics and equilibrium time; cigarette butt–water partition coefficient (KSW); water sampling rate (Rs); effects of nanoplastic size and morphology and filter characteristics on sorption; and modelling of the physicochemical mechanisms governing nanoplastic retention.	Freshwater (sorption medium)	Experimental model: Laboratory sorption experiment. Tobacco product investigated: Unused cellulose acetate cigarette filters, Exposure conditions: One prehydrated cigarette filter was placed in 10 mL of freshwater containing 5 mg L^−1^ nanoplastics and gently agitated at 100 rpm and 25 ± 1 °C. Experiments were conducted in triplicate. Three sizes of spherical PSLs (50, 180 and 500 nm) and fragmented NPTf were tested with both filter types, and sorption was monitored over time. A qualitative experiment additionally exposed unused and used cigarette butts to 20 mg L^−1^ blue 200-nm PSLs under similar exposure conditions.
Garshasbi et al. 2026 [32]	Iran	Field observational study	To quantitatively assess and map the spatial distribution of macroplastics and cigarette butts in Bushehr’s stormwater collection network, characterize macroplastics according to abundance, color, type, size and source, and assess pollution severity using CEI and CBPI.	Macroplastic abundance and spatial distribution; macroplastic item type, polymer type, source, size, color and material form; cigarette butt abundance and density; spatial differences among sampling stations; CEI; and CBPI.	Urban stormwater collection network (sampling setting)	Experimental model: Field survey. Tobacco product investigated: Discarded cigarette butts. Exposure conditions: Not applicable.
Guttmann et al. 2024 [33]	Germany	In vitro laboratory experimental study	To investigate whether CB leachate alters the dynamics of a cyanobacterium–chytrid fungus host–parasite system by evaluating its effects on cyanobacterial growth and on the prevalence and intensity of chytrid infection.	Cyanobacterial biovolume/growth; chytrid infection prevalence; chytrid infection intensity; concentrations of macroelements, trace elements, nicotine, and cotinine in CB leachate.	Distilled sterile water (leaching medium); Z8 freshwater culture medium (exposure medium)	Experimental model: Laboratory leaching experiment; laboratory ecotoxicological bioassay. Tobacco product investigated: Smoked self-rolled cigarette butts with cellulose acetate filters. Exposure conditions: The stock leachate was prepared by soaking smoked CBs in distilled sterile water for 24 h at approximately 18 °C in darkness with gentle agitation, followed by sequential filtration; the final stock concentration was 100 CBs/L. In the bioassay, infected and uninfected *P. agardhii* cultures were exposed to leachate corresponding to 0.2, 2 and 10 CBs/L, with an unexposed control. The experiment comprised 40 experimental units: 2 conditions × 3 concentrations plus control × 5 replicates, with a final volume of 30 mL per unit. Cultures were maintained at 16 °C under continuous light (20 µmol photons m^−2^ s^−1^) for 7 days, with sampling on days 0, 1, 3, 5 and 7.
Kouhi et al. 2025 [34]	Iran	Field observational study	To compare two sampling approaches for estimating discarded CB abundance on recreational beaches in Mazandaran Province and to estimate the potential environmental dispersion of heavy metals associated with this cigarette waste along the Caspian Sea coast.	CB abundance and density (CBs/m^2^); comparison among sampling methods; wet and dry CB weight and moisture; pooled concentrations of 14 metals in discarded CBs; and estimated metal emissions per unit beach area (μg/m^2^) according to sampling method.	Recreational beach surface and sand (sampling setting)	Experimental model: Field survey. Tobacco product investigated: Discarded smoked cigarette butts Exposure conditions: Not applicable.
Martínez-Ruiz et al. 2026 [35]	Germany	In vitro laboratory experimental study	To investigate the metabolic responses of the toxigenic cyanobacterium Planktothrix agardhii to simultaneous abiotic and biotic stressors, specifically MET or CB leachate in the presence or absence of the chytrid parasite Rhizophydium megarrhizum, using a multi-biomarker approach and IBRv2.	Protein and lipid content; oxidative stress biomarkers (TBARS, SOD, GST); total, intracellular and extracellular microcystins; IBRv2; cyanobacterial biovolume; chytrid infection prevalence and intensity; and chemical concentrations/stability of MET, nicotine and cotinine.	Sterile distilled water (leaching medium); Z8 freshwater culture medium (exposure medium)	Experimental model: Laboratory leaching experiment; laboratory ecotoxicological bioassay. Tobacco product investigated: Leachate from smoked self-rolled cigarette butts. Exposure conditions: CB leachate stock (100 CBs/L) was prepared by soaking self-rolled CBs in sterile distilled water in the dark under gentle agitation for 24 h, followed by sequential filtration. The exposure experiment used a 2 × 3 factorial design comprising infected and uninfected *P. agardhii* and three treatments: control, 100 μg/L MET, and CB leachate equivalent to 1 CBs/L. Five replicates were used per treatment (30 experimental units); cultures were exposed for 5 daysat 16 °C under continuous light.
Michael et al. 2022 [36]	India	Laboratory experimental study	To determine the concentrations of Cd, Cu, Fe, Pb, Sn, Zn, and Hg in different brands of unsmoked and smoked cigarettes and cigarette butts, and to evaluate the amounts of these metals released into an aqueous medium after leaching.	Concentrations of Cd, Cu, Fe, Pb, Sn, Zn, and Hg in the original cigarette materials and in their 24-h aqueous leachates; differences among smoked versus unsmoked materials, cigarette versus butt portions, and the three commercial brands.	Aqueous solution (leaching medium)	Experimental model: Laboratory leaching experiment. Tobacco product investigated: Unsmoked cigarette, smoked cigarette, unsmoked cigarette butt, and smoked cigarette butt from three commercial cigarette brands, classified by the authors as expensive, moderate, and cheap. Exposure conditions: Separate leachates of USC, SC, USB, and SB from each of the three brands were prepared by immersion in an aqueous solution for 24 h, with five replicates per sample and a blank without cigarette material.
Mohammadi et al. 2025 [37]	Iran	Laboratory experimental study	To determine the levels and characteristics of microfibers released from cigarette filters into different water matrices at different exposure times; compare microfiber release according to cigarette brand, water type, environmental condition, and exposure duration; assess pollution levels and potential ecological risk using PLI and RQ; and estimate the global emission of cigarette-filter-derived microfibers into aquatic environments.	Number of released microfibers; microfiber size, color, polymer composition, surface morphology and elemental composition; effects of exposure time, water type, cigarette brand, and laboratory vs outdoor conditions on microfiber release; PLI; RQ; and estimated global microfiber emission into aquatic environments.	Distilled water and natural seawater (leaching media)	Experimental model: Laboratory leaching experiment. Tobacco product investigated: Cigarette filters from two conventional cigarette brands. Exposure conditions: Cigarette filters were exposed to 100 mL of distilled water or seawater under two environmental conditions: laboratory environment without sunlight exposure and outdoor environment with sunlight exposure. Six exposure durations were investigated: 1, 2, 7, 15, 30, and 60 days. Three replicates were performed for each exposure time, with three filters per run.
Tan et al. 2026 [38]	China	Laboratory experimental study	To comprehensively characterize the hazardous properties of discarded cigarette butts according to Chinese hazardous-waste identification standards by assessing leaching toxicity, toxic substance content, and estimated acute toxicity, and to determine whether DCBs meet the criteria for classification as hazardous waste.	Leaching concentrations of hazardous substances; toxic substance content in DCBs; cumulative equivalent toxicant content relative to hazardous-waste thresholds; contribution of individual contaminants to cumulative toxicity; estimated acute toxicity); and hazardous-waste classification according to Chinese regulatory criteria.	Acidic extraction solution and deionized water (leaching media)	Experimental model: Laboratory leaching experiment. Tobacco product investigated: Discarded smoked conventional cigarette butts of mixed brands. Exposure conditions: For leaching-toxicity assessment, pollutant-specific extraction procedures were used according to Chinese hazardous-waste standards. An H_2_SO_4_:HNO_3_ acid mixture (2:1; pH 3.20 ± 0.05) was used for metals and semi-volatile organic compounds, whereas deionized water at a liquid-to-solid ratio of 10:1 was used for cyanide and volatile organic compounds. Extractions lasted 18 h. Toxic substance content was determined after contaminant-specific sample preparation, and acute toxicity was estimated computationally using measured toxicant concentrations and reference LD50/LC50 data under a worst-case assumption.
Venugopal et al. 2021 [39]	United States	Combined field and laboratory study	To characterize and quantify the chemical composition of leachate from discarded CBs, compile CB- and cigarette-filter-derived leachate chemicals reported in the literature, and characterize the acute and chronic aquatic ecological hazards of the identified chemicals using GHS classifications and HC values derived from SSDs (Species Sensitivity Distributions)	Chemical constituents and concentrations in CB leachate; acute and chronic aquatic toxicity classification of identified chemicals; HC50 values for acute and chronic aquatic toxicity; identification of chemicals included in the FDA HPHC list or proposed additions.	Deionized water (leaching medium)	Experimental model: Field survey, Laboratory leaching experiment, ecological hazard assessment Tobacco product investigated: Newly discarded smoked cigarette butts collected from designated smoking areas. Exposure conditions Newly discarded CBs were collected from three designated smoking locations after a 4-h accumulation period. For non-volatile chemical analysis, CBs were extracted in deionized water at a 20:1 water-volume-to-CB ratio and tumbled end-over-end for 18 h at 30 rpm, followed by filtration through a 45-μm membrane. Volatile constituents were extracted under sealed, pressurized, zero-headspace conditions. Thirty CBs were used for volatile analysis and 79 for the remaining targeted constituents. No organisms were experimentally exposed; aquatic hazard was characterized using GHS classifications and literature-derived HC50 values based on SSDs.
Yang et al. 2023 [40]	China	Combined field and laboratory study	To quantify cigarette butt littering in different urban and coastal environments in Dalian, China, and to evaluate the release of heavy metals, PAHs, microplastics, and other particles from cigarette butts of different brands.	CB abundance and density across sampling sites and between workdays and weekends; cigarette brand distribution; heavy metal concentrations in CB leachates; PAH concentrations in CB leachates; presence and morphology of microplastics and nanoscale particles; differences in pollutant release between the highest- and lowest-abundance cigarette brands; estimated annual plastic waste generated from discarded CBs in China.	Urban and coastal public surfaces (sampling setting); deionized water (leaching medium)	Experimental model: Field survey; laboratory leaching experiment. Tobacco product investigated: Littered smoked conventional cigarette butts from multiple brands; machine-smoked conventional cigarettes from the highest- and lowest-abundance brands identified during the field survey. Exposure conditions: Field sampling was conducted in June 2023 along randomly selected routes at Blackrock Reef beach, Xinghai Park, and Shuangxing City vegetable market, on different days of the week between 4:00 PM and 11:00 PM following morning cleaning activities. For laboratory leaching, cigarettes from the highest- and lowest-abundance brands were machine-smoked under identical conditions; 20 CBs from each brand were placed in 1 L of deionized water and gently stirred for 12 h to simulate disturbance in an aquatic environment.
Yang et al. 2024 [41]	China	Combined field and laboratory study	To quantify cigarette butt littering across coastal and inland Chinese cities and different land-use types, estimate the degree of cigarette butt pollution using the Cigarette Butts Pollution Index, and characterize microplastics and heavy metals released from cigarette butts under simulated aqueous environmental conditions.	Plastic litter abundance and composition; CB density across cities and land-use types; CBPI and pollution classification; release and morphology of microplastics; heavy metals associated with released particles; concentrations of heavy metals in CB leachate; and estimated daily heavy-metal release from littered CBs in Chinese cities.	Urban and coastal public surfaces (sampling setting); deionized water (leaching medium)	Experimental model: Field survey; laboratory leaching experiment. Tobacco product investigated: Littered smoked conventional cigarette butts collected from urban and coastal environments. Exposure conditions: Field sampling was conducted from June to August 2023 at 12 sites across Guangzhou, Dalian, Baoding, and Meizhou, representing beaches, parks, markets, and culture squares. Each sidewalk was sampled continuously for 5–7 days, with daily collection between 4:00 PM and 11:00 PM. For the laboratory experiment, 20 randomly selected littered CBs were immersed in 1 L of deionized water and gently stirred for 12 h to simulate disturbance in an aquatic environment. The leachate was filtered for microplastic characterization and heavy-metal analysis, with procedural and reagent blanks included.
Yona et al. 2025 [42]	Indonesia	Combined field and laboratory study	To assess the distribution and characteristics of cigarette butts across tourist beaches, investigate the seawater leaching patterns of representative heavy metals (Fe, Cd, Pb, and Zn) according to cigarette-butt degradation state and immersion duration, and estimate the potential contribution of cigarette butts to heavy metal pollution at four beaches in Prigi Bay, East Java.	CB abundance, spatial distribution, size, weight, and degradation state; solid-phase concentrations of Fe, Cd, Pb, and Zn; heavy metal concentrations released into seawater after 1, 7, and 30 days; effects of immersion duration and cigarette-butt degradation state on metal release; and estimated heavy metal pollution at individual beaches.	Beach environment (sampling setting); commercially available seawater (leaching medium)	Experimental model: Field survey; laboratory leaching experiment. Tobacco product investigated: Field-collected whole and decayed smoked cigarette butts; freshly smoked conventional cigarette butts used as a comparison group. Exposure conditions: Field sampling was conducted from March to April 2024 at Cengkrong, Prigi, Karanggongso, and Mutiara beaches using randomly positioned 5 × 5 m transects in foreshore and backshore zones; 31 transects were surveyed three times per week. For laboratory leaching, approximately 2.0 ± 0.03 g of FCBs, WCBs, or DCBs were immersed separately in 100 mL commercially available seawater in 125-mL HDPE vials for 1, 7, or 30 days (*n* = 3) at approximately 25 °C, salinity 35.7 ppt, and pH 8.2, with manual shaking during exposure.

Note. Table 1, Table 2 and Table 3 include only studies classified as addressing a single One Health domain. Studies addressing two or more One Health domains were classified as multi-domain and are reported exclusively in Table 4; therefore, no study is duplicated across Table 1, Table 2, Table 3 and Table 4. * Environmental matrix/experimental medium indicates, as applicable, the environmental matrix directly sampled or experimentally used, the field sampling setting, or the medium used for leaching, exposure, degradation, or other experimental procedures. The specific function is reported in parentheses. ** **PCBs:** PCB18, PCB28, PCB44, PCB52, PCB77, PCB81, PCB101, PCB105, PCB114, PCB118, PCB123, PCB126, PCB128, PCB138, PCB149, PCB153, PCB156, PCB157, PCB167, PCB169, PCB170, PCB180, PCB189, PCB194. **OCPs:** α-HCH, β-HCH, γ-HCH, δ-HCH, heptachlor, aldrin, heptachlor epoxide, endosulfan I, 4,4′-DDE, dieldrin, endrin, 4,4′-DDD, endosulfan II, 4,4′-DDT, methoxychlor, endrin aldehyde, endosulfan sulfate. **Abbreviations:** BTEX, benzene, toluene, ethylbenzene and xylenes; CB, cigarette butt; CBPI, Cigarette Butt Pollution Index; CEI, Clean Environment Index; DCB, decayed cigarette butt; EPA, Environmental Protection Agency; FCB, freshly smoked cigarette butt; FTIR, Fourier-transform infrared spectroscopy; GHS, Globally Harmonized System of Classification and Labelling of Chemicals; HC50, hazardous concentration for 50% of species; HPHC, harmful and potentially harmful constituents; IBRv2, Integrated Biomarker Response version 2; KOC/water, organic carbon–water partition coefficient; Kwater/air, water–air partition coefficient; KSW, cigarette butt–water partition coefficient; LC50, median lethal concentration; LC–MS/MS, liquid chromatography–tandem mass spectrometry; LD50, median lethal dose; MET, metolachlor; NPTf, fragmented polystyrene nanoplastics; OCPs, organochlorine pesticides; PAHs, polycyclic aromatic hydrocarbons; PCBs, polychlorinated biphenyls; PLI, Pollution Load Index; PSLs, polystyrene latex nanoplastics; RQ, risk quotient; Rs, water sampling rate; SB, smoked cigarette butt; SC, smoked cigarette; SSDs, Species Sensitivity Distributions; TEQ, toxic equivalency; USB, unsmoked cigarette butt; USC, unsmoked cigarette; WCB, whole cigarette butt; WFD, Water Framework Directive; ΣPAH, sum of polycyclic aromatic hydrocarbons.

**Table 2:** Characteristics of the studies included in the systematic review addressing the Plant health domain.

Author Year	Country	Study Design	Objective	Outcomes Assessed	Environmental Matrix/Experimental Medium *	Experimental Model Tobacco Product Investigated Exposure Conditions
Green et al. 2023 [43]	United Kingdom	In vivo laboratory experimental study	To assess the effects of smoked cigarette butts, used disposable e-cigarettes, and e-liquid extracted from e-cigarettes on the growth and chlorophyll content of the freshwater macrophyte Lemna minor, and to compare the effects of these different smoking-related waste products.	L. minor biomass, root length, frond number, chlorophyll-a, chlorophyll-b, and total chlorophyll content. Frond number was assessed at baseline and after 7 and 14 days; biomass, root length and chlorophyll were assessed after 14 days.	Artificial freshwater (exposure medium)	Experimental model: Whole- product and e-liquid exposure bioassay.. Tobacco product investigated: smoked conventional cigarette butts; used disposable e-cigarettes (intact/closed and experimentally opened/disassembled); and e-liquid extracted from disposable e-cigarettes. Exposure conditions: Five experimental groups were established, each with 10 replicate microcosms (N = 50): control; 1 smoked cigarette butt/L; 1 closed used e-cigarette/L; 1 opened/disassembled used e-cigarette/L; and 1 mL e-liquid/L (0.1% *v*/*v*). Each 1-L microcosm initially contained 15 L. minor plants. Exposure lasted 14 days at 18 °C under a 16:8 h light/dark cycle. The e-liquid concentration was selected because naturally used e-cigarettes of the same brand retained approximately 1.04 ± 0.06 mL residual e-liquid.
Jakimiuk et al. 2022 [44]	Poland/Czech Republic	In vitro laboratory experimental study	To assess the toxicity of smoked cigarette-butt leachates at different concentrations and leaching times on seed germination and early plant development of Sinapis alba and Hordeum vulgare, and to evaluate the effects of plant species, CB concentration, and their interaction on plant growth.	Seed germination (SG); relative seed germination (RSG); relative radicle growth (RRG); germination index (GI); and root length, evaluated according to CB concentration, leaching time and plant species.	Tap water (leaching medium)	Experimental model: laboratory leaching experiment; laboratory ecotoxicological bioassay. Tobacco product investigated: smoked conventional filtered cigarette butts. Exposure conditions: leachates were prepared in 100 mL water using 3, 5, or 10 smoked CBs, corresponding to 30, 50 and 100 CB/L, and were allowed to leach for 24 h, 7 days, or 21 days without mixing. After each leaching period, CBs were removed and 5 mL of each leachate was applied to 9-cm Petri dishes containing 10 seeds of either *S. alba* or *H. vulgare*. Each treatment and control was performed in triplicate. Seeds were incubated for 72 h in darkness at 20 ± 2 °C before assessment.
Sadıç et al. 2025 [45]	Türkiye	In vivo laboratory experimental study	To evaluate the physiological, cytotoxic, and genotoxic effects of leachates from smoked light cigarette butts and unsmoked light cigarette filters on *Allium cepa* root tips and to investigate concentration-related toxic effects on plant growth and cellular/genetic endpoints.	Leachate pH; root length; root number; GI; RGI; root morphology; MI; number of dividing cells; chromosomal and nuclear abnormalities, including fragments, anaphase/chromatid bridges, chromosome stickiness, polar deviations/misalignment, and micronuclei; and total chromosomal damage.	Chlorine-free drinking water (leaching/exposure medium) and 0.02 M EMS as the positive control.	Experimental model: laboratory leaching experiment; laboratory ecotoxicological bioassay. Tobacco product investigated: smoked light cigarette butts and unsmoked light cigarette filters from four cigarette brands. Exposure conditions: smoked light cigarette butts and unsmoked filters were placed separately in 1 L of chlorine-free drinking water and leached for 24 h. Stock solutions were diluted to 6.25%, 12.5%, 25%, 50%, and 100% *v*/*v*. *A. cepa* bulbs were exposed for 5 days, with endpoints assessed on day 6.

*Note*. CB, cigarette butt. * Environmental matrix/experimental medium refers, as applicable, to the environmental matrix directly sampled or experimentally used, the field sampling setting, or the medium used for leaching, exposure, degradation, or other experimental procedures; its specific function is indicated in parentheses.Table 1, Table 2 and Table 3 include only studies classified as addressing a single One Health domain. Studies addressing two or more One Health domains were classified as multi-domain and are reported exclusively in Table 4; therefore, no study is duplicated across Table 1, Table 2, Table 3 and Table 4.

**Table 3:** Characteristics of the studies included in the systematic review addressing the Animal health domain.

Author Year	Country	Study Design	Objective	Outcomes Assessed	Environmental Matrix/Experimental Medium *	Experimental Model Tobacco Product Investigated Exposure Conditions
Bakr et al. 2025 [46]	Egypt	In vivo laboratory experimental study	To evaluate the ecotoxicological effects of cellulose acetate nanoplastics derived from smoked and unsmoked cigarette butts on the earthworm Allolobophora caliginosa and compare their toxicity with commercially available cellulose acetate nanoplastics.	Mortality, growth inhibition, nanoplastic uptake, oxidative stress biomarkers, antioxidant enzyme activity, detoxification enzyme activity, DNA damage (Comet assay)	Artificial soil (exposure medium)	Experimental model: laboratory ecotoxicological bioassay Tobacco product investigated: smoked cigarette butts, unsmoked cigarette butts Exposure conditions: adult Allolobophora caliginosa were exposed to 100 mg/kg artificial soil containing CCA-NPs, SCB-NPs or USCB-NPs for 30 days. Each treatment included 3 replicate chambers, each containing 20 adult worms in 1000 g of artificial soilunder controlled laboratory conditions (20 ± 2 °C; 12 h light; 70–80% relative humidity).
Banaee et al. 2024 [47]	Iran	In vivo laboratory experimental study	To evaluate the effects of sublethal concentrations of cigarette-butt leachate on blood biochemical parameters, hepatic oxidative-stress biomarkers, and the biochemical profile of liver and muscle tissues in Nile tilapia after 21 days of exposure.	Plasma biochemical parameters, hepatic oxidative-stress biomarkers, total antioxidant capacity, lipid peroxidation, and Raman-spectroscopy profiles of liver and muscle tissues.	Dechlorinated freshwater (leaching/exposure medium)	Experimental model: laboratory ecotoxicological bioassay Tobacco product investigated: smoked conventional cigarette butts from five brands Exposure conditions: sixty smoked cigarette butts from five brands, weighing approximately 15 g, were immersed in 1 L of water for 72 h. Nile tilapia were exposed to 0, 0.5, 1.0, 1.5 and 2.0 µL/L of cigarette-butt leachate for 21 days.
Cho et al. 2025 [48]	South Korea	In vivo laboratory experimental study	To evaluate the acute and chronic toxicity of cigarette filter-derived microplastics in Daphnia magna and investigate the physiological, reproductive and molecular mechanisms underlying their toxic effects.	Particle characterization, nicotine content, LC-HRMS chemical profiling, immobilisation, mortality, reproduction, time to first brood, offspring body length, oxidative stress, and expression of stress-, detoxification-, endocrine- and moulting-related genes.	OECD freshwater test medium (exposure medium)	Experimental model Laboratory ecotoxicological bioassay Tobacco product investigated Smoked conventional cigarette filters (GSF); non-smoked conventional cigarette filters (CAF). Exposure conditions Acute exposure: *D. magna* neonates (<24 h), 6.25–100 mg/L for 48 h (OECD 202). Chronic exposure: individual daphnids, 6.25 and 12.5 mg/L for 21 days (OECD 211). Test microplastics were obtained by cryomilling smoked and non-smoked cigarette filters.
Couto et al. 2025 [49]	Portugal	In vivo laboratory experimental study	To evaluate the acute and sub-chronic toxicity of smoked and non-smoked cigarette butt elutriates on Artemia franciscanaunder current and projected warming scenarios.	LC_50_, mortality, body growth, metabolic rate, acetylcholinesterase activity, glutathione S-transferase activity, lipid peroxidation, catalase, glutathione peroxidase, glutathione reductase, total glutathione, reduced glutathione and oxidized glutathione.	Artificial seawater (elutriate/exposure medium)	Experimental model: laboratory ecotoxicological bioassay Tobacco product investigated: smoked conventional cigarette butts; non-smoked conventional cigarette butts. Exposure conditions: Acute exposure: *A. franciscana* nauplii, 0.188–4.0 CB/L for 48 h at 25 or 30 °C. Sub-chronic exposure: 0.03125–0.5 CB/L for 7 days at 25 or 30 °C (10 replicates × 6 organisms per treatment).
Ferreira-Griz et al. 2026 [50]	Brazil	In vivo laboratory experimental study	To evaluate the toxicological effects of cigarette butt leachate on the tropical mussel Mytella charruana by assessing lysosomal membrane stability and the bioaccumulation of trace metals and PAHs.	Trace metal concentrations, PAH concentrations, lysosomal membrane stability (Neutral Red Retention Time, NRRT), trace metal bioaccumulation and PAH bioaccumulation.	Artificial seawater (leaching/exposure medium)	Experimental model: laboratory ecotoxicological bioassay Tobacco product investigated: smoked conventional cigarette butts Exposure conditions: *Mytella charruana* (*n* = 160) exposed to serial smoked-CB leachate dilutions (0.01–10%) for 24 and 120 h.
Dolar et al. 2025 [51]	Slovenia	In vivo laboratory experimental study	To investigate and compare the ecotoxicological effects of microplastics produced from smoked cigarette filters and unsmoked cigarette filters on terrestrial and aquatic invertebrates, together with their physicochemical characterization	Particle size, chemical characterization (metals and organic compounds), survival, growth, total haemocyte count, haemocyte viability, haemocyte differentiation, acetylcholinesterase, glutathione S-transferase, electron transfer system.	Lufa 2.2 agricultural soil and freshwater media (exposure media)	Experimental model Laboratory ecotoxicological bioassay Tobacco product investigated Smoked conventional cigarette filters and unsmoked conventional cigarette filters. Exposure conditions Microplastics obtained by cryo-milling smoked and unsmoked cigarette filters were characterized and tested on terrestrial and aquatic invertebrates. Porcellio scaber and Tenebrio molitor were exposed in Lufa 2.2 agricultural soil containing 0.06%, 0.5% and 1.5% (*w*/*w*) MPs for 14 and 28 days, respectively. Daphnia magna and Brachionus calyciflorus were exposed in freshwater media to 1, 10 and 100 mg/L MPs for 96 h and 24 h, respectively. Biomarkers, growth and survival were evaluated.
Green et al. 2020 [52]	United Kingdom	In vivo laboratory experimental study	To assess lethal and sublethal effects of increasing concentrations of leachate from smoked cigarettes containing either conventional cellulose acetate filters or biodegradable cellulose filters on four freshwater invertebrate species, and to determine whether biodegradable cellulose-filter butts are less harmful than conventional cellulose acetate butts.	Adult mortality/time to death; juvenile *B. tentaculata* survival; and sublethal behavioural responses, including movement, filtering activity and open/closed behavioural states, assessed across exposure concentrations, filter types and exposure times.	Filtered rainwater/freshwater (leaching/exposure medium)	Laboratory leaching experiment; mesocosm exposure experiment. Tobacco product investigated: Artificially smoked cigarettes with two different filter materials: conventional cellulose acetate filters versus biodegradable unbleached cellulose filters. Exposure conditions: Separate stock leachates were prepared by soaking 14 smoked butts/L of either cellulose acetate or cellulose filters in filtered rainwater for 18 h at approximately 18 °C, with gentle agitation at 100 rpm. Mesocosms contained 400 mL freshwater and were exposed to 0 (control), 0.2, 1, or 5 smoked butts/L of either filter type. Each treatment had 5 replicate mesocosms (*n* = 5; N = 35). Water was maintained at approximately 15 °C, pH 8.13, salinity <0.05 ppt, under a 12:12 h light/dark cycle. Exposure lasted 120 h, with mortality and behaviour assessed every 24 h. Each mesocosm contained one adult *Dreissena polymorpha*, *Polycelis nigra*, *Planorbis planorbis* and *Bithynia tentaculata*, plus five juvenile *B. tentaculata*.
Kim et al. 2023 [53]	Republic of Korea	In vivo laboratory experimental study	To assess the toxicity of microfibers derived from smoked CBs, with specific focus on exoskeletal alterations in the whiteleg shrimp *Penaeus vannamei*, including effects on the carapace and molting-related responses.	Wet weight; body length; cumulative molting; carapace structure and thickness; relative endocuticle/exocuticle thickness; carapace Ca and Mg content and Ca:Mg ratio; body/carapace pigmentation, including SSI and CI; gill histopathology and lamellar tissue density.	Artificial seawater (exposure medium)	Experimental model: Laboratory ecotoxicological bioassay. Tobacco product investigated: Microfibers derived from artificially smoked conventional cigarette butts with cellulose acetate filters. Exposure conditions: Shrimp weighing 0.6–0.9 g were exposed to CB-derived microfibers at a single concentration of 1.86 mg/L for 28 days. Three individual shrimp were housed separately in 1-L beakers, with three replicate setups for both control and exposed groups (18 shrimp in total). Artificial seawater was maintained at 25 °C, aerated to maintain dissolved oxygen at 6.7–7.52 mg/L, and renewed every 7 days. Shrimp were fed daily with pellets corresponding to 5% of body mass.
Lian et al. 2025 [54]	China	In vivo laboratory experimental study	To investigate the multi-level toxic effects of cigarette butts on the marine rotifer Brachionus plicatilis by integrating behavioral, metabolic and mitochondrial endpoints; specifically, to assess inhibition of feeding and motility, elucidate the pathway linking oxidative stress to mitochondrial dysfunction and lipid-metabolism disturbance, and compare the toxicity mechanisms of CB fibers and leachates from different cigarette-butt types, with particular attention to residual ash from smoked cigarettes.	Feeding behavior (grazing and filtration rates); swimming behavior (velocity, acceleration and rotation angle); oxidative stress (ROS, SOD, CAT and MDA); lipid metabolism (neutral lipid content, AMS and LPS activities, TG content); mitochondrial function (ATP and MMP); lysosomal function and autophagy; expression of genes involved in mitophagy and mitochondrial fission/fusion; effects of antioxidant NAC treatment; correlations among biomarkers; and integrated biomarker response index (BRI).	Artificial seawater (leaching/exposure medium)	Experimental model: Laboratory leaching experiment; laboratory ecotoxicological bioassay. Tobacco product investigated: Three cigarette-butt-derived materials were compared: SCB = smoked cigarette butts with 1.0 cm residual tobacco; SF = smoked cigarette filters without residual tobacco; USF = unsmoked cigarette filters without tobacco residues. Exposure conditions: CB leachates were prepared in artificial seawater at 25 °C for 7 days. Rotifers were subsequently exposed for 24 h to fibers or leachates from SCB, SF or USF at 0.03, 0.3 and 3 CB/L, plus a 0 CB/L control. The lowest concentration (0.03 CB/L) was selected as environmentally relevant to Yellow Sea coastal waters, whereas 3 CB/L represented an extreme pollution scenario. Treatments generally included at least three biological replicates.
Olah-Kovacs et al. 2025 [55]	United Kingdom	In vivo laboratory experimental study.	To investigate the effects of smoked CB leachate on survival, reproductive success, growth, and feeding rate of L. stagnalis, and to assess the potential for recovery after cessation of exposure.	Mortality and survival probability; shell-length and fresh-biomass growth; feeding rate; clutch production; clutch size; egg production; egg viability/hatching success; egg abnormalities; post-exposure recovery.	ISO freshwater medium (leaching/exposure medium)	Experimental model: Laboratory leaching experiment; laboratory ecotoxicological bioassay. Tobacco product investigated: Field-collected smoked conventional cigarette butts. Exposure conditions: A stock leachate was prepared by leaching 30 CBs in 2 L ISO medium for six days under orbital shaking at 150 rpm. *L. stagnalis* were exposed for four weeks to 0 (control), 0.25, 0.5, or 1 CB L^−1^, followed by a three-week recovery period in uncontaminated ISO medium. The lowest treatment was selected as a high but environmentally realistic concentration.
Srikanth et al. 2025 [56]	India	In vivo laboratory experimental study.	To investigate the toxic effects of cigarette butt leachate on the freshwater snail *Pila virens* by assessing changes in oxidative stress, antioxidant defense, and neurotoxicity biomarkers following exposure to different leachate concentrations for 24 and 48 h.	Protein carbonyl content; glutathione peroxidase activity; reduced glutathione content; glutathione S-transferase activity; acetylcholinesterase activity; lipid peroxidation; concentration- and time-related changes in oxidative stress, antioxidant defense, and neurotoxicity biomarkers.	Freshwater/distilled water (leaching/exposure media)	Experimental model: Laboratory leaching experiment; laboratory ecotoxicological bioassay. Tobacco product investigated: Smoked conventional cigarette butts. Exposure conditions: Cigarette butt stock solution was prepared by soaking 5 smoked cigarette butts/L water for 2 h, followed by magnetic stirring and filtration through Whatman No. 1 paper. The stock solution was diluted with distilled water to obtain 10%, 25%, and 50% *v*/*v* exposure concentrations. *P. virens* were exposed for 24 and 48 h; 10 snails were used for each concentration and control group, and experiments were conducted three times for each concentration. Before exposure, snails were acclimated for 15 days under laboratory conditions at 21 ± 1 °C, 12:12 h light/dark photoperiod and pH 7.30 ± 0.02.

Note. CB: cigarette butts. Table 1, Table 2 and Table 3 include only studies classified as addressing a single One Health domain. Studies addressing two or more One Health domains were classified as multi-domain and are reported exclusively in Table 4; therefore, no study is duplicated across Table 1, Table 2, Table 3 and Table 4. * Environmental matrix/experimental medium indicates, as applicable, the environmental matrix directly sampled or experimentally used, the field sampling setting, or the medium used for leaching, exposure, degradation, or other experimental procedures. The specific function is reported in parentheses.

**Table 4:** Characteristics of the studies included in the systematic review addressing the multiple One Health domain.

Author Year	Country	Study Design	Objective	Outcomes Assessed	Environmental Matrix/Experimental Medium *	Experimental Model Tobacco Product Investigated Exposure Conditions	**One Health Domain**
Belzagui et al. 2021 [57]	Spain	In vivo laboratory experimental study	To evaluate the release rate, acute aquatic toxicity, and aquatic thermo-oxidative and photo-degradability of cellulose acetate microfibres detached from smoked cigarette filters, and to estimate their potential contribution to global microfiber pollution.	Microfiber release rate, microfiber size, acute immobilisation of Daphnia magna, EC50, EC100, physical entanglement, chemical-structure changes, mass loss and degradability.	Distilled water (leaching/release medium); synthetic seawater and freshwater (degradation media); freshwater (ecotoxicity exposure medium)	Experimental model: laboratory ecotoxicological bioassay Tobacco product investigated: smoked filters from four conventional cigarette brands; unsmoked conventional cigarette filters were used in the degradation experiments. Exposure conditions Microfiber release: 1 SF/L in distilled water, continuous slow agitation, 2 weeks. Ecotoxicity: filtered leachate vs leachate containing ~1-mm microfibres; 0.010–10 SF/L; *D. magna*, 48 h (4 replicates × 5 organisms). Degradability: distilled water/synthetic seawater + UV/agitation (1 month); freshwater + natural sunlight (18 months).	Environmental health + Animal health
Bonanomi et al. 2026 [58]	Italy	In vivo laboratory ecotoxicological bioassay	To investigate the long-term decomposition of cigarette butts over a 10-year period by evaluating mass loss, chemical transformations, microbial succession and ecotoxicological changes under different environmental conditions.	Mass loss, nitrogen dynamics, chemical transformations (^13^C CPMAS NMR), microbial diversity and community composition, ecotoxicity (bioluminescence inhibition, algal growth inhibition, immobilisation, morphological alterations and root elongation), VOC profiles and SEM-EDX morphology.	Grassland and sand-dune soils (degradation media); freshwater (leaching/ecotoxicity medium)	Experimental model: Laboratory ecotoxicological bioassay Tobacco product investigated Smoked conventional cigarette butts; unsmoked conventional cigarette butts (reference samples). Exposure conditions Degradation: 12,000 smoked cigarette butts incubated under five laboratory/field conditions (no soil, grassland soil, or sand dune soil) for 30, 90, 180, 360, 720, 1080, 1800, and 3600 days. Ecotoxicity: leachates tested on *A. fischeri*, *R. subcapitata*, *D. magna*, *H. vulgaris*, and *S. saccharatum*.	Environmental health + Animal health + Plant health
Lazo et al. 2024 [59]	Chile	In vitro laboratory experimental study	To evaluate the environmental impact of cigarette butt leachates on coastal ecosystems by assessing their phytotoxicity to *Lactuca sativa* and *Lolium perenne*, and to characterize chemical changes and metal release under experimental conditions simulating cigarette butt contamination of coastal sand.	Leachate pH; FTIR characterization; Fe, Zn and Cu concentrations by atomic absorption spectroscopy; seed germination; radicle and shoot growth; Relative Germination Percentage (RGP); Germination Index (GI); Relative Radicle Growth (RRG); Hypocotyl/Coleoptile Growth (HG); and toxicity classification based on normalized germination/root-elongation indices.	Natural beach sand (sampled/experimental matrix); ultrapure deionized water (leaching medium)	Experimental model: Laboratory leaching experiment; laboratory ecotoxicological bioassay Tobacco product investigated: Smoked conventional cigarette butts. Exposure conditions: Three experimental scenarios were evaluated in triplicate: (A) sand only, (B) CBs only, and (C) CBs + sand. Scenarios containing CBs received 6 g of ground CB material, while sand-containing scenarios received 200 g of cleaned and dried natural beach sand. Leachates were prepared with 500 mL ultrapure deionized water, gently agitated for 10 min and allowed to leach for 24 h; leachate collection was repeated three times to simulate continued environmental exposure. Final leachates collected on day 37 were used for phytotoxicity testing. Twenty seeds of each plant species were exposed to 5 mL leachate for 120 h under species-specific germination conditions.	Environmental health + Plant health
Lucia et al. 2023 [7]	Italy	Multicomponent laboratory experimental study (ecotoxicological bioassays, ex vivo and in vivo) with chemical characterization and quantitative WOE assessment	To characterize the toxicological effects of cigarette butts on marine organisms across different levels of biological organization and to integrate chemical, ecotoxicological, and biomarker evidence through a quantitative Weight of Evidence approach (WOE).	Chemical contaminant release, ecotoxicological effects, bioaccumulation, biomarker responses, and integrated ecotoxicological risk (WOE).	Artificial seawater (leaching/exposure medium)	Experimental model: Laboratory leaching experiment; laboratory ecotoxicological bioassay; whole-cigarette butt exposure bioassay. Tobacco product investigated Naturally smoked cigarette butts from different brands, consisting of intact filters, covering paper, residual unsmoked tobacco and ash, collected from covered cigarette receptacles within one week after smoking. Exposure conditions: For leaching, CBs were soaked in artificial seawater at 100 CBs/L (LEAC_A) and 25 CBs/L (LEAC_B) for 24 h at 18–20 °C under agitation, with three replicates. Serial dilutions of both leachates were used for standardized ecotoxicological bioassays. Ex vivo PCTS were exposed to 50% LEAC_A, corresponding to 50 CBs/L, for 24, 48 and 72 h at 18 °C. For the whole-CB in vivo experiment, 240 M. galloprovincialis were divided among tanks containing 0, 0.5, 1 or 5 CBs/L in artificial seawater for 14 days, with water and CBs renewed every 72 h.	Environmental health + Animal health
Mihajlović et al. 2026 [60]	Bosnia and Herzegovina	Laboratory experimental study	To quantify Pb, Cd, Cr and Mn in smoked and unsmoked butts from conventional cigarettes and used HEETS tobacco sticks from a heated tobacco product (referred to as “e-cigarette butts” in the original study), characterize their leaching kinetics under different acidic conditions, and estimate potential carcinogenic and non-carcinogenic human health risks associated with metals released into water.	Total Pb, Cd, Cr and Mn content; metal concentrations released during leaching; effects of pH, leaching agent and contact time; leaching reaction order and half-life; carcinogenic risk for Cd and Cr (ILCR); non-carcinogenic risk for Pb and Mn (HQ); ingestion and dermal exposure pathways; separate risk estimates for adults and children.	Simulated acidic water/precipitation (leaching medium)	Experimental model: Laboratory leaching experiment and human health risk assessment. Tobacco product investigated: Smoked and unsmoked conventional cigarette butts and HEETS tobacco sticks from a heated tobacco product (referred to as “e-cigarette butts” in the original study).. Exposure conditions: For the leaching experiment, four Heets HEETS tobacco-stick samplesand two Chesterfield Blue conventional CB samples were exposed to 25 mL of acidic solutions under two experimental series. Series 1 used 0.1 M CH_3_COOH (pH 4.10), 0.1 M HCl (pH 1.23) and 1 M HCl (pH 0.23); Series 2 used 0.1 M HNO_3_ (pH 1.99), 1 M HNO_3_ (pH 0.28) and 2 M HNO_3_ (pH 0.18). Leaching was evaluated over approximately 0–28 days, with 3-h agitation at 25 °C and 200 rpm at the sampling points.	Environmental health + Human health
Nitschke et al. 2023 [61]	Sweden	Combined field and in vivo laboratory study	To investigate personal and contextual predictors of cigarette littering and assess the acute and chronic toxicity of smoked and unsmoked cigarette filters to the freshwater invertebrate Chironomus riparius, including potential drivers of filter toxicity.	Cigarette-littering behaviour and personal/contextual predictors; acute larval immobility; chronic mortality, growth and development; frequency and severity of mouthpart deformities; and differences according to smoked versus unsmoked filters, filter fibres versus leachates, and water versus sediment exposure.	Public urban environments (sampling setting); dechlorinated tap water and sediment–water system (leaching/exposure media)	Experimental model: Field survey; laboratory ecotoxicological bioassay. Tobacco product investigated: Smoked and unsmoked conventional cigarette filters. Exposure conditions: Acute toxicity was assessed by exposing first-instar *C. riparius* larvae for 48 h to smoked or unsmoked cigarette-filter material at 0.125, 0.25, 0.5, 1, and 2 filter equivalents/L, using three exposure types: unwashed fibres, filter leachates, and washed fibres. Leachates were prepared by stirring filter fibres in dechlorinated tap water for 24 h. For chronic exposure, whole smoked or unsmoked filters were leached for 3 weeks in a sediment–water system at 1 filter/L water and 1 filter/166.5 mL sediment; larvae were subsequently exposed separately to the contaminated water or sediment for 7 days.	Environmental health + Animal health
Nongdren et al. 2024 [62]	India	Environmental risk assessment study.	To assess cigarette butt littering in Aizawl city, identify spatial and seasonal littering patterns and hotspots, estimate the potential contribution of littered cigarette butts to downstream surface-water pollution using a newly developed water pollution potential index, and assess potential human health risks associated with ingestion of cigarette-butt-derived contaminants.	Cigarette butt density and spatial/seasonal distribution; littering hotspots according to land use; WPPI; potential contaminant concentrations in sewage/surface water; CDI and lifetime intake through ingestion; NCR; HQ and HI; CR; and relative contributions of heavy metals, PAHs, BTEX compounds, aromatic amines, and nicotine to estimated exposure and risk.	Urban roadside and sewer environment (sampling setting); municipal sewage and downstream surface water (modelled environmental compartments)	Experimental model: Field survey. Tobacco product investigated: Littered smoked cigarette butts. Exposure conditions: Not applicable for the field component.	Environmental health + Human health
Nongdren et al. 2026 [63]	India	Environmental risk assessment study.	To quantify the environmental impacts of cigarettes across the full cradle-to-grave life cycle, including upstream production, consumption, and end-of-life phases; identify environmental hotspots; compare alternative cigarette-butt end-of-life management strategies and biodegradable filter materials; and assess the sensitivity and uncertainty of the model results.	Life-cycle midpoint and endpoint environmental impacts; environmental hotspots across production, consumption, and end-of-life phases; freshwater, marine and terrestrial ecotoxicity; eutrophication; human carcinogenic and non-carcinogenic toxicity; global warming and resource-related impacts; relative environmental performance of alternative end-of-life strategies and biodegradable filter materials; and sensitivity and uncertainty of modelled impacts.	Air, terrestrial, freshwater and marine environments (modelled environmental compartments)	Experimental model: Not applicable. Tobacco product investigated: Conventional cigarettes with cellulose acetate filters and smoked cigarette butts; alternative biodegradable cigarette filters composed of flax, hemp, jute, and cotton were additionally modelled. Exposure conditions: Not applicable.	Environmental health + Human health
Oliva et al. 2021 [64]	Italy	In vivo laboratory experimental study.	To evaluate the toxicity of smoked and unsmoked cigarette butt leachates at different concentrations using batteries of ecotoxicological bioassays representative of marine and freshwater environments.	Algal growth inhibition; bacterial bioluminescence inhibition; larval development inhibition; crustacean immobilization/survival; plant germination and root growth; Daphnia magna heartbeat rate; EC10, EC20, and EC50 values.	Natural seawater and synthetic freshwater (leaching/exposure media)	Experimental model: Laboratory leaching experiment; laboratory ecotoxicological bioassay. Tobacco product investigated: Smoked and unsmoked conventional cigarette filters. Exposure conditions: Leachates were prepared at 20 CBs/L using natural seawater adjusted to 30‰ salinity or standard moderately hard synthetic freshwater. Cigarette filters were leached for 24 h in darkness at 20 ± 2 °C under orbital shaking, after which leachates were filtered. Marine and freshwater bioassays were performed at 100%, 75%, 50%, 25%, and 10% leachate, corresponding to 20, 15, 10, 5, and 2 CBs/L; higher-plant assays were performed only at the maximum freshwater concentration. D. magna heartbeat was assessed after 24, 48, and 72 h exposure.	Environmental health + Animal health + Plant health
Piccardo et al. 2021 [65]	Italy	In vivo laboratory experimental study	To assess and compare the ecotoxicity of smoked and unsmoked classic cigarette butt leachates and materials described by the original authors as “electronic cigarette butts”, and to investigate how weathering conditions and duration influence the toxicity of elutriates from cigarette-butt-contaminated sandy sediments.	Bioluminescence inhibition; microalgal growth inhibition; sea urchin fertilization inhibition; larval development abnormalities; larval body-size reduction; integrated ecotoxicological Hazard Quotient and Class of Hazard; effects of cigarette type, smoking status, weathering conditions, and weathering duration on ecotoxicity.	Natural sandy marine sediment (sampled/experimental matrix); artificial seawater (leaching/exposure medium)	Experimental model: Laboratory leaching experiment; laboratory ecotoxicological bioassay. Tobacco product investigated: Smoked and unsmoked materials described by the original authors as “electronic cigarette butts”; the specific electronic-cigarette product/device associated with these materials was not clearly identified in the study. Exposure conditions Leachates: smoked/unsmoked CCBs and ECBs, 10 CB/L ASW, 1 h at 100 rpm; sediment weathering:smoked/unsmoked CCBs, 0.11 CCB/L natural sandy sediment, dry vs. simulated rain for 1 or 2 weeks; sediment elutriates prepared in ASW (1:4 *w*/*v*). Bioassay exposure: *A. fischeri* 15–30 min; *P. tricornutum* 72 h; *P. lividus* fertilization 20 min and larval development 72 h.	Environmental health + Animal health
Soleimani et al. 2023 [66]	Iran	In vivo laboratory experimental study	To assess the chemical contents and toxic effects of three types of cigarette butt leachates—smoked cigarette butts with residual tobacco, smoked filters without residual tobacco, and unsmoked filters—on Periophthalmus waltoni at different exposure times.		Natural seawater (leaching/exposure medium)	Experimental model: Laboratory leaching experiment; laboratory ecotoxicological bioassay. Tobacco product investigated: Smoked cigarette butts with residual tobacco; smoked cigarette filters without residual tobacco; unsmoked cigarette filters. Exposure conditions: Cigarette butts/filters were soaked in natural seawater for 24 h. P. waltoni juveniles were exposed to five concentrations for each leachate type: SCB: 0.25, 0.5, 1, 2, and 4 CB/L; SF: 0.5, 1, 2, 4, and 8 CB/L; USF: 1, 2, 4, 8, and 16 CB/L. Mortality was assessed at 24, 48, 72, and 96 h. Four replicates with five fish per replicate were used for each concentration. Fish were acclimated for two weeks before exposure and experiments were conducted at approximately 25 °C, 35‰ salinity, with continuous aeration and a 12:12 h light/dark cycle.	Environmental health + Animal health
Torkashvand et al. 2021 [67]	Iran	Laboratory experimental study	To assess the effect of cigarette butts in municipal solid waste on heavy metal concentrations in landfill leachate and to estimate the associated human health and ecological risks for Cd, Cr, Ni, Pb, and Zn.	Concentrations of Cd, Cr, Ni, Pb, and Zn in landfill leachate; total heavy metal concentration; non-carcinogenic human health risk through ingestion of leachate-contaminated groundwater; HQ; cumulative HQ; ecological risk associated with heavy metals; and changes in health and ecological risk under different proportions of freshly smoked and littered CBs.	Synthetic municipal solid waste (experimental matrix); landfill leachate (analytical matrix)	Experimental model: Laboratory leaching experiment. Tobacco product investigated: Freshly smoked cigarette butts and littered cigarette butts. Exposure conditions: Three pilot-scale landfills with identical characteristics were filled with 5.5 kg of synthetic commingled municipal solid waste based on the composition of Tehran municipal waste. P1 served as the control without CBs; P2 contained 140 freshly smoked CBs (0.76% *w*/*w*); and P3 contained 1.3% w/w littered CBs. Leachate was collected from the landfill pilots and Cd, Cr, Ni, Pb, and Zn were quantified. Risk calculations also evaluated standardized scenarios containing 1% *w*/*w* CBs with different proportions of freshly smoked and littered CBs: 25:75 (S1), 50:50 (S2), and 75:25 (S3). For human-health risk assessment, leachate was assumed to undergo 100-fold dilution in groundwater, with drinking-water ingestion used as the exposure pathway.	Environmental health + Human health

* Environmental matrix/experimental medium indicates, as applicable, the environmental matrix directly sampled or experimentally used, the field sampling setting, or the medium used for leaching, exposure, degradation, or other experimental procedures. The specific function is reported in parentheses. Note. Table 4 includes studies addressing two or more One Health domains. The specific combination of domains directly addressed by each study is reported in the “One Health domain” column. Multi-domain studies are reported exclusively in this table and are not duplicated in Table 1, Table 2 and Table 3. No study was classified exclusively within the Human health domain.

## Data Availability

No new data were created or generated in this study. All data analyzed in this systematic review were extracted from the published studies included in the review. The data supporting the findings are provided within the article and its Supplementary Materials.

## Supplementary Materials

The following supporting information can be downloaded at: https://www.mdpi.com/article/10.3390/toxics14100846/s1, Table S1: Review-specific methodological quality assessment checklist for laboratory experimental studies that are neither in vivo nor in vitro; Table S2a: ToxRTool reliability assessment of the in vitro components of the included studies (N = 7); Table S2b: ToxRTool reliability assessment of the in vivo components of the included studies (N = 20); Table S3: Methodological quality assessment of environmental and non-in vivo/non-in vitro studies (N = 22); Table S4: Main findings, dose–effect relationships, and study limitations of the included studies investigating the environmental and ecotoxicological impacts of cigarette butt and related tobacco product waste.

## Abbreviations

The following abbreviations are used in this manuscript:

**Abbreviation**	**Definition**
ASW	Artificial seawater
BTEX	Benzene, toluene, ethylbenzene, and xylenes
CBs	Cigarette butts
CF	Cigarette filter
DW	Deionized water
EC50	Median effective concentration
ENDS	Electronic nicotine delivery systems
EU	European Union
HHRA	Human health risk assessment
ISO	International Organization for Standardization
LCA	Life cycle assessment
LC50	Median lethal concentration
N/A	Not applicable
NPs	Nanoplastics
OCPs	Organochlorine pesticides
OHAT	Office of Health Assessment and Translation
OHHLEP	One Health High-Level Expert Panel
PAHs	Polycyclic aromatic hydrocarbons
PCBs	Polychlorinated biphenyls
PECO	Population, Exposure, Comparator, and Outcomes
PRISMA	Preferred Reporting Items for Systematic Reviews and Meta-Analyses
PROSPERO	International Prospective Register of Systematic Reviews
QA/QC	Quality assurance/quality control
RAGS	Risk Assessment Guidance for Superfund
RW	River water
SW	Seawater
ToxRTool	Toxicological Data Reliability Assessment Tool
TW	Tap water
USEPA	United States Environmental Protection Agency
VOCs	Volatile organic compounds

## References

[1] Araújo M.C.B., Costa M.F. (2019). A critical review of the issue of cigarette butt pollution in coastal environments. Environ. Res..

[2] Benavente M.J., Caballero M.J.A., Silvero G., López-Coca I., Escobar V.G. (2018). Cellulose Acetate Recovery from Cigarette Butts. Proceedings.

[3] Torkashvand J., Farzadkia M., Sobhi H.R., Esrafili A. (2020). Littered cigarette butt as a well-known hazardous waste: A comprehensive systematic review. J. Hazard. Mater..

[4] Da Silva N.F., De Araújo M.C.B., Silva-Cavalcanti J.S. (2023). Cigarette butts in the environment: A growing global threat?. Environ. Rev..

[5] Bonanomi G., Maisto G., De Marco A., Cesarano G., Zotti M., Mazzei P., Libralato G., Staropoli A., Siciliano A., De Filippis F. (2020). The fate of cigarette butts in different environments: Decay rate, chemical changes and ecotoxicity revealed by a 5-years decomposition experiment. Environ. Pollut..

[6] Acarer Arat S. (2024). A review on cigarette butts: Environmental abundance, characterization, and toxic pollutants released into water from cigarette butts. Sci. Total Environ..

[7] Lucia G., Giuliani M.E., d’Errico G., Booms E., Benedetti M., Di Carlo M., Fattorini D., Gorbi S., Regoli F. (2023). Toxicological effects of cigarette butts for marine organisms. Environ. Int..

[8] Green D.S., Boots B., Da Silva Carvalho J., Starkey T. (2019). Cigarette butts have adverse effects on initial growth of perennial ryegrass (gramineae: *Lolium perenne* L.) and white clover (leguminosae: *Trifolium repens* L.). Ecotoxicol. Environ. Saf..

[9] Pitt S.J., Gunn A. (2024). The One Health Concept. Br. J. Biomed. Sci..

[10] Banks E., Yazidjoglou A., Brown S., Nguyen M., Martin M., Beckwith K., Daluwatta A., Campbell S., Joshy G. (2023). Electronic cigarettes and health outcomes: Umbrella and systematic review of the global evidence. Med. J. Aust..

[11] El-Kaassamani M., Yen M., Talih S., El-Hellani A. (2024). Analysis of mainstream emissions, secondhand emissions and the environmental impact of IQOS waste: A systematic review on IQOS that accounts for data source. Tob. Control.

[12] World Health Organization (2025). WHO Global Report on Trends in Prevalence of Tobacco Use 2000–2024 and Projections 2025–2030.

[13] Beutel M.W., Harmon T.C., Novotny T.E., Mock J., Gilmore M.E., Hart S.C., Traina S., Duttagupta S., Brooks A., Jerde C.L. (2021). A Review of Environmental Pollution from the Use and Disposal of Cigarettes and Electronic Cigarettes: Contaminants, Sources, and Impacts. Sustainability.

[14] Page M.J., McKenzie J.E., Bossuyt P.M., Boutron I., Hoffmann T.C., Mulrow C.D., Shamseer L., Tetzlaff J.M., Akl E.A., Brennan S.E. (2021). The PRISMA 2020 statement: An updated guideline for reporting systematic reviews. Int. J. Surg..

[15] Bernardo W.M. (2017). PRISMA statement and PROSPERO. Int. Braz. J. Urol..

[16] Posso M., Sala M. (2024). PROSPERO—Reasons for its existence and why a systematic review and/or meta-analysis should be registered. Cir. Esp. Engl. Ed..

[17] Morgan R.L., Whaley P., Thayer K.A., Schünemann H.J. (2018). Identifying the PECO: A framework for formulating good questions to explore the association of environmental and other exposures with health outcomes. Environ. Int..

[18] Ouzzani M., Hammady H., Fedorowicz Z., Elmagarmid A. (2016). Rayyan—A web and mobile app for systematic reviews. Syst. Rev..

[19] Adisasmito W.B., Almuhairi S., Behravesh C.B., Bilivogui P., Bukachi S.A., Casas N., Cediel Becerra N., Charron D.F., Chaudhary A., One Health High-Level Expert Panel (OHHLEP) (2022). One Health: A new definition for a sustainable and healthy future. PLoS Pathog..

[20] Schneider K., Schwarz M., Burkholder I., Kopp-Schneider A., Edler L., Kinsner-Ovaskainen A., Hartung T., Hoffmann S. (2009). “ToxRTool”, a new tool to assess the reliability of toxicological data. Toxicol. Lett..

[21] National Toxicology Program (2019). Handbook for Conducting a Literature-Based Health Assessment Using OHAT Approach for Systematic Review and Evidence Integration.

[22] Arfaeinia H., Masjedi M.R., Asgariyan R., Soleimani F., Alipour V., Dadipoor S., Saeedi R., Jahantigh A., Maryamabadi A. (2024). Release of polychlorinated biphenyls (PCBs) and organochlorine pesticides (OCPs) from cigarette butts into the aquatic environment: Levels and ecotoxicity. Heliyon.

[23] Kim L., Kim S.A., An Y.-J. (2023). Microfibers from cigarette butts can induce exoskeletal alteration in whiteleg shrimp (*Penaeus vannamei*). Mar. Pollut. Bull..

[24] Dobaradaran S., Schmidt T.C., Kaziur-Cegla W., Jochmann M.A. (2021). BTEX compounds leachates from cigarette butts into water environment: A primary study. Environ. Pollut..

[25] Dobaradaran S., Telgheder U., De-la-Torre G.E., Rockel S.P., Mutke X.A.M., Schmidt T.C. (2024). Elucidating nicotine transfer into water environments via cigarette butt remaining parts. Environ. Pollut..

[26] Dobaradaran S., Mutke X.A.M., Schmidt T.C., Swiderski P., De-la-Torre G.E., Jochmann M.A. (2022). Aromatic amines contents of cigarette butts: Fresh and aged cigarette butts vs unsmoked cigarette. Chemosphere.

[27] Tan Q., Li J., Liu L., Zhan K., Shi G. (2026). Neglected plastic waste from discarded cigarette butts: A case study on hazards identification in typical regions in China. Environ. Res..

[28] Yang S., Gu C., Jiao Y., Yang Q. (2023). Research on the presence of cigarette butts and their leaching of chemical pollutants and microparticles: The case of Dalian, China. Front. Mar. Sci..

[29] Mihajlović D., Lončar N., Đurić A., Đekanović B., Jelić D. (2026). Exploring the heavy metals (Pb, Cd Cr, and Mn) content, kinetic dynamics and health risks in the cigarette butts in Bosnia and Herzegovina. Int. J. Environ. Anal. Chem..

[30] Caridi F., Sabbatini A., Birarda G., Costanzi E., De Giudici G., Galeazzi R., Medas D., Mobbili G., Ricciutelli M., Ruello M.L. (2020). Cigarette butts, a threat for marine environments: Lessons from benthic foraminifera (Protista). Mar. Environ. Res..

[31] El Hadri H., Lisa J.M., Gigault J., Reynaud S., Grassl B. (2021). Fate of nanoplastics in the environment: Implication of the cigarette butts. Environ. Pollut..

[32] Dos Santos N.P., Maciel M.G.R., Guimarães P.S., Trindade C.R.T., Schneck F. (2024). Negative effects of cigarette butt leachate on freshwater phytoplankton communities. Ecotoxicology.

[33] Guttmann N., Wolinska J., Spahr S., Martínez-Ruiz E.B. (2024). Cigarette butts enable toxigenic cyanobacteria growth by inhibiting their lethal fungal infections. Ecotoxicol. Environ. Saf..

[34] Kouhi K., Abbasi-Tajaddod A., Gholami-Borujeni F. (2025). Assessing cigarette butt pollution on recreational beaches: A comparative study of two sampling methods and their impact on metal release. Mar. Pollut. Bull..

[35] Martínez-Ruiz E.B., Fastner J., Spahr S., Wolinska J. (2026). Synergistic and Antagonistic Effects of Chemical Pollutants and Parasitic Fungi on Cyanobacterial Metabolism. Environ. Toxicol..

[36] Michael M., Meyyazhagan A., Velayudhannair K., Pappuswamy M., Maria A., Xavier V., Balasubramanian B., Baskaran R., Kamyab H., Vasseghian Y. (2022). The Content of Heavy Metals in Cigarettes and the Impact of Their Leachates on the Aquatic Ecosystem. Sustainability.

[37] Garshasbi F., Kazemi Miraki S., Jokar Z., Maity A., Ramavandi B. (2026). Quantitative assessment and spatial distribution of macroplastic and cigarette butt contamination in Bushehr’s stormwater system near the sensitive Persian Gulf coast. Mar. Pollut. Bull..

[38] Dobaradaran S., Schmidt T.C., Lorenzo-Parodi N., Kaziur-Cegla W., Jochmann M.A., Nabipour I., Lutze H.V., Telgheder U. (2020). Polycyclic aromatic hydrocarbons (PAHs) leachates from cigarette butts into water. Environ. Pollut..

[39] Bakr Z., Aboulnasr G.N., El-Aal M.A., Elshimy N.A., Said S.M. (2025). Ecotoxicological effects of cellulose acetate nanoplastic derived from cigarette butts on earthworm (*Allolobophora caliginosa*): Implications for soil health. Environ. Res..

[40] Dobaradaran S., Salemi A., De-la-Torre G.E., Telgheder U., Schmidt T.C. (2024). The role of different remaining parts of cigarette butts in the transfer of phenolic compounds into the aquatic environment and their ecological risk. Sci. Total Environ..

[41] Banaee M., Beitsayah A., Zeidi A., Haghi B.N., Piccione G., Faggio C., Multisanti C.R., Impellitteri F. (2024). Toxicity of cigarette butts (CBs) leachate on Nile tilapia (*Oreochromis niloticus*): Blood biochemical parameters, oxidative stress biomarkers, and metabolic profile. Ecotoxicol. Environ. Saf..

[42] Yona D., Firdausi M.A.D., Ramadhani A.N., Sari S.H.J., Yanuar A.T., Chuan O.M. (2025). Spatial distribution and heavy metal leaching assessment of cigarette butts in coastal environments: Evidence from Prigi Bay, Indonesia. J. Hazard. Mater. Adv..

[43] Green D.S., Boots B., Olah-Kovacs B., Palma-Diogo D. (2023). Disposable e-cigarettes and cigarette butts alter the physiology of an aquatic plant *Lemna minor* (Lemnaceae). Sci. Total Environ..

[44] Jakimiuk A., Bulak A., Martínez Barroso P., Podlasek A., Vaverková M. (2022). Impact of Cigarette Butts on Plant Germination Based on *Sinapis alba* L. and *Hordeum vulgare* L. Seeds. J. Ecol. Eng..

[45] Sadıç E., Çelik T.A. (2025). Environmental hazards of light cigarette butts and filters: Physiological, cytotoxic, and genotoxic assessment using the *Allium cepa* L. assay. Environ. Sci. Pollut. Res..

[46] Prata J.C., da Costa J.P., Lopes I., Duarte A.C., Rocha-Santos T. (2020). Environmental exposure to microplastics: An overview on possible human health effects. Sci. Total Environ..

[47] Couto S., Rodrigues S., Patrão R., Vieira M., Antunes S.C., Pinheiro C. (2025). Impact of cigarette butts elutriates on Artemia franciscana in a climate change context. Mar. Pollut. Bull..

[48] Cho H., Sung S., Lim H., Chung S., Kim Y.J., Lim H.-B., Kim Y. (2025). Toxicological Assessment of Cigarette Filter-Derived Microplastics in Daphnia Magna. SSRN.

[49] Iturralde K., Karunarathne S., Selby P., Zawertailo L. (2026). A Review of the Environmental Impacts of Post-Consumer Electronic Cigarette Waste and Disposal. Nicotine Tob. Res..

[50] Ferreira-Griz J.D.A., Taniguchi S., Bícego M.C., Castro Í.B., Zanardi-Lamardo E. (2026). Filtered threats: The impact of cigarette butt leachate on marine life. Mar. Pollut. Bull..

[51] Dolar A., Mušič B., Skalar T., Marolt G., Drobne D., Škapin A.S., Jemec Kokalj A. (2025). Microplastics from cigarette filters: Comparative effects on selected terrestrial and aquatic invertebrates. Environ. Pollut..

[52] Green D.S., Kregting L., Boots B. (2020). Smoked cigarette butt leachate impacts survival and behaviour of freshwater invertebrates. Environ. Pollut..

[53] Lian H., Xu C., Xu P., Tong D., Li Z., Cheng X., Zhang K., Xiang X. (2025). Cigarette butts: A hidden toxic time bomb for aquatic metabolism and behavior. Environ. Pollut..

[54] Srikanth K., Sravani S.S., Pamanji R., Shaik N., Sivan G., Raju N.V. (2025). Cigarette butts: A source of toxicity and potential disruption of *Pila virens* antioxidant mechanisms. Toxicol. Res..

[55] Olah-Kovacs B., White H.J., Tingey T., Boots B., Green D.S. (2025). The impacts of smoked cigarette butt leachate on a common freshwater gastropod, Lymnaea stagnalis. Environ. Pollut..

[56] Oliva M., De Marchi L., Cuccaro A., Pretti C. (2021). Bioassay-based ecotoxicological investigation on marine and freshwater impact of cigarette butt littering. Environ. Pollut..

[57] Mohammadi A., Bahrani F., De-la-Torre G.E., Saeedi R., Renner G., Schmidt T.C., Dobaradaran S. (2025). Cigarette filters as a major source of microfibers in aquatic environments. Sci. Rep..

[58] Bonanomi G., Iacomino G., D’Esposito D., Monti M.M., Siciliano A., Santorufo L., Spampinato M., Ruocco M., Verrillo M.V., Spaccini R. (2026). Long-term cigarette butts’ decomposition over 10 years reveals multi-stage microbial, chemical, and toxicological transformations. Environ. Pollut..

[59] Garrido Lazo R.A., Manrique Suárez R., Bravo Guerra M.F., Soto Silva C.C., Pizarro Konczak J., Ortiz Calderón C. (2024). Ecotoxicological Impact of Cigarette Butts on Coastal Ecosystems: The Case of Marbella Beach, Chile. Sustainability.

[60] Belzagui F., Buscio V., Gutiérrez-Bouzán C., Vilaseca M. (2021). Cigarette butts as a microfiber source with a microplastic level of concern. Sci. Total Environ..

[61] Yang Q., Zhong W., Jiao Y., Zhang Y., Cheng L., Ruan Y., Yang S. (2024). Littered cigarette butts in both coastal and inland cities of China: Occurrence and environmental risk assessment. Front. Mar. Sci..

[62] Venugopal P.D., Hanna S.K., Gagliano G.G., Chang H.W. (2021). No Butts on the Beach: Aquatic Toxicity of Cigarette Butt Leachate Chemicals. Tob. Regul. Sci..

[63] Nongdren L., Parveen N., Vanapalli K.R. (2026). Cradle to grave environmental analysis of cigarettes with emphasis on end-of-life management. Waste Manag..

[64] Torkashvand J., Godini K., Norouzi S., Gholami M., Yeganeh M., Farzadkia M. (2021). Effect of cigarette butt on concentration of heavy metals in landfill leachate: Health and ecological risk assessment. J. Environ. Health Sci. Eng..

[65] Piccardo M., Provenza F., Anselmi S., Broccoli A., Terlizzi A., Renzi M. (2021). Use of Sediqualsoft^®^ to Determine the Toxicity of Cigarette Butts to Marine Species: A Weather Simulation Test. J. Mar. Sci. Eng..

[66] Soleimani F., Dobaradaran S., Vazirizadeh A., Mohebbi G., Ramavandi B., De-la-Torre G.E., Nabipour I., Schmidt T.C., Novotny T.E., Maryamabadi A. (2023). Chemical contents and toxicity of cigarette butts leachates in aquatic environment: A case study from the Persian Gulf region. Chemosphere.

[67] Nongdren L., Parveen N., Vanapalli K.R. (2024). Cigarette butt littering as a potential source of water pollution and human health risk. J. Environ. Chem. Eng..

[68] Dobaradaran S., Schmidt T.C., Mutke X.A.M., De-la-Torre G.E., Telgheder U., Kerpen K., Plonowski M. (2023). Aromatic amines leachate from cigarette butts into aquatic environments: Is there risk for water organisms?. Environ. Res..

[69] Leslie H.A., Depledge M.H. (2020). Where is the evidence that human exposure to microplastics is safe?. Environ. Int..

[70] Jaishankar M., Tseten T., Anbalagan N., Mathew B.B., Beeregowda K.N. (2014). Toxicity, mechanism and health effects of some heavy metals. Interdiscip. Toxicol..

[71] Bolden A.L., Kwiatkowski C.F., Colborn T. (2015). New Look at BTEX: Are Ambient Levels a Problem?. Environ. Sci. Technol..

[72] Nitschke T., Bour A., Bergquist M., Blanchard M., Molinari F., Almroth B.C. (2023). Smokers’ behaviour and the toxicity of cigarette filters to aquatic life: A multidisciplinary study. Microplast. Nanoplast..

[73] World Health Organization (2017). Tobacco and Its Environmental Impact: An Overview.

[74] European Parliament and Council of the European Union (2019). Directive (EU) 2019/904 of 5 June 2019 on the reduction of the impact of certain plastic products on the environment. Off. J. Eur. Union.

