# Supplementary material for: Toxicants Released from Cigarette Butt and Other Tobacco and Nicotine Product Waste and Their Ecotoxicological Impact: A Systematic Review with One Health Perspectives
Source: Toxics. 2026 Sep 23;14(10):846. doi: 10.3390/toxics14100846 (PMC13709674; doi:10.3390/toxics14100846)
Supplement: Supplementary file 1 [file toxics-14-00846-s001.zip › Table S4 main findings rev 01.pdf]

Table S4. Main findings, dose–effect relationships, and study limitations of the included studies investigating the environmental and ecotoxicological impacts of cigarette butt and related tobacco product waste.

Author Year	Main findings	Dose–effect interpretation	Study limitations reported by Authors
Arfaeinia et al. 2024	- ΣPCBs ranged from <LOD–1.36 μg/L in smoked cigarette butt (SCB) leachates, <LOD–1.02 μg/L in smoked filter (SF) leachates and <LOD–0.86 μg/L in unsmoked filter (USF) leachates, with the overall pattern SCBs > SFs > USFs ($p < 0.001$). Most individual PCB congeners also differed significantly among leachate types ($p < 0.005$).<br>- PCB105 was the predominant congener in SCB and SF leachates, whereas PCB18 predominated in USF leachates.<br>- Dioxin-like PCBs (DL-PCBs) accounted for 34% of total PCBs, whereas non-DL PCBs represented 66%.<br>- TEQ values for DL-PCBs ranged from 2×10^{-6} to 1×10^{-4} ng/L.<br>- OCP concentrations ranged from <LOD–0.14 μg/L in SCB leachates and <LOD–0.10 μg/L in SF leachates; in USF leachates, only α-lindane (0.08 μg/L) was detected.<br>- α-Lindane and DDT concentrations differed significantly among leachate types ($p < 0.005$).<br>- Ecological risk assessment showed medium ecological risk for α-lindane (RQ = 0.38, 0.27 and 0.22 for SCB, SF and USF leachates, respectively) and high ecological risk for DDT in SCB (RQ = 3.33) and SF (RQ = 2.67) leachates.	Smoked cigarette butts released higher concentrations of persistent organic pollutants than filters alone. Risk characterization indicated that DDT posed a high ecological risk, whereas α-lindane posed a medium ecological risk in aquatic environments.	Mechanisms and toxicity of cigarette butt leachates in aquatic organisms remain incompletely understood.
Bakr et al. 2025	- SCB-NPs exhibited the smallest particle size (156.7 ± 5.6 nm), followed by USCB-NPs (195.7 ± 25.0 nm) and CCA-NPs (214.0 ± 12.2 nm).<br>- SCB-NPs caused the highest mortality (21.66%; n = 13), followed by USCB-NPs (16.6%; n = 10) and CCA-NPs (13.3%; n = 8), compared with the control (6.66%; n = 4) ($P < 0.0001$, $P < 0.001$ and $P < 0.01$, respectively).<br>- Earthworm growth decreased by 8.44% following SCB-NPs exposure, compared with 5.01% for USCB-NPs and 3.65% for CCA-NPs, whereas the control group showed an 11.17% increase in growth ($P < 0.0001$).<br>- All exposure groups induced significant oxidative stress and antioxidant enzyme inhibition; however, biochemical alterations were consistently greater in earthworms exposed to SCB-NPs than to USCB-NPs or CCA-NPs.<br>- DNA damage assessed by the Comet assay was significantly greater following SCB-NPs exposure than after exposure to USCB-NPs or CCA-NPs.	Cellulose acetate nanoplastics derived from smoked cigarette butts produced greater ecotoxicological effects than nanoplastics derived from unsmoked cigarette butts or commercial cellulose acetate, indicating that combustion-derived residues substantially increase the toxicity of cigarette butt-derived nanoplastics to soil organisms.	- FTIR confirmed nanoparticle uptake but did not allow quantitative assessment of internalization due to the lack of ICP analysis.<br>- Combustion-derived contaminants associated with smoked cigarette butts were not chemically characterized or directly measured.<br>- Long-term ecological consequences of cellulose acetate nanoplastic exposure were not investigated.

	- Overall toxicity followed the pattern: SCB-NPs > USCB-NPs > CCA-NPs.		
Banaee et al. 2024	- No mortality was observed during the 21-day exposure.<br>- LDH increased from 213.5 ± 12.9 to 313.2 ± 4.3 U/L, while AST increased from 70.6 ± 1.0 to 88.2 ± 2.3 U/L at 2.0 µL/L.<br>- ALT increased significantly only at 1.5 and 2.0 µL/L, reaching 13.9 ± 0.8 and 17.9 ± 0.8 U/L, respectively, compared with 11.5 ± 0.4 U/L in controls.<br>- GGT increased from 16.5 ± 1.1 to 24.5 ± 0.4 U/L, whereas ALP decreased from 231.4 ± 13.6 to 172.0 ± 5.8 U/L.<br>- Plasma glucose increased from 49.53 ± 0.40 to 80.81 ± 2.30 mg/dL, cholesterol from 53.8 ± 3.3 to 177.5 ± 6.2 mg/dL, triglycerides from 59.1 ± 5.1 to 157.2 ± 8.9 mg/dL, and creatinine from 0.17 ± 0.01 to 0.50 ± 0.01 mg/dL.<br>- Total protein decreased from 4.63 ± 0.64 to 3.10 ± 0.01 g/dL, albumin from 2.45 ± 0.04 to 2.05 ± 0.04 g/dL, and globulin from 2.18 ± 0.64 to 1.05 ± 0.04 g/dL.<br>- CAT, SOD and GPx activities and MDA levels increased significantly, whereas GSH content and total antioxidant capacity decreased, with the greatest alterations observed at 2.0 µL/L.<br>- Raman spectroscopy revealed concentration-dependent alterations in liver and muscle biochemical profiles, including reduced phospholipids, lipids, proteins, nucleic acids and carbonyl-containing biomolecules, together with collagen damage and altered phenylalanine-related signals.	Sublethal exposure produced a generally concentration-dependent pattern of hepatotoxicity, renal dysfunction, metabolic disturbance and oxidative stress. Effects were detectable at concentrations substantially below the previously reported 96-h LC_{50}, with the most pronounced biochemical and molecular alterations occurring at 1.5-2.0 µL/L.	Not reported
Belzagui et al. 2021	- Each smoked filter released approximately 450 microfibres on the first day and subsequently about 100 microfibres per day during the first 2 weeks; most fibres were <0.2 mm in length.<br>- The estimated global release was approximately 4×10^{18} microfibres per year, corresponding to roughly 0.3 million tonnes annually entering aquatic environments.<br>- After 48 h, the condition containing leachate plus microfibres produced an EC50 of 0.017 SF/L, compared with 0.067 SF/L for leachate alone, indicating an approximately fourfold increase in toxicity attributable to the presence of microfibres.<br>- At lower concentrations, the toxic effect associated with microfibres was estimated to be 5- to 10-fold greater than that of leachate alone.<br>- The estimated concentration causing 100% immobilisation was 0.620 SF/L for leachate plus microfibres and 0.888 SF/Lfor leachate alone.<br>- A statistically significant difference between treatments was observed at concentrations <0.1 SF/L ($p = 0.038$).	The presence of cellulose acetate microfibres substantially increased the acute toxicity of smoked-filter leachate, particularly at low and environmentally more plausible concentrations. Toxicity was attributable to both chemical exposure and fibre-related physical effects, including entanglement, while the very low degradation rate indicated the potential for prolonged environmental persistence and chronic exposure.	Not reported

	- External entanglement of Daphnia magna by microfibres was observed, reducing mobility and accelerating mortality, particularly at concentrations <0.1 SF/L.<br>- FTIR spectra retained approximately 95% similarity to cellulose acetate after 1 month in distilled or synthetic seawater and after 18 months in freshwater and natural sunlight, indicating no relevant chemical degradation.<br>- In the modified Fenton-reaction test, cellulose acetate filter degradation reached a plateau at <10% mass loss, even after prolonged treatment.		
Bonanomi et al. 2026	- CB decomposition followed a four-stage pattern, with an initial 15–20% mass loss during the first month, approximately 30–35% cumulative mass loss after 2 years, and final mass losses after 10 years of 52% (laboratory without soil), 66% (field sand dune), 76% (laboratory sand dune soil) and 84% (laboratory grassland soil).<br>- The C/N ratio decreased from 192.4 in fresh cigarette butts to 16.9 after 10 years in laboratory grassland soil, indicating substantial nitrogen enrichment during decomposition.<br>- ^{13}C CPMAS NMR demonstrated progressive deacetylation and degradation of cellulose acetate, particularly under nitrogen-rich grassland soil conditions<br>- Microbial diversity declined between 5 and 10 years, with clear shifts in bacterial and fungal community composition across decomposition environments.<br>- Fresh smoked and unsmoked cigarette butts exhibited the highest ecotoxicity, whereas toxicity generally declined during decomposition; however, 10-year-old cigarette butts still produced measurable biological effects.<br>- Daphnia magna remained one of the most sensitive bioindicators, with toxicity values of 0–22% in 10-year-old cigarette butt leachates depending on the decomposition condition.<br>- PTR-ToF-MS identified 336 VOCs, of which 325 differed significantly among decomposition stages. Nicotine and acrylonitrile were detected exclusively in freshly smoked cigarette butts, whereas emissions of benzene, formaldehyde and toluene decreased markedly after long-term decomposition.	Although cigarette-butt toxicity decreased during decomposition, aged cigarette butts remained ecologically relevant after 10 years, indicating that cellulose acetate persistence and long-term chemical transformations can sustain chronic environmental hazards despite substantial mass loss.	Not reported.
Caridi et al. 2020	- GC-MS analysis showed a nicotine concentration of 62.5 mg/L in the stock cigarette-butt infusion.<br>- LC_{50} (48 h) values were 1.9 cigarette butts/L for Rosalina globularis, 5.6 cigarette butts/L for Textularia agglutinansand 7.2 cigarette butts/L for Quinqueloculina spp., indicating that R. globularis was the most sensitive species.	Marked concentration-related toxicity with species-specific sensitivity. R. globularis showed the lowest 48-h LC50 (1.9 CB/L), while calcareous taxa exhibited reduced viability and shell decalcification from 4 CB/L onward. T. agglutinans was comparatively more tolerant and showed partial recovery at higher concentrations.	Not reported

	- Both calcareous genera exhibited marked reductions in viability from 4 cigarette butts/L onwards, whereas T. agglutinans partially recovered at 8 and 16 cigarette butts/L.<br>- Shell decalcification occurred in all three species at 4–16 cigarette butts/L. In Quinqueloculina spp., complete dissolution of the imperforate test and cytoplasmic disorganization were observed at the highest concentrations.<br>- FTIR analysis showed pronounced biochemical alterations in R. globularis and Quinqueloculina spp., whereas T. agglutinans exhibited comparatively limited metabolic changes.		
Cho et al. 2025	- GSF microplastics were significantly smaller than CAF microplastics (11.69 ± 9.90 vs 19.04 ± 16.40 µm), with most particles measuring <70 µm, facilitating ingestion by Daphnia magna.<br>- Nicotine was detected in GSF (3.58 ± 0.20 µg/mg) but was not detected in CAF, while LC-HRMS identified several additional organic compounds released from smoked cigarette filter-derived microplastics.<br>- Acute exposure caused concentration-dependent immobilisation and mortality in all exposure groups, with GSF consistently producing greater toxicity than both CAF and LSF at equivalent concentrations.<br>- During the 21-day chronic exposure, GSF produced the greatest reduction in reproductive performance, including fewer neonates, delayed first brood and smaller offspring, whereas CAF induced milder reproductive effects.<br>- Both GSF and CAF significantly altered oxidative stress, detoxification, endocrine- and moulting-related gene expression; however, molecular alterations were consistently more pronounced in GSF-exposed organisms than in CAF-exposed organisms.<br>- Overall, cigarette filter-derived microplastics produced greater long-term toxicity than cigarette filter leachates alone, indicating that the particulate fraction substantially contributes to the adverse effects of cigarette filter waste in freshwater ecosystems.	Cigarette filter-derived microplastics induced both particle- and chemical-mediated toxicity in *Daphnia magna*. Smoked cigarette filter microplastics produced greater acute and chronic toxicity than non-smoked filter microplastics, resulting in impaired reproduction, oxidative stress and altered expression of detoxification, endocrine- and moulting-related genes. These findings indicate that cigarette filter-derived microplastics can act as long-term vectors of toxic chemicals in freshwater ecosystems.	Not reported
Couto et al. 2025	- At 25 °C, the 48-h LC_{50} was 3.98 CBs/L for SCB elutriates and 0.94 CBs/L for nSCB elutriates, indicating that nSCB elutriates were more acutely toxic than SCB elutriates under current temperature conditions.<br>- Increasing the temperature from 25 to 30 °C markedly enhanced the acute toxicity of SCB elutriates, reducing the 48-h LC_{50} from 3.98 to 1.26 CBs/L, whereas only minor changes were observed for nSCB elutriates.<br>- SCB elutriates caused significant reductions in body growth, altered metabolic activity and induced oxidative stress, with these effects becoming more pronounced at 30 °C.	Cigarette butt elutriates produced marked acute and sub-chronic toxicity in *Artemia franciscana*. Increased temperature intensified several toxic effects, including neurotoxicity, oxidative stress and growth impairment, indicating that climate change may exacerbate the ecological impacts of cigarette butt pollution in marine ecosystems.	Not reported

	- nSCB elutriates also significantly affected antioxidant and detoxification biomarkers, although the biochemical response differed from that observed for SCB elutriates, indicating distinct toxicological profiles.<br>- Exposure to both SCB and nSCB elutriates significantly inhibited AChE activity, increased lipid peroxidation and disrupted glutathione homeostasis, with the strongest oxidative damage generally occurring under the warming scenario (30 °C).<br>- Overall, elevated temperature amplified the toxic effects of cigarette butt elutriates, particularly those derived from smoked cigarette butts, suggesting that climate warming may increase the ecological hazard posed by cigarette butt pollution in marine environments.		
Ferreira-Griz et al. 2026	- Cigarette butt leachate contained Cu (53 µg/L), Fe (45 µg/L), Zn (60 µg/L) and a total PAH concentration of 2149 ng/L, predominantly represented by low-molecular-weight PAHs, particularly naphthalene.<br>- Lysosomal membrane stability (NRRT) was significantly impaired at almost all tested leachate concentrations after both 24 and 120 h of exposure, with marked time-dependent effects at the lowest concentrations.<br>- Sublethal cellular effects were detected at concentrations as low as 0.01% CB leachate, corresponding to approximately one cigarette butt per 2500 L of water.<br>- Despite limited bioaccumulation of trace metals, exposed mussels accumulated naphthalene and phenanthrene in their tissues following exposure to cigarette butt leachate.<br>- No substantial mortality occurred during the experiments, indicating that cigarette butt leachate primarily induced sublethal cellular toxicity rather than acute lethal effects under the tested conditions.<br>- Overall, cigarette butt leachate induced early cellular stress and PAH bioaccumulation in *Mytella charruana*, demonstrating that environmentally realistic concentrations may adversely affect marine filter-feeding organisms.	Cigarette butt leachate induced concentration- and time-dependent sublethal toxicity in *Mytella charruana*. Cellular stress occurred even at environmentally realistic concentrations, while PAH bioaccumulation was more evident than trace metal accumulation, suggesting that organic contaminants play a major role in the observed toxicity.	Approximate environmental exposure estimates based on laboratory leaching assumptions; PAHs were characterized only in leachates and not in cigarette butts; inter-study comparisons were limited by differences in cigarette brands and leaching protocols

Dobaradaran et al. 2020	- ΣPAH concentrations increased with contact time, ranging from 3.9 to 5.7 µg/L in deionized water, 3.3 to 5.5 µg/L in tap water and 3.0 to 5.0 µg/L in river water between 4 h and 21 days.<br>- No substantial differences in ΣPAH concentrations were observed among the three water matrices at the same contact time.<br>- Naphthalene and fluorene were the predominant PAHs at all sampling times, whereas indeno(1,2,3-cd)pyrene and dibenz(a,h)anthracene remained below the limit of detection throughout the experiment.<br>- PAH concentrations increased progressively from 4 h to 21 days, indicating continuous release of contaminants from cigarette butts during prolonged water contact.<br>- Tap and river water samples amended with sodium azide contained significantly higher ΣPAH concentrations than untreated samples, suggesting that microbial activity contributed to PAH attenuation.<br>- Several detected PAHs exceeded Water Framework Directive (WFD) environmental quality standards, indicating the potential ecological relevance of cigarette butt-derived PAH contamination.	Cigarette butts continuously released PAHs into freshwater over time, with prolonged water contact increasing contaminant concentrations irrespective of water type. The exceedance of WFD environmental quality standards suggests that cigarette butt-derived PAHs may represent a relevant source of chemical contamination in aquatic environments.	Laboratory batch experiments may not fully represent environmental conditions; global PAH release estimates were based on a maximum contact time of 21 days, and longer-term leaching requires further investigation.
Dobaradaran et al. 2021	- All BTEX compounds were detected in both distilled water and river water after only 15 min of contact, indicating rapid leaching from smoked cigarette butts.<br>- Toluene was the predominant compound, reaching 0.54–0.90 mg/L in distilled water and 0.39–0.74 mg/L in river water, whereas o-xylene showed the lowest concentrations (0.11–0.19 mg/L and 0.09–0.13 mg/L, respectively).<br>- BTEX concentrations reached equilibrium within approximately 15 min, with no significant increase after longer contact times up to 24 h.<br>- No significant differences in BTEX concentrations were observed between distilled water and river water, indicating that water matrix had little influence on BTEX release.<br>- Delaying immersion after smoking significantly reduced BTEX leaching, with the greatest decreases occurring during the first hours after cigarette extinction, reflecting rapid volatilisation of these compounds.<br>- Partitioning analysis indicated that BTEX compounds have a high tendency to leach from cigarette butts into water and subsequently volatilise into the atmosphere because of their relatively low log KOC/water values.	BTEX compounds were rapidly released from smoked cigarette butts, with most leaching occurring within the first 15 min after contact with water. The rapid equilibrium and subsequent decline associated with delayed immersion indicate that cigarette butts represent an immediate but transient source of BTEX contamination in aquatic environments.	As an initial study, several factors potentially influencing BTEX leaching (e.g., water quality, flow and turbulence, cigarette brand, sorption/desorption mechanisms, water temperature and burning style) were not investigated.

Dobaradaran et al. 2022	- The mean ΣPAAs concentration was significantly higher in freshly smoked cigarette butts (3.43 μg/g) than in aged cigarette butts (2.12 μg/g) and unsmoked cigarettes (0.28 μg/g), following the pattern: freshly smoked CBs > aged CBs > unsmoked cigarettes ($P < 0.05$).<br>- Freshly smoked cigarette butts showed marked increases in ΣPAAs (12.26-fold), Σtoluidines (28.84-fold), 2,6-dimethylaniline (10.41-fold), o-anisidine (8.46-fold), 2-naphthylamine (4.78-fold) and aniline (4.05-fold) compared with unsmoked cigarettes.<br>- o-Anisidine was the predominant aromatic amine in all three sample categories.<br>- Most aromatic amines decreased substantially in aged cigarette butts compared with freshly smoked cigarette butts, whereas o-anisidine remained relatively persistent after environmental ageing.<br>- The results indicate that smoking substantially increases aromatic amine concentrations in cigarette butts and that aged cigarette butts remain an important reservoir of these contaminants in the environment.	Smoking markedly increased the accumulation of aromatic amines in cigarette butts. Although environmental ageing reduced the concentrations of most compounds, aged cigarette butts remained a significant reservoir of aromatic amines, supporting their potential to act as a long-term source of environmental contamination.	Not reported
Dobaradaran et al. 2023	- ΣAAs ranged from 569 ng/L in distilled water to 556 ng/L in river water, with no significant differences between the two water matrices ($P > 0.05$).<br>- Aniline was the predominant aromatic amine, followed by 1-naphthylamine, 2-naphthylamine, 2,6-dimethylaniline, toluidines, o-anisidine and aminobiphenyls. Individual AA concentrations ranged from 0.2 to 566 ng/L in distilled water and from 0.2 to 596 ng/L in river water.<br>- AA concentrations increased progressively throughout the 30-day exposure period, indicating continuous release from cigarette butts into aquatic environments.<br>- Partitioning analysis showed relatively low log KOC/water values and moderate water–air partitioning, indicating that aromatic amines are readily released into the aqueous phase after cigarette butt disposal.<br>- Ecological risk assessment identified ΣAAs, aniline, p-toluidine, o-toluidine, 2-naphthylamine and aminobiphenyls as presenting a medium ecological risk for sensitive crustaceans and fish.<br>- No significant differences in leaching behaviour were observed between distilled water and river water, suggesting that water chemistry had limited influence on aromatic amine release under the tested conditions.	Cigarette butts continuously released aromatic amines into aquatic environments over time. Although concentrations were similar in distilled and river water, ecological risk assessment indicated that several aromatic amines may pose a medium risk to sensitive aquatic organisms, supporting cigarette butts as a relevant source of chemical contamination.	Limited ecotoxicological data prevented RQ estimation for 1-naphthylamine; additional aromatic amines not evaluated in this study may also contribute to ecological risk.
Dobaradaran et al. 2024a	- Remaining tobacco plus ash released the highest nicotine concentrations (5.73–17.34 mg/g), followed by freshly smoked cigarette butts (0.36–8.60 mg/g), aged cigarette butts (0.31–4.12 mg/g) and filter plus paper (0.17–2.79 mg/g).	Nicotine was continuously released from all cigarette butt components, with the remaining tobacco plus ash representing the primary source of contamination. Although environmental ageing reduced nicotine release, aged cigarette butts	RQ assessment was based only on nicotine concentrations and did not account for other cigarette butt toxicants; no dilution scenarios representative of real environmental conditions were considered.

	- Nicotine release increased with exposure time in all groups, with the greatest amounts consistently released from the remaining tobacco plus ash fraction throughout the experiment.<br>- Environmental ageing substantially reduced nicotine release compared with freshly smoked cigarette butts; however, aged cigarette butts still released considerable amounts of nicotine into water.<br>- Based on worldwide cigarette litter estimates, freshly smoked cigarette butts were estimated to release approximately 380–7065 tonnes of nicotine annually into aquatic environments.<br>- Ecological risk assessment indicated that nicotine leachates from all cigarette butt types and remaining components posed a high ecological risk for fish, cladocerans, algae and Daphnia magna.<br>- Overall nicotine release followed the pattern: remaining tobacco plus ash > freshly smoked cigarette butts > aged cigarette butts > filter plus paper.	remained an important source of nicotine pollution, and all leachates were predicted to pose a high ecological risk to aquatic organisms.	
Dobaradaran et al. 2024b	- Filter plus paper released the highest concentrations of phenolic compounds, followed by freshly smoked cigarette butts, remaining tobacco plus ash and aged cigarette butts.<br>- Mean leachate concentrations ranged from 0.43–639.56 µg/L per cigarette butt for phenol, 0.77–58.61 µg/L for o-cresol and 0.25–16.58 µg/L for 2,4-dimethylphenol.<br>- Phenol was the predominant phenolic compound in all cigarette butt types, whereas pentachlorophenol and 2,3,4,6-tetrachlorophenol were not detected at any sampling time.<br>- Phenolic compound concentrations increased with exposure time in all cigarette butt types, indicating continuous release into water during prolonged immersion.<br>- Ecological risk assessment indicated that leachates from all cigarette butt types posed a high ecological risk for algae, Daphnia magna and fish.<br>- Overall phenolic compound release followed the pattern: filter plus paper > freshly smoked cigarette butts > remaining tobacco plus ash > aged cigarette butts.	All cigarette butt components continuously released phenolic compounds into water, with the filter plus paper fraction representing the major source. Despite lower releases from aged cigarette butts, all cigarette butt types were predicted to pose a high ecological risk to sensitive aquatic organisms.	Only three phenolic compounds were investigated.

Dolar et al. 2025	- SCF-MPs were significantly smaller than UCF-MPs (9.06 ± 4.10 µm vs 12.71 ± 6.82 µm) and contained nicotine together with a larger variety of trace organic compounds, whereas both particle types contained triacetin and potentially toxic metals.<br>- Neither SCF-MPs nor UCF-MPs affected the survival of the terrestrial invertebrates Porcellio scaber and Tenebrio molitor; however, both induced significant physiological responses, including alterations in total haemocyte count, AChE, GST and ETS activities, with generally stronger effects following SCF-MP exposure.<br>- SCF-MPs significantly reduced the growth of Tenebrio molitor from Day 7 onward at 0.5–1.5% soil concentrations, whereas UCF-MPs produced delayed but significant growth inhibition, mainly after 21–28 days.<br>- Exposure to 100 mg/L SCF-MPs significantly reduced Daphnia magna survival by 20% after 48 h and by 90% after 96 h, whereas UCF-MPs did not significantly affect survival.<br>- Both SCF-MPs and UCF-MPs significantly reduced the survival of Brachionus calyciflorus at 10 mg/L after 24 h, while only SCF-MPs caused a further significant reduction at 100 mg/L.<br>- Overall, SCF-MPs consistently produced more pronounced biological effects than UCF-MPs across terrestrial and aquatic organisms, indicating that smoking-derived contaminants increase the ecotoxicological hazard of cigarette filter microplastics.	Microplastics derived from smoked cigarette filters produced stronger concentration- and time-dependent ecotoxicological effects than those derived from unsmoked filters. The additional contaminants retained during smoking (e.g., nicotine and trace organic compounds) appear to enhance the toxicity of cigarette filter microplastics toward both terrestrial and aquatic invertebrates.	Not reported
dos Santos et al. 2024	Cigarette butt leachate significantly affected freshwater phytoplankton communities. Total cell density decreased progressively with increasing leachate concentration, with significant differences between all treatment combinations. Species richness also declined from a mean of 20 species in the control to 16, 15, and 11 species at 1, 5, and 10 CBs L^{-1}, respectively; the control had significantly higher richness than all exposed treatments, while the 1 and 5 CBs L^{-1} treatments had significantly higher richness than 10 CBs L^{-1}. Community composition differed significantly among all treatments and became increasingly dissimilar from the control as leachate concentration increased. Cryptophyceae decreased with increasing exposure and were absent at 10 CBs L^{-1}, whereas Mediophyceae increased, reaching 34% at the highest concentration; dominant species also shifted across treatments. In contrast, chlorophyll-a concentrations did not differ significantly among treatments ($P = 0.08$), despite being numerically higher in the control.	Clear concentration-dependent adverse effect for cell density and species richness, with progressive alteration of community composition; no significant concentration-response for chlorophyll-a. The strongest biological effects occurred at 10 CBs L^{-1}.	Not reported

El Hadri et al. 2021	Both cigarette filter types showed high sorption capacity for all nanoplastics tested. For spherical PSLs, the residual fraction decreased rapidly and equilibrium was reached within approximately 0.5–1 h; although retention differed among particle sizes (approximately 0.01 residual fraction for 50-nm PSLs versus 0.1 for 500-nm PSLs), no correlation between PSL size and sorption kinetics was observed. Fragmented NPTf showed residual fractions of 0.12 and 0.15 for R and G filters, respectively, but required approximately 4 h to reach equilibrium compared with about 1 h for spherical PSLs, demonstrating an important influence of particle morphology. Sorption was slightly higher for the R filter, particularly for 50-nm PSLs, but filter type contributed less to sorption behaviour than nanoplastic size and morphology. KSW values ranged from approximately 10^2 to 10^4, with the highest value for 50-nm PSLs with the R filter (24,000), while Rs values for fragmented NPTf were approximately one order of magnitude lower than those for spherical PSLs. KSW values in deionized water were approximately 1.8-fold higher than in freshwater, indicating a relatively limited influence of freshwater salinity. Qualitatively, both unused and used cigarette butts rapidly removed blue 200-nm PSLs from water.	*Not applicable.*	Quantitative experiments could not be performed with used cigarette butts because they released organic matter, including a nanoparticulate fraction, that strongly interfered with AF4 analysis.
Garshasbi et al. 2026	A total of 128 macroplastic items were recorded, with significant spatial differences among stations ($\chi^2 = 34.71$, df = 6, $p < 0.001$); Khajeh-ha Street had the highest macroplastic abundance (33 items), followed by Towhid Street (32), whereas Salman Farsi Street had the lowest (5). Cigarette butts were the most frequently observed waste item overall (39; 30%), while plastic cups (18; 14%) and bags (13; 10%) were the most abundant macroplastic items. Most macroplastics were white (73%), 1–5 cm in size (49.21%), and derived from household waste (55.46%); soft plastics represented 89.84%of collected macroplastics. Among identified polymers, PET showed the highest abundance (17.18%) and PS the lowest (4.68%), with polymer distribution varying according to urban land use. CEI values exceeded 20 at every station, classifying all sites as extremely dirty, with significant differences among stations ($F = 5.93$, $p < 0.01$). Cigarette butt abundance also differed significantly among stations ($\chi^2 = 15.24$, df = 6, $p < 0.05$), with Towhid Street showing the highest proportion (35.89%) and Salman Farsi Street the lowest (2.56%). CBPI ranged from 20.0 to 279.9 (mean 111.43)and differed significantly among sites ($F = 8.77$, $p < 0.01$); all seven stations were classified as extremely dirty. Mean cigarette butt density was 3.71 items/m². Overall, the results demonstrated marked spatial heterogeneity and severe macroplastic and cigarette butt contamination throughout Bushehr's stormwater network.	Not applicable	Sampling was conducted only during the regional wet season; therefore, the findings represent peak accumulation conditions and may not reflect dry-season conditions.
Green et al. 2020	Leachate toxicity was driven primarily by concentration rather than filter material, with no significant difference in adult survival between leachates derived from biodegradable cellulose and conventional cellulose acetate butts. At 5 butts/L, most adult *D. polymorpha, P. nigra, P. planorbis* and *B. tentaculata* died within approximately 72 h, and mortality was significantly greater than at 1, 0.2 butts/L or control conditions (all relevant comparisons $P < 0.001$). Juvenile *B. tentaculata* were particularly sensitive: at 5 butts/L, <20% survived after only 24 h, irrespective of filter type. At 1 butt/L, cellulose acetate leachate initially produced lower juvenile survival at 48–72 h than cellulose leachate, but this difference disappeared with longer exposure; by 120 h, survival was ~100% at 0.2 butts/L, ~30% at 1 butt/L and <5% at	Clear concentration-dependent adverse effect. Mortality and juvenile survival worsened progressively with increasing leachate concentration, most clearly demonstrated at 120 h by juvenile survival of 100% at 0.2, ~30% at 1 and <5% at 5 butts/L. Sublethal behavioural impairment was already evident at 1 butt/L. The dose-related effect was	Not reported

	5 butts/L, with all concentration comparisons significant. Adults showed little mortality at 1 butt/L and no mortality in controls or at 0.2 butts/L, whereas juvenile survival at 1 butt/L was only ~30% after 120 h compared with approximately 80% for adults, indicating greater sensitivity of the juvenile life stage. Among surviving organisms, behaviour did not differ significantly according to filter material (P = 0.458), but 1 butt/L significantly altered behaviour compared with both controls and 0.2 butts/L, with animals showing less movement/filtering and more closed/inactive states. Overall, biodegradable cellulose-filter butts were not less toxic than conventional cellulose acetate-filter butts under the tested conditions.	independent of whether filters were cellulose or cellulose acetate.	
Green et al. 2023	The different smoking-related waste products produced contrasting effects on *L. minor*. Exposure to 1 cigarette butt/L stimulated plant growth: biomass was approximately 2.1-fold higher than controls, root growth increased by approximately 515% compared with 180% in controls, and final root length was approximately 2.9-fold greater than controls. Cigarette-butt exposure also increased frond number by 133 ± 10%, resulting in significantly more fronds than controls (1.23-fold; P = 0.031). In contrast, closed e-cigarettes, opened e-cigarettes and isolated e-liquid significantly reduced frond production compared with controls, with final frond numbers approximately 0.3-, 0.4-, and 0.4-fold lower, respectively. Cigarette-butt exposure significantly increased chlorophyll-a to approximately 1.5-fold the control level (P = 0.018) and total chlorophyll from 625 ± 58 µg/g dry biomass in controls to 920 ± 59 µg/g, whereas chlorophyll-b was not significantly affected. Overall, cigarette butts stimulated *L. minor* growth, whereas used disposable e-cigarettes—particularly opened devices—and isolated e-liquid inhibited growth. The similar responses to opened e-cigarettes and isolated e-liquid indicated that residual e-liquid was the principal component associated with the inhibitory effects observed for disposable e-cigarettes.	Not assessable (single concentration tested for each exposure type).	Short-term exposure; lack of environmental baseline data on litter densities; removal of e-cigarette batteries.
Guttmann et al. 2024	*CB leachate significantly altered both cyanobacterial growth and host–parasite interactions. In uninfected cultures, cyanobacterial growth was unaffected at 0.2 CB L^{-1} but inhibited at 2 and 10 CB L^{-1} compared with controls. In infected cultures, the opposite pattern emerged at higher exposure: cyanobacterial growth increased because CB leachate suppressed the chytrid parasite, with biomass at the highest concentration reaching levels comparable to uninfected controls. Infection prevalence decreased at all tested concentrations and was completely suppressed at 2 and 10 CB L^{-1}, whereas infection intensity at 0.2 CB L^{-1} did not differ from the control. Chemical analysis confirmed that CBs released a complex mixture of inorganic and organic constituents, including elevated macroelements and trace elements, nicotine, and cotinine. Overall, CB leachate directly inhibited cyanobacterial growth in the absence of the parasite but indirectly favoured cyanobacterial growth in infected cultures by suppressing chytrid infection.*	A concentration-dependent effect was evident but differed according to the host–parasite condition. Increasing CB leachate concentrations inhibited cyanobacterial growth in uninfected cultures, whereas increasing concentrations progressively suppressed chytrid infection, with complete inhibition at 2 and 10 CB L^{-1}. This parasite suppression indirectly favoured cyanobacterial growth in infected cultures at higher CB concentrations.	The effects of individual chemicals and their potential interactions within the complex CB-leachate mixture were not separately assessed.
Jakimiuk et al. 2022	*CB effects depended on both concentration and plant species, with evidence of stimulation at low exposure and inhibition at higher exposure. In S. alba, low exposure to the 3-CB leachate, particularly after 7 days of leaching, increased RSG, RRG and GI above control values, consistent with a hormetic response. In contrast, the 10-CB treatment markedly inhibited germination, with SG declining to approximately 53% after 24 h, 47% after 7 days and 13% after 3 weeks, showing stronger inhibition with increasing CB concentration and leaching time. Root growth*	Biphasic concentration-dependent effect (hormesis at low exposure and inhibition at higher exposure), with species-dependent sensitivity. Low CB concentrations occasionally stimulated germination and root growth, whereas increasing CB concentration	Possible Petri-dish contamination affecting one anomalous result.

	of S. alba was also strongly inhibited as CB concentration increased: after the longest leaching period, mean root length decreased from approximately 30 mm in controls to 17 mm at 3 CBs, 4 mm at 5 CBs and <2 mm at 10 CBs. H. vulgare was substantially more tolerant. Its germination was less affected by increasing CB concentration, although RSG, RRG and GI generally declined with greater CB concentration and longer leaching time; a slight stimulatory effect was observed at the lowest exposure, including an RRG of about 108% for 3 CBs after 24 h. Root-length inhibition was also less pronounced in H. vulgare: after 24-h leaching, mean root length remained around 30 mm for 0, 3 and 5 CBs and decreased to approximately 19 mm at 10 CBs, whereas S. alba declined from 14 mm in controls to 4, 5 and 3 mm at 3, 5 and 10 CBs, respectively. Two-way ANOVA showed that after 7 days of CB leaching both plant species and CB exposure significantly affected root length ($p < 0.05$); CB concentration was also significant after 3 weeks, whereas no significant species × CB interaction was demonstrated. Overall, high CB concentrations adversely affected seed germination and radicle growth, while H. vulgare exhibited greater resistance than S. alba.	progressively reduced SG, RRG, GI and root length, particularly in *S. alba*. Longer leaching time further increased the inhibitory effect on seed germination, although the authors report that leaching duration did not significantly affect root length overall.	
Kim et al. 2023	Exposure to CB-derived microfibers did not significantly affect shrimp wet weight or body length, but increased cumulative molting, indicating alteration of the normal molting cycle. Structural examination of the carapace showed separation/gaps between cuticular layers and a reduction in endocuticle thickness. The endocuticle-to-exocuticle thickness ratio in the head carapace was significantly reduced ($p < 0.014$), and total cuticle thickness was significantly lower in exposed shrimp ($p < 0.001$). Although carapace calcium content and the Ca:Mg ratio tended to decrease following exposure, these differences were not statistically significant ($p = 0.382$).<br>CB-MF exposure also altered pigmentation. Exposed shrimp showed significantly paler body coloration after 28 days ($p < 0.004$) and lower chromatophore pigmentation; the reported total CI was 3.61 ± 1.19 in controls versus 2.26 ± 1.27 in exposed shrimp. Histological examination additionally demonstrated gill damage, including vacuole formation, edema of the gill lamellae and decreased tissue density. The authors interpret this damage as potentially impairing calcium uptake through respiration, thereby contributing to defective exoskeleton hardening. Overall, chronic CB-MF exposure increased molting activity and was associated with thinner, less pigmented carapaces and gill damage, despite the absence of detectable effects on somatic growth.	Not assessable (single concentration tested)	Inability to distinguish physical microfiber effects from effects of chemicals associated with smoked CB microfibers. The authors explicitly state that shrimp were exposed only to microfibers derived from smoked cigarettes; consequently, the study cannot determine whether the observed molting and exoskeletal alterations resulted from the physical stress of the microfibers themselves or the chemical effects of contaminants associated with smoked CBs.
Kouhi et al. 2025	The estimated abundance of CBs differed substantially according to sampling method. The FAZ approach yielded an average of 6.02 CBs/m², compared with 0.95 CBs/m² for the circular approach. When individual methods were considered, mean densities were 1.2875 ± 0.4712, 0.7112 ± 0.5212 and 0.864 ± 0.7500 CBs/m² for the 5-, 10-, and 15-m circular areas, respectively, and 2.7667 ± 1.5916 CBs/m² for the FAZ method; the overall average was 1.4028 ± 1.2328 CBs/m². The three circular methods did not differ significantly from one another, whereas each differed significantly from the FAZ method ($P < 0.05$), indicating that sampling geometry/location substantially influenced estimated CB abundance. The meta-analysis retained 4 of 17 identified studies and provided concentration estimates for 14 metals associated with discarded CBs. Among these, Mn had the highest pooled	Not applicable	The Flood Accumulation Zone method may produce biased and potentially

	concentration (36.4 µg/g) and Hg the lowest (0.05 µg/g). Estimated metal loads per unit beach area were consistently higher when calculated from the FAZ-derived CB abundance than from the circular methods; for example, estimated Mn emissions ranged from 9.43–34.58 µg/m² for the three circular methods versus 159.07 µg/m² for FAZ. The authors concluded that circular sampling is more appropriate for estimating beach CB abundance because it covers a larger area and is less affected by localized accumulation caused by waves and wind, whereas cigarette waste represents a potential point source of metal contamination in coastal environments		unrealistic estimates because the rectangular sampling area can coincide with zones where CBs accumulate because of wind and coastal-wave action; wind direction and tourist density should therefore be considered during sampling. The authors also acknowledge the advantage of FAZ sampling in allowing CB recovery from deeper beach-sand layers.
Lazo et al. 2024	CB-containing treatments produced marked chemical and phytotoxic effects compared with sand alone. Leachate pH progressively increased in the CB-only and CB + sand scenarios, whereas it remained comparatively stable in the sand-only treatment. Cellulose acetate showed minimal chemical modification throughout the experiment, indicating persistence of the filter material. Fe concentrations were highest in sand-only leachates and substantially lower in CB-containing treatments, suggesting adsorption of Fe by CB material; Zn remained low in all treatments and Cu was not detected. CB leachates strongly inhibited seed germination and seedling growth, with the CB-only treatment producing the greatest phytotoxicity and the CB + sand treatment showing intermediate effects, indicating partial mitigation by sand. *L. sativa* was more sensitive to CB leachates than *L. perenne*, showing greater inhibition of germination and growth.	No conventional concentration-based dose–effect relationship was assessed	The study used deionized water to isolate the toxicological effects of CB components, and therefore did not assess the influence of seawater salinity; the authors explicitly indicate that future studies should investigate whether salinity modifies the observed effects. Further chemical analysis was also considered necessary to determine whether sand provides a true buffering effect on toxicity beyond its apparent filtration capacity.
Lian et al. 2025	CB exposure significantly impaired feeding and swimming behavior in *B. plicatilis* and induced oxidative stress, lipid and energy metabolism disruption, and mitochondrial dysfunction. Toxicity generally increased with CB concentration, although some metabolic endpoints showed biphasic responses, with stimulation at low exposure followed by inhibition at higher concentrations. SCB generally produced the strongest effects compared with SF and USF, indicating an important contribution of residual tobacco and smoke-derived contaminants. CB fibers and leachates showed different toxicity patterns, with fiber-associated effects being more persistent and leachate effects more rapid. CB exposure increased ROS and lipid peroxidation and reduced ATP and mitochondrial membrane potential, while altering mitochondrial dynamics, mitophagy and lysosomal function. NAC treatment partially reversed oxidative, metabolic and mitochondrial alterations, supporting oxidative stress as a key mechanism of toxicity. The integrated BRI showed worsening biological condition with increasing CB concentration, and significant adverse effects were detected even at the environmentally relevant concentration of 0.03 CB/L.	*Clear concentration-dependent adverse effect, with some biphasic responses at individual metabolic endpoints.Overall rotifer health deteriorated as CB concentration increased, and BRI declined dose-dependently from major toward severe physiological alteration. However, some endpoints—notably AMS activity and, under some conditions, neutral lipid content—showed stimulation at low concentrations followed by inhibition/depletion at higher concentrations, indicating a biphasic response.*	*Not reported*

Lucia et al. 2023	Cigarette butts released a complex mixture of chemicals into seawater after 24 h. The more concentrated leachate (LEAC_A, 100 CBs/L) generally contained higher levels of several metals, PAHs, nicotine and cotinine than LEAC_B, although Cr, Ni, Pb and total aliphatic hydrocarbons did not follow this pattern; nicotine reached 180,000 µg/L in LEAC_A versus 43,200 µg/L in LEAC_B, and cotinine 1,500 versus 402 µg/L, respectively.<br>Standardized bioassays demonstrated acute toxicity: *A. fischeri* showed an EC50 of 4.47 CBs/L for both leachates; EC50 values for algal growth inhibition were 12.4/8.05 CBs/L for P. tricornutum, 4.89/5.55 for S. costatum, and 3.38/3.84 for D. tertiolecta for LEAC_A/LEAC_B, respectively. *C. gigas* was the most sensitive species and showed a marked leachate-dependent difference, with EC50 values of 0.28 CBs/L for LEAC_A versus 2.54 CBs/L for LEAC_B.<br>In the ex vivo PCTS model, viability was unchanged, but *acox1*, *gst-pi*, *cat* and *Cu/Zn-sod* transcripts decreased significantly, generally with increasing exposure time, while several other genes were unaffected. In mussels exposed directly to whole CBs, PAHs increased significantly in a dose-dependent manner and nicotine increased from below quantification in controls to 0.124, 0.266 and 3.44 µg/g at 0.5, 1 and 5 CBs/L, respectively. Biological effects became more evident at higher exposures: lysosomal membrane stability and the granulocyte/hyalinocyte ratio decreased at 5 CBs/L; micronuclei increased at 1 and 5 CBs/L; DNA fragmentation increased at 5 CBs/L; acetylcholinesterase activity decreased in haemolymph at 5 CBs/L and in gills at 1 and 5 CBs/L; ACOX increased at 5 CBs/L; glutathione decreased at all exposure levels, while glutathione reductase and total GPx increased at 5 CBs/L. The integrated Weight of Evidence (WOE) assessment showed a clear exposure-related increase in risk: Slight at 0.5 CBs/L, Moderate at 1 CBs/L and Major at 5 CBs/L.	Clear dose-dependent adverse effect / increasing integrated risk. In the whole-cigarette-butt exposure experiment, PAH and nicotine bioaccumulation and several biomarker responses increased or became significant at higher CB concentrations, and the integrated WOE classification rose from Slight → Moderate → Major at 0.5, 1 and 5 CBs/L.	The intrinsic heterogeneity of naturally smoked cigarette butts from different brands and smokers introduced variability in exposure conditions that may have influenced quantitative differences between leachates; additionally, the PCTS model was considered not particularly suitable for investigating receptor-based pathways such as Ah- and estrogen-receptor responses.
Martínez-Ruiz et al. 2026	*CB leachate significantly altered the metabolism of uninfected P. agardhii, decreasing protein and lipid content, TBARS, SOD activity, and total/intracellular microcystins, while producing a significantly higher IBRv2 than the control, consistent with CB-induced metabolic and oxidative stress. In contrast, most of these effects disappeared in chytrid-infected cultures, and IBRv2 remained comparable to the infected control, indicating that chytrid infection mitigated CB-leachate-induced stress. CB leachate also significantly reduced chytrid infection prevalence but not infection intensity. The interaction therefore differed from that observed with MET: MET + chytrids showed a synergistic stress response, whereas CB leachate + chytrids showed an antagonistic interaction. During CB exposure, nicotine decreased slightly while cotinine increased, indicating partial nicotine transformation.*	*Notb applicable*	Short experimental duration and single-time-point assessment, which may have limited the ability to capture growth dynamics and cumulative effects. In addition, because CB leachate is a complex chemical mixture, effects could not be attributed to individual constituents; nicotine and cotinine were used as indicators of leachate stability but may not represent the stability of all leachate components.
Michael et al. 2022	*Heavy-metal concentrations varied substantially according to brand, cigarette component, and smoking status. Overall, cigarette butts—particularly USB and SB samples—contained higher concentrations of several metals than cigarette/tobacco portions, although the pattern differed among individual metals and brands. Leachate analyses likewise showed marked variation, but butt-derived leachates generally*	*Not applicable*	The leaching procedure was performed in a closed system that was not designed to mimic the natural environment and instead represented a simplified

	contained higher metal concentrations than cigarette-derived leachates. For example, USB frequently showed the highest concentrations of Cu, Fe, Pb, and/or Zn, although specific rankings varied across brands. The authors also report that, overall, unsmoked cigarette materials released higher metal concentrations than smoked materials, suggesting an important contribution from residual tobacco. Some metals present in the original cigarette material were not readily detected in the leachates after 24 h, indicating differences in leachability among elements.		approach to maximize leachable metal recovery; interactions with physicochemical factors such as pH and temperature were not evaluated. The authors also note that metal concentrations may vary among cigarette brands.
Mihajlović et al. 2026	Smoked CBs contained measurable Pb, Cd, Cr and Mn, with conventional CBs showing higher mean Pb, Cr and Cd concentrations than used HEETS tobacco sticks from a heated tobacco product, whereas Mn was higher in the latter. Conventional smoked CBs contained mean concentrations of 23.37 mg/kg Mn, 8.58 mg/kg Pb, 5.80 mg/kg Cr and 0.17 mg/kg Cd; metal concentrations were lower in unused than used samples. Metal release was strongly influenced by pH, leaching medium and time, with highly acidic conditions generally enhancing leaching; Cd was mostly below detection at early time points and was preferentially released at very low pH. Leaching followed zero-order kinetics for the investigated **HEETS tobacco-stick samples**,, whereas conventional CBs showed both zero- and first-order behaviour. Health-risk modelling indicated ILCR values above the acceptable threshold for Cd and Cr in all analysed samples, with Cr-associated risk approximately 20–500 times greater than Cd-associated risk. Mn showed acceptable non-carcinogenic risk (HQ <1), whereas Pb reached HQ >1 under the highly acidic Series 2 conditions for both adults and children. Ingestion contributed substantially more to estimated risk than dermal exposure. The HEETS residues were referred to as "e-cigarette butts" in the original study, although HEETS are consumables of heated tobacco products rather than electronic cigarettes.	Metal release was pH- and time-dependent rather than showing a conventional biological dose–response relationship. Highly acidic conditions generally enhanced metal mobilisation and the associated estimated human-health risk. Cd and Cr exceeded the authors' acceptable carcinogenic-risk threshold, while Pb showed potentially unacceptable non-carcinogenic risk (HQ >1) under highly acidic conditions; Mn remained within the acceptable non-carcinogenic range.	Not reported
Mohammadi et al. 2025	Microfiber release increased significantly with exposure time, ranging from 5.88–34.77 items/g CFs at day 1 to 30.32–92.43 items/g CFs at day 60. Release was significantly greater in seawater than distilled water, whereas no significant differences were observed between cigarette brands or between laboratory and outdoor conditions. The predominant microfiber size was 20–50 µm, and white/transparent fibers were most common. Cellulose acetate represented 93% of identified polymeric fibers, followed by polyamide (5%) and polyethylene (2%). After prolonged exposure, particularly 60 days in seawater, fibers showed increased surface roughness and ruptures consistent with weathering. PLI values were >30, indicating severe pollution according to the adopted classification, while all RQ values were >1, indicating potential ecological risk. Estimated global microfiber emissions increased from 14×10^{12} at day 1 to 51×10^{12} at day 60.	Not applicable	Lack of quantitative environmental data on cigarette-filter-derived microfibers; absence of standardized methods for microplastic identification, limiting comparability across studies; visual identification restricted to fibers >20 µm, likely resu lting in substantial underestimation of released microfibers; micro-Raman analysis was not performed on all suspected fibers; and absence of standardized models for ecological and health risk assessment of microplastics.
Nitschke et al. 2023	Cigarette littering was observed in 80% of 597 smokers; younger age and being in a group increased littering likelihood, whereas greater ashtray availability reduced it. Acute cigarette-filter exposure increased *C. riparius* immobility, with significant effects from 0.25 filters/L for unsmoked filters and 1 filter/L for smoked filters. At 2 filters/L, smoked and unsmoked filters caused 36–100% and 75–100% immobility, respectively. Filter fibres generally caused greater acute immobility than leachates, and unsmoked filters were more toxic than smoked filters in fibre exposures, whereas no significant smoked–unsmoked difference was found for leachates. In the chronic	Concentration-related increase in acute toxicity, with effects occurring at lower concentrations for unsmoked than smoked filters; filter fibres generally produced greater acute toxicity than leachates. Chronic effects were more pronounced following sediment than water exposure; no concentration–	Unequal sample sizes in the cigarette-littering regression data, particularly for ashtray availability; lack of analysis of the interaction between age and group setting; greater standardization of unsmoked than randomly collected smoked filters, limiting reproducibility and comparison of their toxicity;

	assay, sediment exposure caused more pronounced effects than water exposure, including >20% higher mortality, >1.5-fold lower growth, and >80% reduced development relative to controls. Water-phase exposure significantly increased mouthpart deformity frequency and severity, with prevalence increasing from 40% in controls to 78% and 86% following smoked- and unsmoked-filter exposure, respectively. No significant differences between smoked and unsmoked filter leachates were observed for chronic endpoints.	response relationship was assessed for chronic toxicity because only one filter loading was tested.	unknown time between disposal and collection and unknown chemical load/composition of smoked filters; possible prior degradation or leaching of toxicants from collected smoked filters; limited comparability among acute experiments because the 24-h leaching period may have been insufficient to remove representative amounts of chemicals from washed fibres; absence of chemical analyses, preventing distinction between chemical and physical fibre effects; and fibre accumulation/unequal distribution within test vessels, resulting in variable direct exposure of larvae.
Nongdren et al. 2024	The annual average CBD was 0.438 ± 0.669 CBs/m², with commercial areas showing the highest littering density, followed by institutional and residential areas; winter had the highest seasonal CBD and the rainy season the lowest. The modelled WPPI for 1 CB/L was 5.22×10^{-2}; after incorporating observed littering density and estimated municipal sewage dilution, the cumulative WPPI attributable to CB littering was $(59.97 \pm 6.68) \times 10^{-6}$. Heavy metals dominated estimated exposure, contributing >99% of total CDI; Mn was the largest contributor to heavy-metal CDI. For 1 CB/L, the total HI was 1.785×10^{-2}, below the threshold of 1, whereas total modelled CR was 3.847×10^{-6}, with As contributing 76.77% of total CR, followed by Cr, Pb, naphthalene and 2-naphthylamine. After accounting for city-level sewage dilution, total CR was $<1 \times 10^{-6}$. The authors estimated that a lifetime exposure to 242 CBs/L would produce a modelled CR equivalent to smoking one cigarette per day over a lifetime	Not applicable	River dilution was not incorporated because reliable data on sewage generation, stormwater generation, and surface runoff in Aizawl were unavailable, limiting accurate estimation of the contribution of cigarette butts to downstream surface-water pollution. The precise quantity of littered cigarette butts ultimately reaching downstream water bodies was unknown, so the health-risk analysis was intended primarily to compare exposure levels rather than provide an actual population risk estimate. The contaminant-leaching dataset was restricted to studies using deionized water, although leachability may vary with pH, ionic strength, salinity, temperature, and sunlight. An ecological risk assessment could not be performed because standardized toxic-response factors were unavailable for several cigarette-butt-derived emerging contaminants.

Nongdren et al. 2026	The upstream phase was dominated by human carcinogenic toxicity, mainly from fertilizer use in tobacco cultivation and wood-fired tobacco curing, whereas cigarette consumption produced the greatest human-health burden, with sidestream smoke accounting for approximately 97% of total consumption-phase impacts. In the downstream phase, littered cigarette butts were major drivers of freshwater ecotoxicity and eutrophication, with leached nicotine and other contaminants accounting for approximately 79–82% of downstream impacts. Compared with littering, incineration, anaerobic digestion, and sanitary landfilling with gas and leachate collection generated net environmental credits, whereas open dumping and landfilling with leachate collection only retained >90% of the littering impact and produced net endpoint burdens. Recycling into fired clay bricks showed the greatest overall benefit, reducing midpoint and endpoint scores by approximately 39% and 321%, respectively. Biodegradable filters containing high proportions of flax, hemp, or jute also substantially reduced impacts; ≥90% jute- or hemp-based filters achieved the best endpoint performance.	Not applicable	Absence of complete industrial and region-specific life-cycle inventory data, requiring reliance on secondary sources and modelling assumptions; simplified representation of contaminant fate in conventional LCA, which does not adequately capture long-term cigarette-butt degradation, fragmentation, leaching, microfiber release, and dissolved organic carbon release; exclusion of some atmospheric emissions and secondary reactions during cigarette-butt degradation because of uncertainty; inability of LCA alone to represent population-level public-health burdens; and lack of real-world validation of the large-scale effectiveness of proposed end-of-life management strategies.
Olah-Kovacs et al. 2025	CB leachate adversely affected survival, growth, and reproductive performance of *L. stagnalis*, with effects evident even at the environmentally realistic concentration of 0.25 CB L^{-1}. Mortality increased with exposure concentration, while clutch number, clutch size, egg production, and hatching success declined and egg abnormalities increased. Growth responses were less clearly concentration-dependent, and feeding rate did not differ significantly among treatments during exposure. Following removal of the contamination, mortality declined, growth and feeding increased, and reproductive endpoints and egg viability approached control levels, while egg abnormalities were no longer associated with previous treatment after three weeks, indicating substantial post-exposure recovery.	A concentration-related adverse response was evident for several endpoints, particularly mortality and reproduction: increasing CB leachate concentrations were associated with reduced survival, clutch and egg production, clutch size, and egg viability, and with increased egg abnormalities. Growth and feeding responses did not show a consistent concentration-dependent pattern. Most adverse responses substantially improved after removal of exposure.	The use of CBs collected from public ashtrays prevented full standardization of cigarette-butt preparation, although this was considered to increase the ecological realism of the experiment. The chemical composition of the CB leachate was not measured, preventing attribution of the observed effects to specific contaminants. The relatively short experimental duration also limited assessment of long-term effects and repeated pollution pulses; the authors recommend longer experiments and investigation of multiple exposure fluxes.

Oliva et al. 2021	Smoked cigarette butt leachates generally produced greater ecotoxicological effects than unsmoked leachates, and marine organisms were overall more sensitive than freshwater organisms. In marine assays, smoked leachate caused concentration-dependent inhibition of microalgal growth, bacterial bioluminescence, and *Ficopomatus enigmaticus* larval development, with *Acartia tonsa* showing the greatest sensitivity; it was also the only marine model clearly responsive to unsmoked leachate. In freshwater assays, toxicity was less pronounced: *Thamnocephalus platyurus* showed no significant effects, while *Raphidocelis subcapitata* displayed biostimulation rather than growth inhibition. *D. magna* showed an unusual non-monotonic response, with greater adverse effects at intermediate smoked-leachate concentrations than at the maximum concentration. Heartbeat measurements supported a neurophysiological effect, showing significant acceleration after exposure to the higher leachate concentrations, particularly smoked leachate, with the strongest response around 75%. Overall, crustaceans emerged as particularly sensitive models for cigarette-butt-leachate toxicity.	Smoked cigarette butt leachates generally showed concentration-dependent toxicity, particularly in marine bioassays, but the dose–effect pattern was not uniformly monotonic across species. Marine algae and larval-development assays showed increasing adverse effects with increasing smoked-leachate concentration, whereas crustaceans, particularly D. magna, showed greater effects at intermediate concentrations and reduced apparent effects at the maximum concentration.	Smoked cigarette butt leachates generally showed concentration-dependent toxicity, particularly in marine bioassays, but the dose–effect pattern was not uniformly monotonic across species. Marine algae and larval-development assays showed increasing adverse effects with increasing smoked-leachate concentration, whereas crustaceans, particularly D. magna, showed greater effects at intermediate concentrations and reduced apparent effects at the maximum concentration.
Piccardo et al. 2021	Classic cigarette butts were more toxic than materials described by the original authors as “electronic cigarette butts”, and smoked classic CBs generally produced greater effects than unsmoked classic CBs. In the direct-leachate experiment, smoked classic CBs produced the highest overall toxicity (HQ = 1.99; moderate hazard), with inhibition of *A. fischeri* bioluminescence and *P. tricornutum* growth and adverse effects on *P. lividus* fertilization and larval development. The materials described as “electronic cigarette butts” were classified as absent hazard, while unsmoked classic CBs showed slight hazard. In the sediment-weathering experiment, toxicity depended strongly on weathering conditions and duration. Smoked classic CBs after 1 week under dry conditions produced the highest toxicity (HQ = 3.86; major hazard), whereas toxicity generally decreased after 2 weeks. Rain exposure also modified toxicity, with smoked CBs changing from moderate to slight hazard between weeks 1 and 2. Overall, the results indicate that combustion status, environmental weathering, and residence time strongly influence CB ecotoxicity.	Not applicable	The 10 CB/L concentration used for the direct-leachate experiment was relatively high and not environmentally relevant; the environmental representativeness of the cigarette-butt loading used in the sediment experiment was uncertain; and the high heterogeneity of electronic-cigarette devices, filters, e-liquids, nicotine contents, excipients, and flavouring agents limits the generalizability of findings for electronic cigarette butts.
Sadıç et al. 2025	Both smoked light cigarette butt and unsmoked filter leachates caused physiological, cytotoxic, and genotoxic effects in *A. cepa*, which generally increased with concentration. Root length and root number progressively decreased, with significant root-growth inhibition occurring from 25% for smoked butt leachate and from 50% for unsmoked filter leachate; at 100%, RGI declined to 0.30 and 0.32, respectively. Mitotic activity also decreased with increasing concentration: MI reductions were significant at 25–100% for smoked butt leachate and 50–100% for filter leachate. Chromosomal damage increased significantly with concentration and included fragments, bridges, chromosome stickiness, misalignment/polar deviations,	Clear concentration-dependent physiological, cytotoxic, and genotoxic effects. Increasing leachate concentrations progressively reduced root growth and mitotic activity and increased chromosomal damage. Smoked light cigarette butt leachate generally produced stronger adverse effects than unsmoked filter leachate.	Not reported

	and micronuclei. Overall chromosomal damage was greater with smoked cigarette butt leachate than with unsmoked filter leachate. Smoked-butt exposure also produced thicker, shorter, and yellowish roots, changes not observed with unsmoked-filter leachate.		
Soleimani et al. 2023	All three leachate types were acutely toxic to *P. waltoni*, with toxicity consistently following the order SCB > SF > USFand increasing with both concentration and exposure duration. LC50 values for SCB decreased from 3.75 CB/L at 24 h to 1.37 CB/L at 96 h; corresponding values decreased from 7.58 to 2.90 CB/L for SF and from 14.68 to 7.46 CB/L for USF. For SCB, mortality at 4 CB/L increased from 55% at 24 h to 95% at 96 h; for SF, mortality at 8 CB/L increased from 60% to 95%; and for USF, mortality at 16 CB/L increased from 50% to 90%. SCB leachates also contained higher concentrations of chemical contaminants than SF and USF leachates: total PAHs were 85.61, 61.48, and 50.43 µg/L, respectively, and all examined heavy metals were higher in SCB leachates. These findings indicate that residual tobacco and compounds generated or retained during smoking substantially increase the acute toxicity of cigarette-butt leachates.	Clear concentration- and time-dependent acute toxicity. Mortality increased with increasing leachate concentration and exposure duration, while LC50 decreased with increasing exposure time. Toxicity followed the order SCB > SF > USF.	The experimental conditions may have limited environmental relevance because food availability, water availability, and restricted space for movement differed from realistic environmental conditions. The study assessed acute mortality, while sublethal effects under chronic exposure were not investigated.
Srikanth et al. 2025	Cigarette butt leachate altered oxidative stress, antioxidant-defense, and neurotoxicity biomarkers in *P. virens*, with effects generally becoming stronger as leachate concentration and exposure duration increased. PC, GPx, and GSH progressively decreased across the 10%, 25%, and 50% treatments and were lower after 48 h than after 24 h. In contrast, GST increased with both concentration and exposure duration, reaching its highest activity at 50% after 48 h (381.54 ± 10.45% of control), consistent with an enhanced detoxification response. AChE activity was progressively inhibited with increasing concentration and time, with values decreasing from 92.38, 80.51, and 67.01% at 24 h to 56.39, 48.13, and 28.84% at 48 h for 10%, 25%, and 50% exposures, respectively, indicating neurotoxic effects. LPO showed the opposite pattern, increasing with concentration and exposure duration from 108.35 ± 7.56 to 216.82 ± 8.83 at 10%, 172.23 ± 13.91 to 228.69 ± 7.68 at 25%, and 218.75 ± 7.98 to 267.34 ± 3.88 at 50% between 24 and 48 h. Overall, the biomarker pattern indicates cigarette-butt-leachate-induced oxidative stress, impairment of antioxidant defenses, and neurotoxicity in *P. virens*.	Concentration- and time-dependent biological effects were observed. Increasing cigarette butt leachate concentrations and longer exposure generally resulted in greater depletion of PC, GPx, and GSH, increased GST and LPO responses, and stronger AChE inhibition, indicating progressively greater oxidative stress and neurotoxicity.	Not reported
Tan et al. 2026	Leaching tests detected Cu, Zn, Cd, total Cr, Ba, Ni, As, and cyanide, but all measured concentrations were below the corresponding hazardous-waste leaching thresholds, indicating that DCBs did not meet hazardous-waste criteria based on leaching toxicity. Chemical characterization identified 23 detectable substances among 61 investigated indicators, including metals, nicotine, formaldehyde, acetaldehyde, cyanide, and several PAHs. Nicotine concentration was 744 mg/kg. The cumulative equivalent toxicant content was 0.99, very close to the regulatory hazardous-waste threshold of 1; acutely toxic substances accounted for 90.5% of cumulative toxicity, with nicotine alone contributing 75.15%, while carcinogenic substances contributed 5.4%, mainly due to formaldehyde. The estimated acute toxicity value was 5476.08 mg/kg, compared with the regulatory acute-toxicity	Not applicable	The number and mass of DCB samples used for leaching-toxicity and toxic-substance-content analyses were lower than those specified by hazardous-waste identification standards, potentially introducing bias; subdivision of collected samples into smaller aliquots may have reduced measurement uniformity and representativeness; current analytical techniques cannot directly quantify some inorganic toxic compounds, requiring measured metal

	criterion of LD50 ≤200 mg/kg for solid waste by oral ingestion; therefore, DCBs did not meet the criterion for classification as acutely toxic hazardous waste. Overall, although the samples did not definitively satisfy the regulatory criteria for hazardous-waste classification, their toxic-substance content was very close to the threshold, indicating a potentially relevant environmental hazard, particularly if DCBs are improperly managed.		concentrations to be converted into equivalent metal-containing toxic compounds for calculation; contaminant leaching changes over time and not all compounds present in DCBs were identified and analyzed; and no comparative analysis between single-brand and mixed-brand DCBs was performed.
Torkashvand et al. 2021	The presence of CBs increased the concentrations of all five heavy metals in landfill leachate. Total concentrations of Cd, Cr, Pb, Ni, and Zn were 8.329 mg/L in the control, 8.755 mg/L with freshly smoked CBs, and 8.650 mg/L with littered CBs, corresponding to increases of 5.11% and 3.85%, respectively. Freshly smoked CBs therefore produced a greater increase in overall heavy metal contamination than littered CBs. When standardized to 1% w/w CBs, total HQ increased from 0.474 in the control to 0.524 with freshly smoked CBs and 0.491 with littered CBs, corresponding to increases of 10.52% and 3.43%, respectively. Ecological risk similarly increased from 0.269 in the control to 0.296 with freshly smoked CBs and 0.278 with littered CBs, increases of 10.05% and 3.22%. Among the mixed scenarios, increasing the proportion of freshly smoked CBs progressively increased both health and ecological risk: HQ values were 0.499, 0.508, and 0.516 and ecological-risk values were 0.283, 0.287, and 0.292 for S1, S2, and S3, respectively. Overall, freshly smoked CBs contributed more strongly than littered CBs to heavy-metal concentrations and associated risks, likely because part of the contaminant load had already leached from littered CBs before collection.	Increasing cigarette-butt contribution, particularly the proportion of freshly smoked CBs, was associated with higher heavy metal concentrations and higher estimated human-health and ecological risks. Freshly smoked CBs produced greater effects than littered CBs. However, a conventional biological dose–response relationship was not assessed.	The littered CBs collected for the study may not have been representative of actual conditions in Iranian cities. The duration of environmental exposure of littered CBs before collection could not be accurately determined, and environmental factors such as humidity can influence pollutant leaching. Consequently, the amount of pollutants remaining in littered CBs depended on previous environmental exposure and street-cleaning conditions, introducing uncertainty into comparisons between freshly smoked and littered CBs.
Venugopal et al. 2021	Experimental chemical analysis identified 34 chemicals in cigarette butt leachate, including metals, volatile organic compounds, aldehydes, and glycols; 23 were newly reported for cigarette butt leachate. Integration with literature data resulted in 98 identified chemicals, of which 25 were included in the FDA established HPHC list and four additional chemicals were proposed HPHCs. Glycerol, propylene glycol, and nicotine occurred at the highest measured concentrations. Approximately one-third of the 98 chemicals were classified as very toxic and about 10% as toxic for acute and chronic aquatic toxicity according to GHS. Acute and chronic HC50 values were available for 68 and 67 chemicals, respectively, while approximately one-third had little or no ecotoxicity information. PAHs, metals, phthalates, nicotine, and volatile organic compounds were identified as the chemical classes posing the greatest aquatic hazard; among experimentally measured compounds, nicotine, Pb, Cr, naphthalene, and limonene were among the most hazardous.	Not applicable	Cigarette butts were collected under limited environmental littering conditions and minimally represented the overall cigarette marketplace; cigarette brands and mentholated versus non-mentholated products were not controlled; some chemical constituents remained unidentified; and ecotoxicity information was unavailable for a substantial proportion of the identified chemicals.
Yang et al. 2023	A total of 10,591 CBs representing 35 brands were collected. Average CB density was highest in the park (0.124 items/m²), followed by the market (0.099 items/m²) and beach (0.077 items/m²). CB densities were significantly higher on weekends than workdays at the beach (p=0.0049) and park (p=0.0238), but not at the market (p=0.1265). In the laboratory analysis, 17 metals were detected in HongTaShan leachate and 15 in ShuangXi leachate. Total metal concentration was higher for HongTaShan (642.4 mg/L) than ShuangXi (617.1 mg/L), although individual metals differed between brands; for example, Mn was higher in	Not applicable	Not reported

	HongTaShan (395 vs 297 mg/L), whereas Pb was higher in ShuangXi (2.17 vs 0.34 mg/L). Nine PAHs were detected in both brands, with total PAHs significantly higher in HongTaShan (180.10 ± 8.56 ng/L) than ShuangXi (157.74 ± 9.19 ng/L); significant between-brand differences were observed for all detected individual PAHs except naphthalene. Microplastic fibres and nanoscale particles were also detected in leachates from both brands. Based on national cigarette-consumption data, the authors estimated that discarded CBs could generate approximately 360,000 tons of plastic waste annually in China.		
Yang et al. 2024	CBs accounted for 86.0–97.4% of total plastic litter across the sampled sites. CB density differed significantly among cities, with the highest density in Dalian (0.10 ± 0.03 CBs/m²), followed by Meizhou (0.07 ± 0.02), Baoding (0.06 ± 0.02), and Guangzhou (0.03 ± 0.02); densities in the lower-tier cities were approximately 2–3 times those in first-tier Guangzhou. Land-use-related patterns differed among cities, with significant differences in Baoding and Meizhou but not in Guangzhou and Dalian. CBPI also differed significantly among cities, with Dalian classified as high pollution (CBPI 9.0), Meizhou as pollution (3.4), and Guangzhou and Baoding as low pollution (2.0 and 1.4, respectively). Laboratory analysis demonstrated the release of fragmented and fibre-like microplastics, with heavy metals detected on some particle surfaces. Fourteen heavy metals were detected in the leachate, with a cumulative concentration of 629.7 mg/L, and Mn showed the highest concentration. Based on the observed mean CB density and laboratory leaching data, the authors estimated a potential daily release of approximately 1.9 ± 0.9 g/km² of heavy metals from littered CBs in Chinese cities.	Not applicable	Uncertainty in extrapolating the observed CB density and experimental leaching data to estimate daily heavy-metal release across Chinese cities, stating that the accuracy of the estimated 1.9 ± 0.9 g/km²/day requires further investigation and confirmation.
Yona et al. 2025	Cigarette butt abundance and characteristics differed significantly among the four beaches, with Prigi showing the highest occurrence and backshore zones generally containing more CBs than foreshore zones. Whole cigarette butts were more abundant than decayed cigarette butts, indicating continuous recent litter input. Heavy metal content and leaching behavior varied according to cigarette-butt degradation state and immersion duration. Fe tended to increase with environmental ageing in solid CBs, whereas Zn and Pb generally decreased. During seawater leaching, Cd showed rapid initial release followed by decline, Fe reached a delayed peak after 7 days, and Zn increased progressively over time, while Pb remained below the detection limit. Estimated heavy metal pollution was mainly driven by Fe and Zn and was highest at Prigi and Mutiara beaches, indicating site-specific contamination potential.	No biological dose–effect relationship was assessed.	The controlled laboratory leaching conditions and the 30-day experimental timeframe may not fully represent natural coastal dynamics. Estimated pollution values are comparative indicators of contamination potential rather than actual seawater concentrations, because natural coastal conditions are more complex than laboratory experiments. The environmental exposure history of field-collected cigarette butts was also uncertain, and some metals may already have undergone partial leaching before collection.

Note: SCBs, smoked cigarette butts; SFs, smoked filters; USFs, unsmoked filters; ΣPCBs, total polychlorinated biphenyls; DL-PCBs, dioxin-like polychlorinated biphenyls; TEQ, toxic equivalency; RQ, risk quotient; LOD, limit of detection, USCBs, unsmoked cigarette butts; CCA-NPs, commercially available cellulose acetate nanoplastics; SCB-NPs, cellulose acetate nanoplastics derived from smoked cigarette butts; USCB-NPs, cellulose acetate nanoplastics derived from unsmoked cigarette butts; MDA, malondialdehyde; NO, nitric oxide; CAT, catalase; SOD, superoxide dismutase; GPx, glutathione peroxidase; GST, glutathione S-transferase, CBL, cigarette-butt leachate; LDH, lactate dehydrogenase; GGT, gamma-glutamyl transferase; AST, aspartate aminotransferase; ALT, alanine aminotransferase; ALP, alkaline phosphatase; GSH, reduced glutathione; MDA, malondialdehyde, MFs, microfibres; EC50, median effective concentration; EC100, concentration producing 100% effect; FTIR, Fourier-transform infrared spectroscopy, VOCs, volatile organic compounds; CPMAS NMR, cross-polarization magic-angle spinning nuclear magnetic resonance; SEM-EDX, scanning electron microscopy coupled with energy-dispersive X-ray spectroscopy; PTR-ToF-MS, proton-transfer-reaction time-of-flight mass spectrometry; UT50, toxicity unit corresponding to the 50% effect threshold, CTG, CellTracker™ Green CMFDA; LC_{50}, median lethal concentration; FTIR, Fourier-transform infrared spectroscopy; GC-MS, gas

chromatography–mass spectrometry, CAF, cigarette acetate filter microplastics (non-smoked filters); GSF, ground smoked cigarette filter microplastics; LC-HRMS, liquid chromatography-high resolution mass spectrometry; OECD, Organisation for Economic Co-operation and Development; ROS, reactive oxygen species; Q-TOF, quadrupole time-of-flight mass spectrometry, nSCBs, non-smoked cigarette butts; AChE, acetylcholinesterase; LPO, lipid peroxidation; GR, glutathione reductase; TG, total glutathione; GSH, reduced glutathione; GSSG, oxidized glutathione, ΣPAHs, total polycyclic aromatic hydrocarbons; EPA, United States Environmental Protection Agency; WFD, Water Framework Directive; GC-MS, gas chromatography–mass spectrometry, BTEX, benzene, toluene, ethylbenzene, o-xylene and p-xylene; DW, distilled water; RW, river water; HS-GC-MS, headspace gas chromatography–mass spectrometry; KOC, organic carbon–water partition coefficient; Kwater/air, water–air partition coefficient, ΣAAs, total aromatic amines; ANL, aniline; 1-NA, 1-naphthylamine; 2-NA, 2-naphthylamine; 2,6-DMA, 2,6-dimethylaniline; o-ASD, o-anisidine; p-TOL, p-toluidine; m-TOL, m-toluidine; o-TOL, o-toluidine; 3-ABP, 3-aminobiphenyl; 4-ABP, 4-aminobiphenyl; LC–MS/MS, liquid chromatography–tandem mass spectrometry, ΣPAAs, total primary aromatic amines; PAAs, primary aromatic amines; ANL, aniline; ΣTOL, total toluidines; LC–MS/MS, liquid chromatography–tandem mass spectrometry, PSL = polystyrene latex; NPTf = fragmented polystyrene nanoplastics; AF4 = asymmetric flow field-flow fractionation; KSW = cigarette butt–water partition coefficient; Rs = water sampling rate; XDLVO = extended Derjaguin–Landau–Verwey–Overbeek, CEI = Clean Environment Index; CBPI = Cigarette Butt Pollution Index; DCB = density of cigarette butts; RICs = resin identi fication codes, PCTS = Precision-Cut Tissue Slices; WOE = CB-MF = cigarette butt-derived microfiber; SSI = skin saturation index; CI = chromatophore index.Weight of Evidence; LOE = Line of Evidence; LEAC_A = leachate prepared at 100 CBs/L; LEAC_B = leachate prepared at 25 CBs/L, FCB = Freshly Smoked Cigarette Butt; WCB = Whole Cigarette Butt; DCB = Decayed Cigarette Butt; EP = Estimated Pollution; ICP-OES = Inductively Coupled Plasma Optical Emission Spectrometry, MDL = Method Detection Limit; PC = Polycarbonate, GHS = Globally Harmonized System of Classification and Labelling of Chemicals; HC50 = Hazardous Concentration affecting 50% of species; SSD = Species Sensitivity Distribution; HPHCs = Harmful and Potentially Harmful Constituents, CDI = Chronic Daily Intake; RfD = Reference Dose, PC = Protein Carbonyl; GPx = Glutathione Peroxidase; GSH = Reduced Glutathione; GST = Glutathione S-Transferase; AChE = Acetylcholinesterase; LPO = Lipid Peroxidation, SCB = Smoked Cigarette Butt with tobacco; SF = Smoked Filter without tobacco; USF = Unsmoked Filter; LC50 = Lethal Concentration 50; PAHs = Polycyclic Aromatic Hydrocarbons, GI = Germination Index; RGI = Relative Growth Index; MI = Mitotic Index; EMS = Ethyl Methanesulfonate, DCB = discarded cigarette butt; ATE = acute toxicity estimate; L/S = liquid-to-solid ratio; QA/QC = quality assurance/quality control; RSD = relative standard deviation; MSDS = Material Safety Data Sheet; LOD = limit of detection, CCB = classic cigarette butt; ECB = electronic cigarette butt; S-CCB = smoked classic cigarette butt; U-CCB = unsmoked classic cigarette butt; S-ECB = smoked electronic cigarette butt; U-ECB = unsmoked electronic cigarette butt; ASW = artificial seawater; NSS = natural sandy sediment; HQ = Hazard Quotient; WOE = Weight of Evidence; PLA = polylactic acid, USC = unsmoked cigarette; SC = smoked cigarette; USB = unsmoked butt; SB = smoked butt; AAS = atomic absorption spectroscopy, MET = metolachlor; IBRv2 = Integrated Biomarker Response version 2; MC = microcystin; TBARS = thiobarbituric acid reactive substances; SOD = superoxide dismutase; GST = glutathione S-transferase, SCB = smoked cigarette butt with residual tobacco; SF = smoked cigarette filter without residual tobacco; USF = unsmoked cigarette filter; BRI = integrated biomarker response index; NAC = N-acetylcysteine, RGP = Relative Germination Percentage; GI = Germination Index; RRG = Relative Radicle Growth; HG = Hypocotyl/Coleoptile Growth, SG = seed germination; RSG = relative seed germination; RRG = relative radicle growth; GI = germination index.
