# Supplementary material for: Toxicants Released from Cigarette Butt and Other Tobacco and Nicotine Product Waste and Their Ecotoxicological Impact: A Systematic Review with One Health Perspectives
Source: Toxics. 2026 Sep 23;14(10):846. doi: 10.3390/toxics14100846 (PMC13709674; doi:10.3390/toxics14100846)
Supplement: Supplementary file 1 [file toxics-14-00846-s001.zip › Table S1.pdf]

Table S1. Review-specific methodological quality assessment checklist for laboratory experimental studies that are neither in vivo nor in vitro.

Domain	Assessment criterion	Low methodological concern (2)	Moderate methodological concern (1)	High methodological concern (0)
1. Study objectives	Are the study objectives clearly stated and consistent with the study design?	Objectives clearly defined and fully aligned with the design	Objectives partially described or only partially aligned	Objectives unclear or inconsistent
2. Study design	Is the selected study design appropriate to answer the research question?	Appropriate and fully justified	Appropriate but justification limited	Inappropriate or poorly described
3. Sampling strategy	Is the sampling strategy adequately described and representative of the study area or population?	Representative and clearly described	Minor limitations	Poorly described or likely to introduce selection bias
4. Exposure characterization	Is exposure adequately measured, estimated or characterized?	Direct measurements or well-validated models	Combination of measured and secondary data	Poorly described or based on unsupported assumptions
5. Analytical methods	Are laboratory, analytical or measurement methods adequately described and validated?	Fully validated methods with quality assurance/control	Methods described but validation incomplete	Methods inadequately described or not validated
6. Input data quality	Are the data sources reliable and appropriate?	High-quality primary data or well-justified secondary sources	Combination of reliable and uncertain sources	Poor-quality or undocumented data

7. Model assumptions (when applicable)	Are modelling assumptions explicitly reported and scientifically justified?	Clearly stated and justified	Partially justified	Not reported or unsupported
8. Uncertainty and sensitivity analysis (when applicable)	Is uncertainty appropriately evaluated?	Comprehensive uncertainty and/or sensitivity analysis performed	Limited uncertainty assessment	No uncertainty assessment
9. Statistical/modelling analysis	Are statistical or modelling methods appropriate and adequately reported?	Appropriate, reproducible and correctly interpreted	Minor methodological limitations	Inappropriate or insufficiently described
10. Interpretation of findings	Are conclusions supported by the results and are study limitations adequately discussed?	Conclusions fully supported and limitations clearly acknowledged	Minor overinterpretation or incomplete discussion	Conclusions not supported or limitations ignored

Note. Each domain was rated as low methodological concern (2 points), moderate methodological concern (1 point), or high methodological concern (0 points). Domains 7 and 8 were considered applicable only when a study directly developed or implemented a quantitative modelling or risk-modelling framework involving explicit assumptions. Simple descriptive calculations, pollution indices, concentration estimates, or the use of model-derived parameters or toxicity metrics obtained from previously published sources were not, by themselves, considered sufficient to classify a study as a modelling study. For non-modelling studies, Domains 7 and 8 were rated as not applicable (N/A) and excluded from the total score. For studies with all ten domains applicable, overall methodological quality was classified as high (17–20), moderate (13–16), or low (≤12). For non-modelling studies (Domains 7–8 = N/A), methodological quality was classified as high (14–16), moderate (10–13), or low (≤9).

Overall methodological quality

Total score	Overall methodological quality
17–20	High
13–16	Moderate
≤12	Low

Scoring for non-modelling studies (Domains 7–8 = N/A)

Total score	Overall methodological quality
14–16	High
10–13	Moderate
≤9	Low
