# Supplementary material for: Toxicants Released from Cigarette Butt and Other Tobacco and Nicotine Product Waste and Their Ecotoxicological Impact: A Systematic Review with One Health Perspectives
Source: Toxics. 2026 Sep 23;14(10):846. doi: 10.3390/toxics14100846 (PMC13709674; doi:10.3390/toxics14100846)
Supplement: Supplementary file 1 [file toxics-14-00846-s001.zip › Table S2a methodological quality assessment of vitro studies .pdf]

Table S2a. ToxRTool reliability assessment of the in vitro components of the included studies (N=7)

Authors, Year	Criteria I	Criteria II	Criteria III	Criteria IV	Criteria V	Score	
Bonanomi et al., 2026	4	3	6	3	2	18/18	Reliable without restriction
Guttmann et al., 2024	3	3	6	3	2	17/18	Reliable without restriction
Jakimiuk et al., 2022	3	3	6	3	2	17/18	Reliable without restriction
Lazo et al., 2024	4	3	6	3	2	18/18	Reliable without restriction
Michael et al., 2022	3	3	6	3	2	17/18	Reliable without restriction
Martinez – Ruiz et al., 2026	2	3	6	3	2	16/18	Reliable without restriction
Piccardo et al., 2021	3	3	6	3	2	17/18	Reliable without restriction
