# Supplementary material for: Toxicants Released from Cigarette Butt and Other Tobacco and Nicotine Product Waste and Their Ecotoxicological Impact: A Systematic Review with One Health Perspectives
Source: Toxics. 2026 Sep 23;14(10):846. doi: 10.3390/toxics14100846 (PMC13709674; doi:10.3390/toxics14100846)
Supplement: Supplementary file 1 [file toxics-14-00846-s001.zip › Table S2b methodological quality assessment of in vivo studies.pdf]

Table S2b. ToxRTool reliability assessment of the in vivo components of the included studies (N=20).

**Authors, Year**	**Criteria I** Test substance identification	**Criteria II** Test system characterisation	**Criteria III** Study design description	**Criteria IV** Study results documentation	**Criteria V** Plausibility of study design and results	**Score**	
Bakr et al., 2025	4	2	7	3	2	**18/21**	**Reliable without restriction**
Banaee et al., 2024	4	3	7	3	2	**19/21**	**Reliable without restriction**
Belzagui et al., 2021	4	3	6	3	2	**18/21**	**Reliable without restriction**
Caridi et al., 2020	4	3	7	3	2	**19/21**	**Reliable without restriction**
Cho et al., 2025	4	4	7	3	2	**20/21**	**Reliable without restriction**
Couto et al., 2025	4	3	7	3	2	**19/21**	**Reliable without restriction**
Ferreira-Griz et al., 2026	2	4	6	3	2	**17/21**	**Reliable with restriction**
Dolar et al., 2025	4	4	7	3	2	**20/21**	**Reliable without restriction**
Dos Santos et al., 2024	4	3	6	3	2	**18/21**	**Reliable without restriction**

Green et al., 2020	2	3	7	3	2	**17/21**	**Reliable with restriction**
Green et al., 2023	3	2	7	3	2	**17/21**	**Reliable with restriction**
Kim et al., 2023	4	3	7	3	2	**19/21**	**Reliable without restriction**
Lian et al., 2025	4	3	7	3	2	**19/21**	**Reliable without restriction**
Lucia et al., 2023	3	3	7	3	2	**18/21**	**Reliable without restriction**
Nitschke et al., 2023	2	4	7	3	2	**18/21**	**Reliable without restriction**
Olah-Kovacs et al., 2025	4	4	7	3	2	**20/21**	**Reliable without restriction**
Oliva et al., 2021	4	3	7	3	2	**19/21**	**Reliable without restriction**
Sadic et al., 2025	2	2	7	3	2	**16/21**	**Reliable with restriction**
Soleimani et al., 2023	4	4	7	3	2	**20/21**	**Reliable without restriction**
Srikanth et al., 2025	4	3	6	3	2	**18/21**	**Reliable without restriction**

Note. Each criterion was scored as 1 (criterion met) or 0 (criterion not met). The overall reliability classification was determined according to the ToxRTool scoring system, taking into account both the total score and the fulfilment of the minimum information requirements (“red criteria”). For studies combining multiple study designs or experimental approaches, the assessment reported in this table refers exclusively to the in vivo component; other components were evaluated separately using the corresponding study-design-specific assessment approach.
