# Supplementary material for: Toxicants Released from Cigarette Butt and Other Tobacco and Nicotine Product Waste and Their Ecotoxicological Impact: A Systematic Review with One Health Perspectives
Source: Toxics. 2026 Sep 23;14(10):846. doi: 10.3390/toxics14100846 (PMC13709674; doi:10.3390/toxics14100846)
Supplement: Supplementary file 1 [file toxics-14-00846-s001.zip › Table S3 methodological quality assessment of environmental and non-in vivonon-in vitro studies quality assessment .pdf]

Table S3. Methodological quality assessment of environmental and non-in vivo/non-in vitro studies (N=22).

Study	D1	D2	D3	D4	D5	D6	D7	D8	D9	D10	Total score	Overall quality
Arfaeinia et al., 2024	2	2	1	2	2	2	1	0	2	2	**16/20**	**Moderate**
Dobaradaran et al., 2020	2	2	1	2	2	2	na	na	2	1	**14/16**	**High**
Dobaradaran et al., 2021	2	2	1	2	2	2	na	na	2	1	**14/16**	**High**
Dobaradaran et al., 2022	2	2	1	2	2	2	na	na	2	1	**14/16**	**High**
Dobaradaran et al., 2023	2	2	1	2	2	2	1	0	2	1	**15/20**	**Moderate**
Dobaradaran et al., 2024a	2	2	1	2	2	2	1	0	2	1	**15/20**	**Moderate**
Dobaradaran et al., 2024b	2	2	1	2	2	2	1	0	2	1	**15/20**	**Moderate**
El Hadri et al., 2021	2	2	1	2	2	2	2	0	2	2	**17/20**	**High**
Garshasbi et al., 2026	2	2	1	2	1	2	na	na	1	1	**12/16**	**Moderate**
Kouhi et al., 2025	2	2	1	1	1	1	1	0	2	1	**12/20**	**Low**
Michael et al., 2022	2	2	1	2	1	2	na	na	1	1	**12/16**	**Moderate**
Mihajlović et al., 2026	2	2	1	2	2	2	1	0	1	1	**14/20**	**Moderate**
Mohammadi et al., 2025	2	2	1	2	1	2	1	1	2	2	**16/20**	**Moderate**
Nitschke et al., 2023†	2	2	1	2	1	2	na	na	2	2	**14/16**	**High**
Nongdren et al., 2024	2	2	2	1	1	1	1	2	2	1	**15/20**	**Moderate**
Nongdren et al., 2026	2	2	1	2	2	1	1	2	2	2	**17/20**	**High**
Tan et al., 2026	2	2	0	2	2	2	1	0	2	2	**15/20**	**Moderate**
Torkashvand et al., 2021	2	2	1	2	1	2	1	0	1	1	**13/20**	**Moderate**
Venugopal et al., 2021	2	2	1	2	2	1	na	na	2	2	**14/16**	**High**
Yang et al., 2023‡	2	2	2	2	1	2	na	na	1	1	**13/16**	**Moderate**
Yang et al., 2024	2	2	2	2	2	2	na	na	2	1	**15/16**	**High**
Yona et al., 2025	2	2	2	2	2	2	na	na	2	2	**16/16**	**High**

Note. D1, Study objectives; D2, Study design; D3, Sampling strategy; D4, Exposure characterization; D5, Analytical methods; D6, Input data quality; D7, Model assumptions; D8, Uncertainty and sensitivity analysis; D9, Statistical/modelling analysis; D10, Interpretation of findings. Scores: 2 = low methodological concern; 1 = moderate methodological concern; 0 = high methodological concern; na = not applicable. For mixed-design studies including observational/environmental and in vivo or in vitro components, only the observational/environmental component was assessed using this checklist; the in vivo or in vitro component was assessed separately using the corresponding study-design-specific quality assessment tool.

† Nitschke et al. (2023): the score is based exclusively on the observational cigarette-littering component; the in vivo ecotoxicological component was assessed separately.

‡ Yang et al. (2023): the score reported here refers only to the observational/environmental component; the laboratory in vivo/in vitro component, where applicable, was assessed separately using the corresponding tool.
